# Timely community palliative and end-of-life care: a realist synthesis

**DOI:** 10.1136/bmjspcare-2021-003066

**Published:** 2021-12-09

**Authors:** Mila Petrova, Geoff Wong, Isla Kuhn, Ian Wellwood, Stephen Barclay

**Affiliations:** 1 Palliative & End of Life Care in Cambridge (PELiCAM) Research Group, Primary Care Unit, Department of Public Health and Primary Care, University of Cambridge, Cambridge, UK; 2 Nuffied Department of Primary Care Health Sciences, University of Oxford, Oxford, UK; 3 Medical Library, University of Cambridge, Cambridge, UK; 4 Primary Care Unit, Department of Public Health and Primary Care, University of Cambridge, Cambridge, UK

**Keywords:** clinical decisions, home care, prognosis, terminal care

## Abstract

**Background:**

Community-based and home-based palliative and end-of-life care (PEoLC) services, often underpinned by primary care provision, are becoming increasingly popular. One of the key challenges associated with them is their timely initiation. The latter requires an accurate enough prediction of how close to death a patient is.

**Methods:**

Using ‘realist synthesis’ tools, this review sought to develop explanations of how primary care and community PEoLC programmes generate their outcomes, with the explanations presented as context–mechanism–outcome configurations. Medline, Embase, CINAHL*,* PsycINFO*,* Web of Science*,* ASSIA*,* Sociological Abstracts and SCIE Social Care Online were originally searched. A multistage process of focusing the review was employed, with timely identification of the EoL stage and timely initiation of associated services representing the final review focus. Synthesised sources included 21 full-text documents and 324 coded abstracts, with 253 ‘core contents’ abstracts generating >800 codes.

**Results:**

Numerous PEoLC policies and programmes are embedded in a framework of Preparation and Planning for Death and Dying, with identification of the dying stage setting in motion key systems and services. This is challenged by: (1) accumulated evidence demonstrating low accuracy of prognostic judgements; (2) many individuals’ orientation towards Living and Hope; (3) expanding grey zones between palliative and curative care; (4) the complexity of referral decisions; (5) the loss of pertinent information in hierarchical relationships and (6) the ambiguous value of having ‘more time’.

**Conclusion:**

Prioritising temporal criteria in initiating PEoLC services is not sufficiently supported by current evidence and can have significant unintended consequences.

**PROSPERO registration number:**

CRD42018097218.

**Key messages**

**What was already known?**

- The initiation of palliative and end-of-life care services often requires a judgement that a patient is approaching death, whether it is within days, weeks, months or a year. Such prognostic judgements are acknowledged as irreducibly uncertain but accepted as sufficiently reliable in the majority of cases.

**What are the new findings?**

- A significant body of evidence from systematic reviews suggests that judgements of proximity of death often have accuracy below chance levels. Furthermore, a range of unintended consequences follow from making the identification of the end-of-life stage central to providing palliative and end-of-life care.

**What is their significance?**

- Clinical
  - Relative to current evidence, we need to consider the implications of softening, potentially even removing, time-based criteria from the sets of referral criteria for palliative and end-of-life care services.
- Research
  - Priority needs to be given to research syntheses on: consequences of inaccurate predictions of approaching death; the reception of ‘bad news’ relative to findings about significant background uncertainty of prognosis; the effectiveness of second-line and third-line therapies; and the relationship between early referral for home care and home death.

## Background

 ‘How long have I got left, doctor?’ is perhaps the scariest and most courageous question a patient can ask in the context of healthcare. It is also one of the most difficult for a health professional to answer, both because of the uncertainty of prediction and the intensity of emotions the latter tends to evoke. The judgement underpinning the response to the above question—a specific, even if irreducibly uncertain, temporal prognosis or a more general expectation that a patient is in the last stage of their life—is also central to health professionals’ considering transitions from curative to palliative and end-of-life care (PEoLC). It is often a key formal criterion for initiating PEoLC services.

This paper brings together evidence and lines of argumentation around the timely (or otherwise) identification of patients at the end-of-life and the timely (or otherwise) initiation of PEoLC services. This was the ultimate focus of a realist review on PEoLC programmes in primary care and/or community settings in England. The trajectory through which this final focus was arrived at is, arguably, consequential for the answers the review proposes, primarily by virtue of the sample of the literature included in it and the lens through which the latter was analysed. The original framing of the review is thus preserved as background to the study, even if the questions it provides answers to are both narrower (related to timely identification of dying patients and timely initiation of relevant services rather than PEoLC programmes in primary care and the community overall) and broader (not restricted geographically and with a relevance beyond primary and community care).

At a global level, a 2014 resolution of the World Health Assembly^1^ urged member states to ‘integrate evidence-based, cost-effective and equitable palliative care services in the continuum of care, across all levels, with emphasis on primary care, community-based and home-based care, and universal coverage schemes’. A more recent (2018) WHO document reasserted and specified this commitment further and also offered a basic model of such integration.^2^ Regional position papers, reports, research and related documents have been developed in parallel or followed on, both in high-income^3 4^ and low- and middle-income regions.^5 6^ In the context of England, whose PEoLC policy documents were central to this study, the six ‘ambitions’ for PEoLC include a community-centred ambition recognising that ‘dying, death and bereavement are not primarily health and social care events; they affect every aspect of people’s lives and experience’.^7^ In the context of the country’s primary care, the most recent general practitioner (GP) contract, in its first year (2019/2020), enabled practices to achieve 37 ‘Quality and Outcomes Framework’ points by engaging in continuous quality improvement of their end-of-life care provision,^8^ a leap from 6 points pre-2019.^9^ In view of the growing importance, complexity and context-dependence of primary and community care programmes for PEoLC, we sought to elucidate, using the principles of realist research, the mechanisms through which such programmes generate their outcomes in the immense variety of contexts in which they are designed and implemented.

## Methods

### Realist research and realist synthesis

Realist syntheses (or realist reviews, with the terms generally used interchangeably) are a form of theory-based literature reviews belonging to the field of ‘realist research’ as pioneered by Pawson and Tilley.^10^ The realist synthesis approach has been specified in the scholarship of Pawson;^11^,^14^ codified in the outputs of the RAMESES projects (Realist And Meta-narrative Evidence Syntheses: Evolving Standards)^15^,^17^; and has been ramifying in the work of the latter’s team members and a growing number of committed adopters. The approach is also actively contested and advanced through the RAMESES mailing list^18^ and international realist research conferences.^19^ Work belonging or claiming to belong to the realist domain has appeared in areas as diverse as ‘development studies, social care, public health, crime reduction, agricultural extension, information science, wildfire prevention’.^13^ High level policy and funding circles are becoming increasingly receptive to it, including government departments and agencies, such as the UK Treasury,^20^ the UK Department for International Development and the US Agency for International Development,^21^ and multinational organisations such as the World Bank and WHO.^21^

Realist reviews are one of a significant number of approaches (over 30 as per^22^) developed in the 1990s/early 2000s in response to limitations of the mainstream, Cochrane type, systematic reviews. Basic bibliometric searches in PubMed show it as one of four most widely used ‘alternative’ synthesis methods (see online supplemental appendix 1). Realist synthesis ‘always has explanatory ambitions’, is ‘firmly rooted in a realist philosophy of science and places particular emphasis on understanding causation and how causal mechanisms are shaped and constrained by social context’.^17^ A realist synthesis question is a (usually partial) version of the question ‘What works, how, why, for whom, to what extent and in what circumstances, in what respect and over what duration?’ (*ibid*.). This question is one representation of a fundamental assumption of realist research that ‘programme effectiveness will always be partial and conditional’ (*ibid*.). The outcome of a realist review is a set of middle-range theories aiming to explain how programmes cause their outcomes. The resulting causal explanations take the form of contexts–mechanisms–Outcomes (CMO) configurations. Data in a realist review are relevant if they contribute to the development, refinement and testing of programme theories and can thus come from a broad variety of sources, study types and thematic domains, including ones far removed from the topic being studied. Risk of bias is judged for the sets of evidence and arguments used in the synthesis and is not attributed to the source study type and/or the cumulative quality of its execution.^11^,^15^ Online supplemental appendix 1 outlines further features of the realist approach.

### Review questions

The original questions of the review were as follows (see PROSPERO protocol CRD42018097218^23^ for details):

1. What are the key mechanisms which underpin PEoLC programmes for adults in primary care and community settings in the UK and similar healthcare systems?
2. How do different contexts set in motion or block those programme mechanisms?
3. What other enabling and blocking mechanisms have an impact on the outcomes of community PEoLC services?
4. How do outcomes differ across patient groups, context types and time points in the patient journey?

For realist reviews, it is ‘typical and legitimate’ for the review objectives, questions, breadth and depth to evolve as the review progresses.^17^ After iterative focusing (described below), we sought to offer a high-level conceptualisation in response to the above questions and answer in detail the following more specific questions:

- What are the key mechanisms which underpin timely (or otherwise) *identification* of patients at the end of life who may benefit from PEoLC programmes in primary care and/or community settings?
- What are the key mechanisms which underpin timely (or otherwise) initiation of relevant services?
- How do different contexts set in motion or block those mechanisms?
- How do key patient-focused, carer-focused, staff-focused and health system-focused outcomes, namely ‘good death’, quality of care, cost-effectiveness, coordination of care, hospital admissions and place of death, differ as a result of differences in the timing of identification of end-of-life stage and/or initiation of relevant services?

‘Identification’ of patients at the end of life was understood, broadly, to denote a judgement made by a healthcare professional that a patient is likely to be approaching the end of life, whether this is the last year, months, weeks or days of life, followed by adjustments to the course of care for that patient.

‘Initiation’ of PEoLC services was understood, broadly, to mean the first step that makes the transition from a mainly curative focus of care to a palliative and/or end-of-life care emphasis on comfort and quality of life.

### Review processes and stages

#### Development of rough programme theory and study protocol

A key task at the outset of a realist study is the constructing, or ‘surfacing’, of an initial (rough, candidate) programme theory.^24 25^ ‘Programme theory’ is the description ‘of what is supposed to be done in a policy or programme (theory of action) and how and why that is expected to work (theory of change)’.^24^ At the end of a realist review, a programme theory needs to be couched in CMO terms and serve to explain ‘how and why different outcomes are generated in different contexts’.^24 25^ We developed an initial programme theory primarily through within-team discussions (diagrammatic representation in online supplemental appendix 2). Two team members were content experts in PEoLC (SB and MP), two (SB and GW) were practising GPs, and one team member (GW) was an experienced realist reviewer (the other two core team members, IK and IW, were, respectively, a library and information specialist and an evidence synthesis researcher with a clinical background). Foregrounding conceptual challenges in the discussions were how to circumscribe ‘a programme’ and how broadly to cast the net in defining ‘community’ (see box 1 on the understanding of ‘community programme’ consolidated in the course of the work). Key UK policy documents on palliative, end-of-life care and community care were also consulted. Two stakeholder groups were engaged: a study-specific professionals’ advisory group and a patient and public involvement (PPI) Group with a broader remit (detail in online supplemental appendix 3).

Box 1 **What is a community programme?**

**Treatment of key types of boundary cases:**

Home-based, day care and other non-inpatient programmes delivered by hospital, hospice or specialist palliative care teams were included; care home programmes were included.

**Substance—surface tension:**

As only one of the above features was sufficient to identify a programme as a ‘community programme’, some of the initiatives we have included would not be considered ‘proper’ community programmes under stricter definitions, that is, ones aiming to exclude all cases of ‘tokenistic’ involvement.

**Local—global perspective:**

In the UK and other high-income countries, social institutions are, overall, better established and stronger than those in low- and middle-income countries. This is often believed to be paralleled by greater independence of the members of a given community from one another and of the community as a whole. As such, the community programmes we reviewed may have, on average, stronger formal structures and links with social institutions than would be the case in more traditional societies. They might also be less influential by virtue of having, typically, a range of statutory alternatives.

#### Literature searching and initial screening

Online supplemental appendix 4 describes in detail the approach to literature searching and screening. Briefly, our main search strategy combined four blocks of search terms around: (1) PEoLC; (2) primary and community care; (3) UK (which reduced non-UK sources but still captured a significant number of them) and (4) programme (theory, model, philosophy). Limiting to UK sources served to circumscribe, in a way that ensured coherence of the macrocontext, the policy, health services setup, financial and community context of programmes. However, no restrictions on origin or language of papers were placed. As long as they helped to test and refine the evolving programme theory, they were included for further consideration. We searched Medline, Embase, CINAHL*,* PsycINFO*,* Web of Science*,* ASSIA*,* Sociological Abstracts and SCIE Social Care Online. Records between 1998 and 2018 were used, 10 years either side of the 2008 End-of-Life Care Strategy for England.^26^ The resulting dataset was of 2832 citations.

Relevance of a piece of evidence in the realist approach is determined by its capacity to enable the testing and refinement of the programme theory. As the theory itself is under development, judgements about relevance are partly dynamic and underdetermined. While we were guided by the set of inclusion-exclusion criteria outlined in the protocol^23^ (briefly: adults whose death is perceived as imminent or who have advanced, progressive or incurable conditions; programmes, interventions, initiatives, approaches, tools, etc for the provision of PEoLC in primary care and other community settings; no restrictions on study design, non-empirical research also included), we worked within flexible boundaries of relevance. In the screening process, we classified abstracts into tiered inclusion and exclusion categories, reflecting different levels of perceived relevance (eg, ‘core contents’, ‘include, generic’, ‘include, broad’). The ‘include, broad’ category, in particular, contained references that went beyond our explicit inclusion criteria (eg, from other countries, settings, conditions, age groups) but pointed to potentially transferable CMO-elements or configurations. A range of targeted searches were also conducted as the study progressed (see online supplemental appendix 4).

#### Data extraction, analysis and synthesis

After screening 1226 citations (title, abstract, keywords; alphabetical order of first author surname within chronological order) out of the main dataset (of 2832 citations), we reached a level of saturation of emerging issues. This was taken as an opportunity to use roughly half of the dataset for theory development and refinement and the other half for theory testing. Data extraction—in the form of document coding—was then initiated. Using NVivo (QSR International, V.12), we coded with a high level of granularity the abstracts of all citations tagged as ‘core contents’ or ‘potentially core contents’ during the screening (253 citations, over 800 codes). To achieve greater accuracy, systematicity and transparency of the process of extracting data from the primary studies and including them into the synthesis study, we annotated substantive codes with what we called ‘bridging terms’. The latter linked the form in which the data appeared in the original study to the CMO configurations expected as an outcome of a realist study. For instance, MMEAN—standing for “Mechanisms, Meanings, Experiences, Attitudes, Narratives’—indicated that the original study relied on concepts such as ‘meaning’ or ‘experiences’ and that its findings can underpin the formulation of a realist mechanism.

Next, we reorganised the NVivo codes into eight broad categories representing types of PEoLC programmes (see Findings). We sought to develop categories by abstracting high level CMOs from the specific CMOs characterising the programmes described in the literature. Apart from taking the analysis at a higher conceptual, realist-informed, level, this was an attempt to contain and focus the work. Further narrowing of the review focus was, however, required. The steps taken to achieve it aimed to balance the following tensions:

1. Clear focus—preservation of aspects of the big picture envisaged in the original review questions.
2. Priorities in policy documents—priorities in the research literature.
3. Practical importance—conceptual promise.
4. Relevance to the practice of professionals—resonance with lay persons.
5. Richness of data—feasibility of their analysis.

The time and timing theme was chosen as a focus for the review, as it met the five criteria above better than any other candidate theme. As, on closer inspection of the main dataset, we found that time and timing was still too broad a topic, we went through a succession of further stages in narrowing the review focus. We mapped discussions of time and timing in the retrieved research literature against discussions of the same concepts in five key national policy documents and/or reports.^726^,^29^ We took the overlap of interest to represent shared priorities, including:

1. Timely identification of patients who are approaching the end of life, typically seen as prompting the timely initiation of relevant services.
2. What we termed ‘temporally defined services’ (such as 24/7 services, rapid response services, out of hours, night sitting, fast track discharge).
3. Advance care planning.
4. The final race against time to respect a dying patient’s wishes.

The decision to focus on identification (topic 1 of the four above) was driven by the relative richness of evidence in our dataset (higher for topics 1 and 2 than for 3 and 4); the fact that identification of the end-of-life stage was the first step in a new pathway of care; and, finally, the strong negative reaction we received to some of the findings from the PPI Group, perceiving them as damaging to the need and right to be appropriately prepared for death, should health professionals be aware of an impending death. We felt that finding clear and effective ways to represent this subset of the study findings is important. See online supplemental appendix 3 for details on stakeholder involvement.

**Figure 1:**
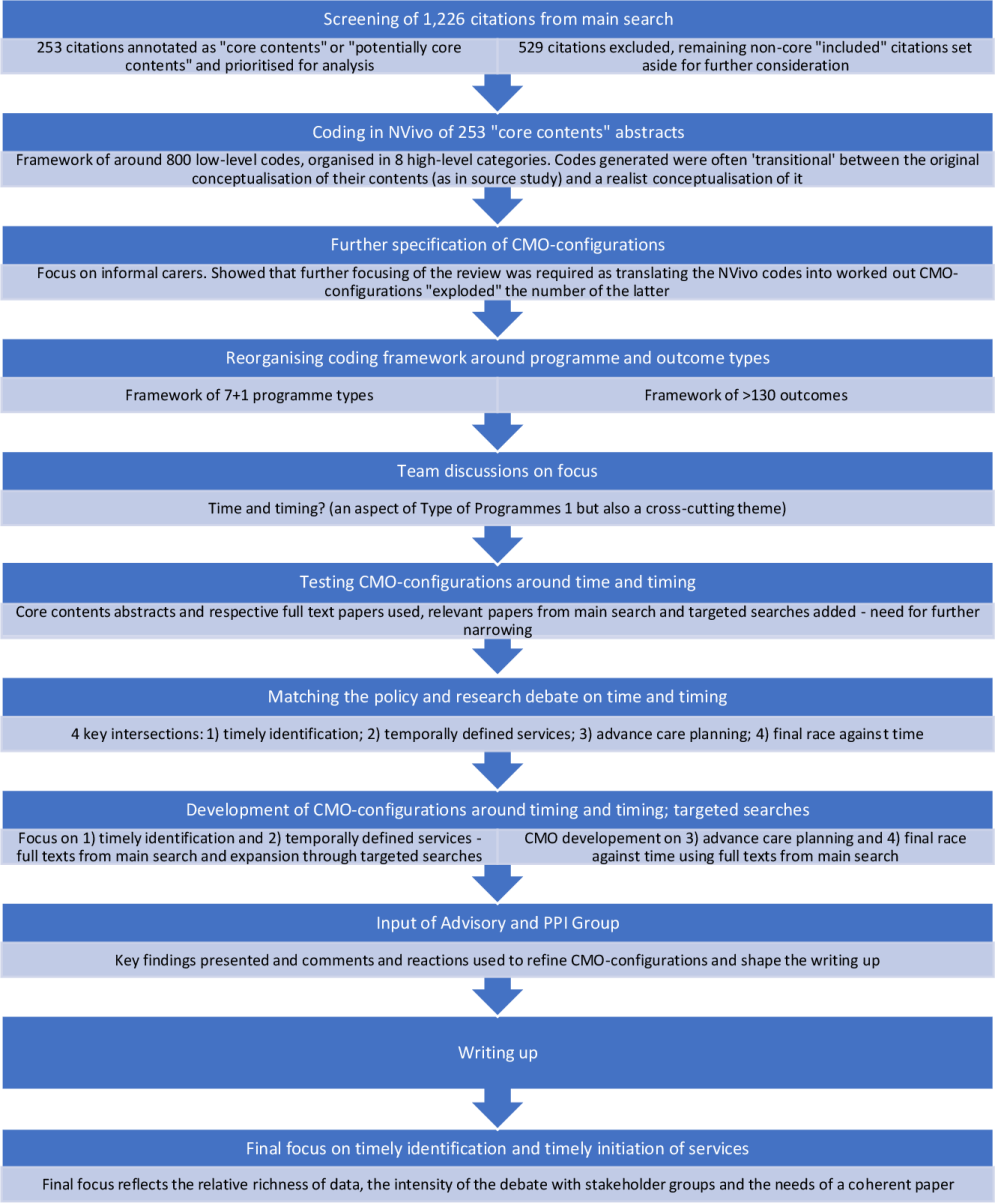
Stages in narrowing the review focus, intertwined with stages in the analysis. CMO, context–mechanism–outcome.

**Figure 2:**
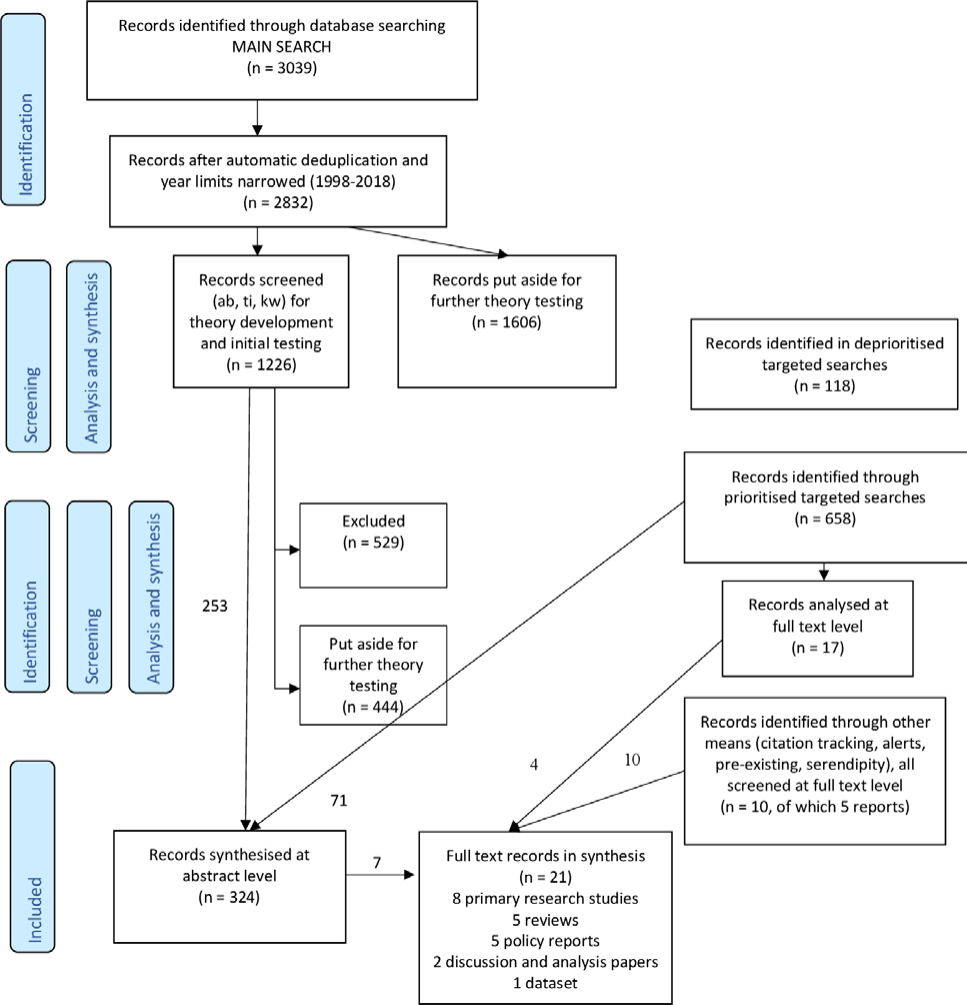
Modified PRISMA flow diagram. PRISMA, Preferred Reporting Items for Systematic Reviews and Meta-Analyses.

Figure 1 represents graphically the process of focusing the review, including sub-stages not discussed above. Figure 2 represents a modified PRISMA diagram.

## Findings

The Findings section consists of two largely independent parts. Box 2 represents a classification of PEoLC programmes in terms of their overarching logic of mechanisms causing outcomes, as developed through the coding of ‘core contents’ abstracts. This is a relatively abstract, conceptual response to aspects of the original review questions. The narrative part of the Findings section concerns only the narrow review focus on issues around timely identification of patients approaching the end of life and the associated initiation of services.

Box 2 **Types of palliative and end-of-life care programmes in primary care and the community in terms of broad programme logic expressed as context–mechanism–outcome (CMO) configurations (brief, see online supplemental appendix 5 for complete box)**

**Type 1 programmes: Programmes seeking to improve the availability of services where systemic and critical gaps exist: in terms of time, location, intensity and responsiveness (when, where, how much and how quick)**

When palliative care is needed in time periods outside of normal working hours and in underserved areas (C), end-of-life care outcomes will improve dramatically and efficiently (O) only if services are time-of-the-day-independent (M), adapted to the location where they are offered (M), flexible (M) and responsive (M).

**Examples of type 1 programmes that address time and timing**

- 24/7 services
- Out of hours (OOH) services
  - OOH specialist palliative and end-of-life care services
  - OOH pharmacy provision of drugs
  - OOH generalist services

- ‘Informal’ OOH services (eg, general practitioner (GP) providing personal phone number)
- ‘Twilight’ services (in the underserved periods between daytime and OOH services)
- Night services
  - Night nursing
  - Night sitting
- Hospice day care services
- Rapid response services

**Examples of type 1 programmes that address location, coverage and proximity**

- Home-based services, including hospice at home
- Hospice outpatient services
- GP practice palliative and end-of-life care clinics
- Community centres services
- Host family respite
- Rural services
- Telecare services
- Deprived areas services
- (Relative) services moving closer to the users

**Examples of type 1 programmes that address intensity and responsiveness**

- Rapid response services
- Improved standard practice (new types of prioritisation)

**Type 2 programmes: Programmes defined through the broad aspect of our humanity and needs being addressed, often as forms of care and support provided by a particular professional or lay group (detail in online supplemental appendix 5)**

As suffering and pain are multimodal (C), we can achieve better quality of life for dying patients (O) when we acknowledge the numerous modalities of experiencing pain and suffering and by acting in (more) holistic ways (M).

**Type 3 programmes: Programmes addressing the management of boundaries and transitions**

As the needs of dying patients at transition points can be extra complex (C) and different services often lack sufficient levels of integration and coordination (C), we can enable each patient to receive the most appropriate and timely care within resource limits (O) if we manage service boundaries and transitions better, in rational yet person-centred ways (M).

**Examples of type 3 programmes that address discharge management**

- Rapid hospital discharge to enable home death
- Discharge roles (eg, discharge community link nurses)
- Discharge letters and templates
- Discharge policies and pathways
- Discharge practices when palliative care needs reduced or prognosis modified

**Examples of type 3 programmes that address referral management**

- Clarification of referral criteria (triggers) and development of documentation
- Rules on referral initiators—who can refer?
- Rules on referral timing—when to refer?
- Referral triggers—what needs to happen so as to refer?
- Referral audits for quality improvement

**Examples of type 3 programmes that address ‘midway solutions’ between service types**

- Intermediate care beds
- Community hospitals
- Hospice at home services
- Primary care doctors with visiting rights to local hospitals

**Examples of type 3 programmes that address the management of transitions and working across settings**

- ‘Alignment models’, for example, aligning the work of GPs and care homes
- Bridging roles—liaison roles, secondments, dual roles
- Case reviews across settings
- Electronic data sharing, Electronic Palliative Care Coordination Systems (EPaCCS)
- Hand-over protocols and forms, particularly for OOH
- Multidisciplinary team meetings
- Service integration work
- Transportation across settings
- Partnerships between ambulance service and other settings

**Type 4 programmes: Programmes prioritising patient-centredness, ownership and empowerment (detail in online supplemental appendix 5)**

As patients and their carers have a range of diverging end-of-life care needs, preferences and wishes (C), we are far more likely to achieve the goals of care that truly matter to them (O) if these are clearly elicited, recorded and acted on (M) and, more broadly, if services are codeveloped with patients and carers (M).

**Type 5 programmes: Programmes addressing different phases of an illness or of the dying process (detail in online supplemental appendix 5)**

As the phase of an illness and/or proximity to death have a profound impact on patients’ treatment and care needs (C), we can improve patient outcomes and support the sustainability of the health service (O) by structuring and delivering services in a phase-centric way, which enables service optimisation, with no relevant needs missed and no unnecessary activities undertaken (M).

**Type 6 programmes: Programmes taking a systemic approach**

As the terminal phase of an illness or the process of dying can be very complex and fast changing and involve a large number of services (C), we are more likely to achieve positive outcomes for the patients, their family and the system (O) and less likely to encounter crisis situations (O) if terminal illness and/or the process of dying is approached in a systematic, proactive and anticipatory manner rather than a piecemeal and reactive one (M).

**Subtypes and examples**

**Programmes aiming to improve identification of patients in need of palliative and/or end-of-life care**

- Development of new prediction and risk stratification tools
- Broader, more systematic implementation of prediction and risk stratification tools
- Improving staff abilities in identifying patients at the end-of-life
- Improving the skills of junior and lower level staff in communicating concerns about patients higher up the hierarchy
- Appropriate recording and communication of such information to other services, for example, through Registers (EPaCCS)

**Programmes enabling discussions of death, dying and care at the end-of-life**

**Programmes aiming to improve advance care planning (ACP)**

- Tools, proformas, templates
- Enhancing basic staff skills in using them
- More in-depth training on using ACP tools, acknowledging challenges such as differences between family and patient preferences, dynamics of preferences, service limitations, creating the right environment for the conversations, etc
- Initiatives to support the broader, more systematic use of such tools

**Programmes aiming to improve integration of care and handling diffusions of responsibility**

- Case management initiatives
- Key worker initiatives
- Data sharing for improved informational continuity
- Palliative care coordination centres
- Bridging roles

**Programmes aiming to develop or refine existing protocols and pathways**

**Programmes based on the use of decision-making tools**

**Programmes aiming to improve monitoring and evaluation systems and processes**

- Enhanced annual reviews of patients
- Patient recall systems and processes
- Palliative and end-of-life care registers and dashboards
- Provision of (comparative) data on palliative and end-of-life care processes and outcomes

**Programmes facilitating internal change through external support**

- Peer facilitation for practices
- Educational facilitation

**Programmes creating a broad supportive environment**

- Financial incentives
- National guidance
- Local change management initiatives
- Identifying and supporting ‘champions’

**‘Meta-programmes’—highly systematic ways of developing new local initiatives and programmes**

**Type 7 programmes: Programmes seeking improvements through staff and volunteer development (detail in online supplemental appendix 5)**

When work environments value palliative and end-of-life care training and development as part of their business-as-usual rather than a matter of short-term projects (C), palliative and end-of-life care provision across the board improves (O) through investment in the knowledge, skills, motivation, attitudes, etc of professionals and lay persons providing care (M) and through creating effective role structures and arrangements (M).

**Subtypes and examples**

**Programmes based on developing new staff roles and forms of task distribution**

- Extended nurse prescribing in palliative care
- Peer facilitators with ‘dual roles’ (e.g. GPs with special interest in PEoLC)
- Bridging roles

**Programmes for staff training and support**

- From palliative care specialists to generalist staff
- Training in specific skills
  - Communication
  - Palliative and end of life care prescribing
  - Advance Care Planning
- Training for specific staff groups
- Training by using different approaches, contexts and platforms (hands-on, online, on-the-job, etc.)
- Support for generalist staff, or even specialist staff, in dealing with rare diseases

**Programmes expanding the roles of volunteers and community members**

- Compassionate cities initiatives
- Volunteers in hospices
- Death dealers

**Type 8 programmes: Programmes defined through the support they provide to informal carers**

The presence of carer-focused programmes in the research literature, as sampled, was not on a par with the presence of programmes of the other seven types. Further research is needed on the degree to which support for informal carers in palliative and end-of-life care translates into programmes which are defined in terms of their carer-focused mechanisms rather than including them as a secondary component.

Note: Here, we offer a typology of palliative and end-of-life care programmes in primary care and the community in terms of the overarching, generic theory (taking the form of a CMO configuration) to which they appear to subscribe. We have abstracted the high-level, generic CMOs from specific CMOs characterising the example programmes analysed, with the examples coming from the 253 ‘core contents’ citations.For instance, on the basis of the brief programme descriptions we have reviewed, we suggest that the abstract mechanism underpinning innovative discharge roles is the management of boundaries between services and settings. Similarly, we suggest that intermediate care beds can be thought of as a boundary management initiative—both between service types (hospital and community) and patient needs (requiring intense professional input and oversight—requiring more limited professional input and oversight). Thus, two programmes which may ‘look’ very different belong, in our classification, to the same type by virtue of their shared theoretical underpinning—shared programme logic of mechanisms causing their outcomes. Further work is required to elicit the theories behind the specific programmes included here and to test our choices of a ‘defining theory’.Online supplemental appendix 5 presents the complete box. More intuitive parts of it have been condensed in this version.

### Timely identification of the end-of-life care stage and timely initiation of services

In the remainder of the Findings section, we argue that current PEoLC policy in England is underpinned by a rough programme theory of Preparation and Planning for Death and Dying whose CMOs can be very powerful, but are also often enough blocked, counteracted, neutralised and even distorted by CMOs arising from the directions of: (1) uncertainties and unknowns in predicting death and dying; (2) orientations towards Living and preserving Hope till the very end; (3) grey zones between palliative and curative care; (4) complexity of decision making about referrals; (5) the loss of pertinent information in rigid hierarchies of knowledge and labour; (6) the ambiguous value of having ‘more time’, which can enable patients and carers to prepare, achieve closure, enjoy each others’ presence for longer, but can also be ‘more of a difficult time’.

#### Preparation and planning for death and dying: the current policy discourse

Timely identification of patients who are likely to be approaching death, often understood as the last year of their lives, opens up precious opportunities to discuss, plan and organise care around a patient’s needs, wishes and preferences, for instance, around preferred place of care or death and the invasiveness of treatments attempted; around the capacities and needs of the patient’s loved ones and/or other informal carers; the features of the patient’s home or other relevant environment; the availability of local services; and with a view to the sustainability of the healthcare system (eg, by seeking to reduce unnecessary admissions and interventions).^27^,^29^

Outside of the immediate context of healthcare, awareness that death may be near enables patients to put their affairs in order; make the best of the time they have got left; complete what has been left undone or find better closure for it; settle and heal relationships; express feelings such as love, regret, forgiveness, gratitude and appreciation; reminisce about their life and find a sense of value and meaning in it; and leave a legacy for future generations.^30 31^

In contrast, delayed identification of the end-of-life stage may mean that patients are robbed of time they believed they had; be denied the opportunity to have a choice in how and where they die; experience significant distress and, ultimately, not have the death they wanted. Families and other people close to the patient may also feel robbed of time and choice; go through avoidable distress and traumatic experiences around the time of death; and be left to deal with feelings such as guilt and complicated grief for years to come.^26 27^

Delays and omissions in identifying dying patients often result from insufficient knowledge, experience, confidence and associated training—one aspect of a much broader problem of knowledge, skills and training in PEoLC.^26 27^ They also reflect deep-seated challenges around information sharing and care coordination.^32^ Disease trajectories also have an impact, potentially irreducible, for example, the trajectories of heart disease or respiratory conditions are less predictable than those of cancer. The transition into the end-of-life stage is also frequently difficult to identify for frail older patients or people with dementia.^26 27^

Detailed CMO configurations and associated evidence can be found in online supplemental appendix 6: table 1, sections 1.1, 1.2 and 1.3.

#### Low accuracy of prognostic judgements in PEoLC

The mainstream Planning and Preparation framework, as summarised above, relies on the assumption that prognosis at the end of life is sufficiently accurate, even if uncertainties are openly acknowledged.^7 26^ Evidence from systematic reviews contradicts this assumption strongly. Out of 20 studies reporting on categorical survival estimates in a systematic review by White *et al*,^33^ only two demonstrate overall accuracy of prognosis over 70% while in 12 studies it is below 50%. In a systematic review on the Surprise Question (‘*Would I be surprised if this patient died in the next 12 months?’*) Downar *et al*^34^ estimate pooled positive predictive value (the proportion of patients who died when the clinician predicted dying) of 37.1% (95% CI 30.2% to 44.6%). No improvements are visible from oldest to newest studies, which could have been expected in view of advances in diagnostic/ prognostic technologies and medical education. No consistent evidence has been found on the impact of professional group, level of experience or time frame of prognosis (eg, imminent death vs within 12 months) on the accuracy of prognosis.^33 35^

Prognostic judgements are made through various combinations of probabilistic objective criteria, clinical judgement and/or subjective intuitions.^36^ A variety of prediction modalities and frameworks are used by health professionals of different professional backgrounds, of different levels of skills, experience and confidence, with different degree of input from other professionals.^33 36^ Prognostic judgements are made of patients in different phases of an illness or frailty.^33^ Judgements about individual patients are also made in complex, dynamic and often overburdened healthcare contexts. Powerful emotional factors also come into play, such as health professionals’ reluctance to share bad news; the value of hope for many patients and their loved ones; or some health professionals’ resistance to ‘admitting failure’ in not being able to do more for a patient.^37^

A targeted search of systematic reviews on prognosis we conducted (key data extracted from the abstracts of 71 reviews) identified a vibrant research field. However, the emerging picture is of significant complexity and distance from clinical applications.

Detailed CMO configurations and evidence associated with prognostic judgements can be found in online supplemental appendix 6: Q5 and Q13; 1.4 and 2.6.1 in table 1; and table 2.

#### Personal cost of inaccurate prognosis

Relative to such findings, the current discourse on timely identification at the end of life, while rightfully eloquent about the consequences of delayed identification, appears surprisingly quiet on the emotional, ethical, pragmatic and other effects of inaccurate predictions of proximity of death (no relevant evidence in our sample). Stakeholders shared anecdotal evidence of exhaustion and emotional turmoil experienced by carers years after a family member had been given ‘weeks’ to live. Survivors pointed out the burden of handling other people’s reactions at a time when they were ‘written off’. The literature on receiving bad news in a medical context and of differing responses to such news may acquire new light relative to findings about significant background uncertainty of prognosis as opposed to the frequent invocation of ‘denial’ in PEoLC.

#### Expanding grey zone between curative and palliative care interacting with patient wishes

The increasing availability of curative therapies, for example, for cancer, and of oral preparations in particular, contributes to later and later referrals to PEoLC, as there is almost always a further line of therapy that can be tried.^38^ Circa 2007, Mintzer and Zagrabbe^38^ identify 26 agents approved by the FDA (Food and Drug Administration) in the preceding decade which have come to be used routinely for the treatment of a variety of malignancies. As of August 2021, the A to Z list of cancer drugs of the National Cancer Institute (USA) lists 641 approved drugs for cancer or conditions related to cancer.^39^

Only a small proportion of patients appear to respond to such therapies and with minimal gains. As a result, many patients die without receiving any, or adequate, palliative care.^38^ Overall, patients may experience a prolongation of suffering rather than life. In view of such outcomes, non-palliative professionals, for example, oncologists, who initiate such courses of action may be judged as overly aggressive in treatments at the end of life^38^; resistant to palliative care; overly committed to a curative ethos and likely to perceive death as a failure; driven by a mindset of having to do something because they are expected to or feel responsible to find a solution; even driven by a hubris that they hold God-like power at the life-and-death line.^37^

Such mechanisms and contexts, however, interact in complex ways with far more patient-driven ones. The outcomes are identical or similar to those explained by health professionals’ resistance to a palliative care ethos and related reasons. The leading driver of decisions to continue with curative treatments are often the wishes of the patient and their family rather than a non-palliative professional’s clinical judgement and recommendation. Following such wishes, rather than aiming to influence the reasoning of patients and carers, is a meaningful choice when new treatment options are available and increasing; when there is always the off-change, the 1% uncertainty, the miracle recovery even; and when hope till the very end is of immense value for some patients and families.^37^ An inclination to respond to patient and carer wishes and preferences for trying once more may, again, be rendered more likely by a non-palliative professional’s own difficult emotions. (*op. cit*.)

CMO configurations and evidence associated with the grey zone between palliative and curative care in online supplemental appendix 6: table 1: sections 2.6 and 2.6.1.

#### Complexity of decisions about referrals to PEoLC services—further factors

##### Family needs

The needs of the family and informal carers may be a far more consequential factor in referring to specialist palliative care services than a health professional’s judgement about a patient’s likelihood to be at the end of their life.^40^ An aspect of this is that some families decline early offers by generalist staff to involve specialist community services (*op. cit*.).

##### System-level factors

A broad range of system-level factors also affect referrals. These may include perceptions of excessive workload/ limited capacity of the specialist community services or lack of confidence in the quality of their work.^40^ Concerns of overspending may be a consideration. The very act of referral is not straightforward either: technical problems may arise with electronic referral systems or the level of detail required may be experienced as off-putting (*op. cit*.). With individual services rarely covering every need of a patient and their family, a particular referral is often only one element of a complete package of care to be activated only if the whole package can be put in place. (*op. cit*.)

##### Potential overconfidence in own abilities

There are some indications in the literature that generalist staff may perceive their PEoLC knowledge and ability to deal optimally with dying patients as better than they actually are, resulting in fewer or later referrals to specialist services than beneficial for patients.^40^ Evidence in our dataset was insufficient to make a strong claim (see online supplemental appendix 6, 4.3.1.1), yet it is possible that brief training events for generalist staff—often part of a strategy for improving PEoLC knowledge and skills—raise confidence in their recipients’ knowledge and skills more than they raise knowledge and skills.

CMO configurations and evidence associated with factors contributing to the complexity of decisions about referrals in online supplemental appendix 6: table 1, sections 2.2, 2.3, 2.3.1, 2.4 and 2.7.

### Reverse engineering of predictions

Temporal criteria in access to PEoLC services have aimed to increase equity, yet referral criteria also serve to manage demand for an enhanced type of service. When a health professional is committed to do one’s best for a patient and/or the family is actively, persistently seeking help, referring professionals may ‘reverse engineer’ the uncertainty of predictions at the end of life^41^ (online supplemental appendix 6: table 1; Table 1, 1.7). They refer a patient to a service by claiming a shorter prognosis than they believe to be accurate. I.e. they ‘play the system’ so as to ensure the best possible care for their patients while the well recognised uncertainty means that they are making no demonstrable error of judgement.

### Hierarchy of knowledge and labour

Staff such as healthcare assistants, care aides, personal support workers who provide hands-on care to patients may make highly accurate observations of less conspicuous changes to the patient’s condition, allowing them to predict a transition towards the end of life. However, their input is often ignored, as they are considered low-level personnel without the training and skills needed for such judgements. Time pressures for senior staff exacerbate the tendency^30^ (online supplemental appendix 6, 1.5).

### Experiences that fill up the time enabled by early identification

Reasoning within the Preparation and Planning framework shows awareness that ‘early identification’ can have its own dangers. It is one of the reasons why ‘timely identification’ is often a preferred term. Still, the leaning of ‘timely’ is in the direction of ‘early’ and the framework does not unpack the potential downsides of early identification.

Early identification of symptom control needs may result in patients being perceived as chronic disease patients rather than approaching the end of life. As a result, they may receive less input from specialist community services, as data in Addington-Hall and Altmann 2000 suggest.^42^ Early referral to services supporting home care may also, in some cases, reduce the likelihood that a patient dies at home.^43 44^ This may be because families and/or professionals find it difficult to sustain care at home for extended periods of time.^43^

A significant time lag between diagnosis and referral to a palliative specialist (in this sense late referral) may, paradoxically at first sight, increase the likelihood that a patient dies at home. Patients may have developed greater acceptance of their terminal prognosis because they have been ‘through more trials, tribulations, and treatment failures, and spent more time in institutions’.^45^ They may thus be more likely to seek, accept and plan for home-based palliative care as opposed to more invasive, hospital-based care with curative or life-prolonging goals (*op.cit*.). From such a perspective, there are individuals for whom trying anything other but palliative care is a precondition for palliative care being seen in a positive light.

CMO onfigurations and evidence associated with the above theme can be found in online supplemental appendix 6: table 1, sections 1.6, 2.9 and 2.10.

## Discussion

### Summary of main findings and comparison with existing literature

This realist review addressed programmes for PEoLC for adults in primary care and community settings, with a further focus on issues around timely identification of patients who are at the end of their lives and the associated timely initiation of services. The key outcomes of the review are:

1. A working typology of PEoLC programmes (and, to a degree, routine services) in primary care and the community, which condenses their immense and often confusing variety. Using a realist logic, we have aimed to centre the typology around ‘deeper’ similarities between programmes (eg, of how and why they are expected to work) rather than more ‘surface’ ones (eg, what visible activities are carried out and where). The typology needs further development and refinement but, we suggest, already enables more reliable comparisons in programme evaluations and commissioning decisions.
2. An argument—an evolving realist theory comprised of interacting CMO configurations—that calls into question a foundational assumption underpinning a broad range of PEoLC policy and services, namely that an accurate enough, be it irreducibly fallible, identification of patients approaching the end of life is typically achievable.

As should be desired of a literature review, this review has both significant overlaps with the existing literature and an original, ‘meta-level’ perspective which makes it a whole—a synthesis—that is greater than the sum of its parts. The crux of our argument around patient identification used ‘ready’ evidence from pre-existing systematic reviews^33^,^35^ which, however, acquired new force once embedded in a broader argument. This broader argument brought to light tensions between accumulated evidence on prognosis, on the one hand, and PEoLC practices and policies predicated on early or timely identification of the dying stage, on the other. Some of the unintended consequences of this policy-evidence mismatch are a cause for concern. The argument also guarded against easy appeals for ‘further research’ and ‘improved tools’. Having consulted over 70 systematic reviews on prognosis from the last 5 years, we suggested that dramatically improved prognostication tools suitable for clinical practice may be a long way away and possibly an unachievable ‘holy grail’.

The CMO configurations we presented as acting against those consistent with the Preparation and Planning framework (under themes such as orientations towards Living and preserving Hope; grey zones between palliative and curative care; complexity of decision making about referrals, etc) are, realistically, familiar to professionals and congruent with the background knowledge of PEoLC researchers. They are also well represented in the research literature, far better than the need to contain this work allowed us to demonstrate. Yet they too received a combined force and meanings they did not possess in isolation. Similarly, the interim focus of the review on ‘time and timing’ constructed a new composite object of inquiry. Time and timing motifs are omnipresent in palliative and end-of-life research and discourse (Box 3). Yet unlike their close counterpart of ‘place’, they have not been turned into an object of study at this general level. Finally, comparisons of our approach and findings with those of a growing number of realist reviews on adult PEoLC, on topics including the district nurse’s role,^46^ music therapy,^47^ meaning of life interventions,^48^ social capital in end-of-life care for patients with dementia^49^ and hospice at home,^50^ suggest that realist synthesis is gaining credibility in PEoLC research but has not yet coalesced into a coherent research programme working towards a shared, cumulative middle-range theory.

Box 3 **Examples of time and timing issues emerging in the reviewed documents**

Timely identification of patients approaching the end of life

Prognosis

Time frame when predictions about dying made (eg, within the last year or days of life)

Timely referral to services

Referrals based on temporal criteria (days, weeks, months to live)

Good timing of/ right time to have ‘the difficult conversations’

Time to prepare for the approaching death

Advance care planning

Temporally defined services (eg, 24/7, rapid response, out of hours, see Box 2)

Waiting lists

Waiting times for services

Respite as ‘time off’

Hours of home care provided

Length of stay in hospital

Duration of use of community services

Time pressures for health professionals

Time for providing palliative care by generalists

More time with a patient as a form of personalised care

Continuity of care

More time with family at the end-of-life

Longevity of services (how long a service has been ‘in business’/funded for)

Timely transfers at the end-of-life

Timing of discharge

The moment of death—sudden, protracted, repeated moments

Being there at the moment of death

Time in managing certification

Importance of how quickly the body is buried for some religious communities

Timely removal of equipment

Timely notification of other services (eg, so that hospital appointment letters are no longer sent).

Box 4 **Implications for practice—initial recommendations and reflections**

The following recommendations and reflections seek to translate the review findings into initial responses to the question of ‘what to do then?’. Solid proposals for changing non-evidence-based practices around patient identification and initiation of palliative and end-of-life care services require further consideration and assessments for acceptability, feasibility, impact and unintended consequences.

1. **Consider the implications of removing, or at least softening, time-based criteria from the sets of referral criteria for palliative andend-of-lifecare services. Current evidence is quite clear that, on average, they are fallible while also associated with important unintended consequences.**
2. **Routine data can be used to conduct local, even clinician-specific, evaluations of overall accuracy of prognosis if the evidence presented here is considered insufficient. Such evaluations can use, for instance, Electronic Palliative Care Coordination Systemsand the dates of addingpatientsto them (when identified asend-of-life) relative to the dates of‘deducting’them (after death). Similarly, the date of first prescribing‘just in case’medications can be linked to the date of death of apatient.**
3. **Increase and diversify the triggers which set in motion the following processes, often associated with preparing for death or the provision of palliative and end-of-life care:**

- Data sharing preferences.
- Assessments for psychological, social and spiritual support needs in the face of experiences of deep vulnerability (of which mortality is a key aspect, but not the only one).
- Elicitation of preferences for home care and home care support needs.
- Carer needs assessments.
- Discussions of preferences for care in situations in the future when mental capacity may be lacking.
- Discussions of ceilings of care.
- Detailed assessments of pain relief and symptom control needs.
  Addressing such needs should not be triggered primarily in association with proximity to death. Nor it needs to be done necessarily, or even preferably, in the context of palliative and end-of-life care services. Skilled health professionals both within and outside of palliative and end-of-life care already attend to such needs regardless of a patient’s prognosis and the context of care, but may be impeded by system fragmentation, policing of boundaries between specialties and demand management criteria, among others.
  At a conceptual level, the definition of palliative care is also broadening towards ‘serious health related suffering’ and away from life-limiting illness, death and dying.^51^ The flipside of this is that serious health related suffering is not the sole provenance of palliative care. There is much in palliative and end-of-life care that is ‘just good care’.

1. **Explore (further) one’s personal standing relative to conversations on time-limited prognosis, death and dying in light of the tension that, on the one hand, current evidence suggests that the accuracy of prognosis is often limited and, on the other, immense negative consequences can arise if a death is completely unexpected till the very end.**
  There is no easy answer to the question of how we acknowledge the prospect of death and dying as a very real, very proximal experience and, as a result, benefit from preparing for it, while also preserving hope for a longer life, if valued. The dilemmas are exacerbated by the tension that health professionals know far more about the signs of irreversible deterioration and death than most patients, yet have far less of the certainty that patients may want, expect and even demand. Positioning oneself relative to such dilemmas has an irreducible personal component. No level of training and professional guidance can fully substitute for it.
2. **The overall unsatisfactory accuracy of prognostic judgements should not become an excuse for avoiding difficult conversations about death and dying—with others and oneself.**
3. **There will be significant economic consequences to removing temporal criteria in referral to specialist or enhanced palliative andend-of-lifecare services. These may be difficult to quantify and exceptionally difficult to meet, yet we cannot be avoiding them by pretending temporal criteria are sufficiently objective, neutral and fair.**

### Strengths and limitations

The originality and potential practical importance of the study outcomes was enabled by a robust yet adaptive process, emphasising rigorous transitions in narrowing the study focus and honing further the tools of realist synthesis. Some of the steps we took (eg, the use of ‘bridging terms’; the mapping of the field on the basis of abstracts coding; the extra systematic approach to focusing the review) were not part of the conventions of the realist review approach but, we believe, are consistent with it and may contribute to its methodological toolkit.

A significant weakness of the review is that the study-final theory around timely identification is, while validated by professionals and subject specialists, tested against a sufficiently broad range of evidence only in the aspect of prognosis. Most of its other aspects are at the level of theory development based on indicative evidence rather than at the level of extensively tested CMOs. Furthermore, our explorations into candidate ‘substantive’/ ‘formal’ theories (briefly, the theories developed within scholarly disciplines to explain specific phenomena in their field of study), whose effective use is seen as a marker of quality in a realist review,^17^ did not bear fruit. Next, the process of constructing the review focus may have been too complex for a relatively small study. The quest for the perfect focus may have led, paradoxically, to suboptimal focus. Some of the steps of this process, for example, the use of abstracts, were also contentious. Finally, a realist review is significantly different from a mainstream systematic review approach and some of its particularities can be perceived as methodologically substandard. Online supplemental appendix 1 represents some of the decisions taken in this review through the critical lens of a mainstream systematic review and aims to bridge the gap.

### Implications for practice

Crucially, we argue that the presence of powerful CMOs which are oppositional to the framework of Preparation and Planning for Death and Dying is not a reason to disinvest—ideologically, emotionally, financially, etc—from it, but to complement and balance it in creative ways, even if the approach taken may appear as backtracking on important advances in open discussions of death and dying. We may be able to achieve better end-of-life care, faster, if we enable specialties, settings and health professionals committed to a life-saving, curative ethos to integrate more of our processes, structures and skills without making them about the end of life. See Box 4 for initial recommendations and reflections.

### Implications for research

The explanatory framework we are proposing will be tested most robustly and expanded most fruitfully by research synthesis studies (realist or other) on: the emotional, ethical, pragmatic and other effects of inaccurate predictions of proximity of death; the reception of ‘bad news’ relative to findings about significant background uncertainty of prognosis; the effectiveness of second-line and third-line therapies; and the relationship between early referral to services supporting home care, on the one hand, and home death, on the other.

## Conclusion

PEoLC programmes in primary care and community settings are here to stay and grow. A realist perspective centred around the concepts of CMO offers a promising way of understanding and improving their workings. Once again, however—after decades of seeking to expand palliative care outside of the realm of cancer—we may need a significant rethinking of the reach of PEoLC programmes. A significant pool of evidence on prognosis suggests that current end-of-life care policy in England and any other country which relies strongly on ‘timely identification’ is nothing short of hubristic in its expectations of working out Death’s timings. And while the day may come, for better or worse, when we are able to perfect the latter, for now we will achieve more if we focus on other ways of improving the end-of-life care we provide, no matter how brief or how long we are providing it for.

## supplementary material

10.1136/bmjspcare-2021-003066 online supplemental appendix 1

10.1136/bmjspcare-2021-003066 online supplemental appendix 2

10.1136/bmjspcare-2021-003066 online supplemental appendix 3

10.1136/bmjspcare-2021-003066 online supplemental appendix 4

10.1136/bmjspcare-2021-003066 online supplemental appendix 5

10.1136/bmjspcare-2021-003066 online supplemental appendix 6
