# Supplementary material for: Timely community palliative and end-of-life care: a realist synthesis
Source: BMJ Support Palliat Care. 2021 Dec 9;14(e3):e003066. doi: 10.1136/bmjspcare-2021-003066 (PMC11671952; doi:10.1136/bmjspcare-2021-003066)
Supplement: online supplemental appendix 1 [file bmjspcare-14-e3-s001.pdf]

Appendix 1: Further detail on realism and realist reviews

Box 1: More on realism

1a. Realism, in the intellectual tradition of Pawson and Tilley, is characterised by the following assumptions*:

1) **Both the material and social words are "real"**; anything that can have real effects is real – crucially, this claim is intended to take a stance on the traditionally controversial **reality of constructs and entities such as culture, gender, social class, programmes, policies, social institutions, ideas**, etc.
2) **Mind-independent and interdependent reality** – both the natural and social world exist and exert their powers **independently of our understanding, judgements and interpretations of them, but they are also affected by** human actions based on the latter (in this sense, have a reality interdependent with our ideas).
3) All enquiry and observations are "filtered through" the human mind; therefore, there is **no "final" truth or knowledge**. However, **we can still work towards a closer understanding of reality** because reality constraints our interpretations and we can continuously test, judge and refine them.
4) All social systems are **open systems** – social systems interact and influence one another and change over time, producing outcomes with or without the introduction of a policy or programme. A realist study **can only show that a policy or a programme *contributed* to an outcome.**
5) **Realism subscribes to a "generative" view of causality** (Bhaskar, 1975, Pawson, 2008) in the sense that, briefly, observable outcomes are generated by largely non-observable causal processes and forces (mechanisms), which operate or not depending on the contexts in which they occur. As a result, outcomes vary across different contexts. Realist research aims to identify the mechanisms which cause outcomes, not just associations between programmes and outcomes.
6) Whether mechanisms generate outcomes depends on the context in which they function – realist research identifies **what it is about the context that determines whether, and which, mechanisms "fire".**
7) **Mechanisms operating in different contexts generate different outcomes** – programmes thus generate different outcomes for different groups of people in different contexts, with **realist research aiming to identify and explain different outcome patterns.**

Key thinkers who have informed the above philosophy are: R Bhaskar (A Realist Theory of Science, 1978), M Archer (Realist Social Theory, 1995), J Elster (Explaining Social Behaviour, 2007), R Merton (On Theoretical Sociology: Five essays old and new, 1967), K Popper (The Logic of Scientific Discovery, 1992), DT Campbell (Methodology and Epistemology for Social Science, 1988), and P Rossi (The Iron Law of Evaluation and Other Metallic Rules, 1987).

* Descriptions of assumptions based primarily on: The RAMESES II Project. Philosophies and Evaluation Design. 2017. http://www.ramesesproject.org/media/RAMESES_II_Philosophies_and_evaluation_design.pdf, with references to "evaluation" changed to "research". Point 5 (on causality) also based on: The RAMESES II Project. Realist evaluation, realist synthesis, realist research – what's in a name? 2017. http://www.ramesesproject.org/media/RAMESES_II_RE_RS_RR_whats_in_a_name.pdf. Paragraph on key thinkers draws on: Pawson R. *The Science of Evaluation: A Realist Manifesto*. London, etc.: SAGE 2013.

Box 1: More on realism, continued

1b. Other "realisms" and other realist philosophies of science

While versions of the positions described in 1a. are standard in the realist research community circumscribed as above, such an interpretation of realism becomes problematic, or at least too facile, when mapped against debates on realism (including distinctions between realism about the world and realism in the philosophy of science), causality, mechanisms, explanation, theories, etc. in "hard-core" philosophy communities, especially if these are part of the philosophy establishment as opposed to the social sciences one.

From the perspective of a broader understanding of philosophical realism, realist research in the Pawson, Tilley and RAMESES tradition has appropriated a name belonging to a much more varied set of conceptualisations and endeavours. The specificity of its understanding of concepts such as causation, mechanisms, theory and explanation, amongst others, while necessary and expected, can also cross into the parochial, contributing to a level of "realist group think" which outsiders to the intellectual community or even critical insiders may, at times, experience.

It is, however, an open question whether a methodological debate which is better embedded in a broader understanding of philosophical realism and/or more varied (realist) perspectives from the philosophy of science around causation, mechanisms, theories, etc. will change substantively, or even at all, the way the applied work is conducted.

1c. Realist synthesis in the context of other "alternative" synthesis methods

Realist reviews are one of a significant number of approaches developed in the 1990s / early 2000s in response to limitations of the mainstream, Cochrane type, systematic reviews.[22] These approaches seek to open up space for meaning-making, explanation, interpretation and theory; a broader understanding of evidence; a greater procedural flexibility; and opportunities for handling significant complexity in an endeavour that has become too self-limiting, largely by virtue of its narrow focus on evidence (or a focus on evidence, narrowly understood).

Basic bibliometric searches in PubMed covering the last five years suggest that out of 29 methodological alternatives to the mainstream systematic review and/or a narrative summary/ synthesis,[22] realist synthesis is amongst the four most widely used methods, alongside thematic synthesis, content synthesis and qualitative meta-synthesis (372 papers retrieved by "realist review" OR "realist synthesis", Sep 2020).

Main advantages of the realist approach we perceived relative to pertinent alternatives were:
1) its focus on programmes as a key unit of analysis (with many interventions in palliative and end of life care in primary care and the community taking such a form);
2) the disciplined, "obligatory" way in which contextual influences are traced, through every CMO-configuration formulated (with outcome patterns in palliative and end of life care characterised by dramatic variations across contexts);
3) its openness to an exceptionally broad variety of evidence (accommodating the endless variety of perspectives that can be taken towards death and dying outside of palliative and end of life care);
4) its commitment to broad stakeholder involvement, including policy makers, practitioners, programme participants and the public, which acknowledges the value of each perspective, the privileged status of some perspectives relative to some questions but, at the same time, does not ascribe ultimate authority to any stakeholder group.

Table 1: Some strengths and weaknesses of a realist review from the perspective of an exemplar (Cochrane-type) systematic review

This table outlines key differences between a realist review and an exemplar (Cochrane-type) systematic review in terms of the research question, inclusion and exclusion criteria, and approach to literature searching. It is not an exhaustive comparison of the methods but, rather, focuses on challenges raised by initial reviewers of this paper. The last column summarises the steps we have taken to "bridge the gap", i.e. improve on features of the realist review which are likely to be perceived as limitations or even failures in comparison to a mainstream systematic review. The steps taken are relatively unusual in the realist synthesis literature but, we argue, consistent with the spirit and methodological injunctions of the approach.

Key element of a review	The realist approach	Strengths relative to the traditional (Cochrane-type) systematic review	Weaknesses from the perspective of the traditional systematic review	Strategy used in this review to bridge the gap
**Research question/ review focus**	The initial research question can be quite broad, as it is typically generated by real-life practical concerns and policy needs as opposed to arising in a research context. It also tends to concern complex social programmes rather than discrete treatments such as new drugs.<br><br>The review focus tends to become narrower as the study	The review questions asked are closer to the actual questions asked in complex policy and practice contexts.<br><br>The work on focusing the review is highly responsive to new cues in the data, which could not have been anticipated at the start of the study.	The initial review questions may appear unspecific, possibly challenged as unanswerable in a scientific way.<br><br>The process of focusing the review may appear to be changing what the review set out to achieve and, as such, to be compromising the integrity of the study.	We used a robust stepwise approach, involving 12 key stages,[2] of making the transitions from the original to the final review questions.<br><br>We still provided a high-level answer to some of the original review questions through the working typology of programmes in palliative and end of life care in primary care and the community.

[2] The process of focusing the review involved the following stages described in the paper: 1. developing a realist typology of programmes for palliative and end of life care; 2. identifying a key theme within the typology which met five criteria (see paper for criteria). The *time and timing* theme was decided upon as a tentative focus; 3. test coding for CMO configurations on time and timing; 4. extensive team discussion. The other main candidate theme for a narrower review focus was "carers". The focus on *identification* within the time and timing theme was decided upon through the following steps: 6. We identified themes related to time and timing in palliative and end of life care using all papers in the main dataset; 7. coded influential UK policy documents on palliative and end of life care for themes related to time and timing; 8. identified the intersections between themes in the research literature and themes in the policy documents (four key themes); 9. developed CMO configurations for all four themes; 10. conducted targeted searches on two of the themes and developed further CMO configurations based on them; 11. consulted stakeholders (the professionals' Advisory Group and the PPI Group) on the emergent findings. 12. finalised the review focus on *timely identification of the dying phase and timely initiation of palliative and end of life care services*.

Key element of a review	The realist approach	Strengths relative to the traditional (Cochrane-type) systematic review	Weaknesses from the perspective of the traditional systematic review	Strategy used in this review to bridge the gap
**Research question/ review focus, cont.**	progresses and initial programme theories begin to be tested against the evidence retrieved (as opposed to being strictly set at protocol stage).[1]<br><br>The review focus is decided upon iteratively, taking into account emerging explanatory possibilities and responding to feasibility demands as explanatory detail grows.		The final review question may bear little resemblance to the original review question.<br>The decision-making process on focusing the review may appear haphazard or "messy".	
**Inclusion/ exclusion criteria**	Studies or parts of studies are included in the review depending on the degree to which they illuminate an emerging theory.	The strong explanatory aspect of a realist review, systematically supported by data, allows programme developers, practitioners and policy makers to make sense of patterns they know from experience and discern new ones. In turn, they can devise or refine programmes on the basis of "theories" (theories of change, logic models, lines of argumentation, etc.) as opposed to being confronted by piecemeal evidence which rarely speaks for itself.	Inclusion/ exclusion criteria can appear vague.<br><br>Explanations may be brought in from other areas creating a potential sense of confusion about the scope of the study.<br><br>As typical inclusion/exclusion criteria such as health condition, setting, age group, etc. correspond to factors which may or may not play a role in theoretical explanations, fundamental features of the review, such as its target	Provided a detailed analysis table (Appendix 5) to demonstrate the logic of the derivations.<br><br>Provided conceptual detail and numerical data on the 9 "levels of relevance" into which we classified studies (Appendix 4 on Literature searching).

[1] See section 3 (Focusing the review) in: The RAMESES Project. Quality Standards for Realist Synthesis (for researchers and peer-reviewers), 2014. https://www.ramesesproject.org/media/RS_qual_standards_researchers.pdf (Accessed Jan 2021).

Key element of a review	The realist approach	Strengths relative to the traditional (Cochrane-type) systematic review	Weaknesses from the perspective of the traditional systematic review	Strategy used in this review to bridge the gap
**Inclusion/ exclusion criteria, cont.**			population, setting, even overall topic, may seem to shift.<br><br>Searches that deliberately (with good justification) reach outside the programme under study (and thus create a worse version of the limitations above) are considered a feature of an excellent review within the realist approach.[3]	
**Search strategy**	Aims to identify sources rich in explanatory detail and evidence that can confirm or reject emerging hypotheses rather than the totality of relevant evidence.<br><br>Develops iteratively, with targeted searches decided upon as the emerging theory is fleshed out.	Responsive to growing understanding of the topic of interest.	As the explanations at the lower levels may include concepts different to those in the review questions, the searches may appear insufficiently matched to the review questions.	Transparency about the search strategy and indication of the topics of all targeted searches, even if, ultimately, not used (Appendix 4 on Literature searching).

[3] See section 5 (Developing a search strategy) in: The RAMESES Project. Quality Standards for Realist Synthesis (for researchers and peer-reviewers), 2014. https://www.ramesesproject.org/media/RS_qual_standards_researchers.pdf (Accessed Jan 2021).
