# Supplementary material for: Timely community palliative and end-of-life care: a realist synthesis
Source: BMJ Support Palliat Care. 2021 Dec 9;14(e3):e003066. doi: 10.1136/bmjspcare-2021-003066 (PMC11671952; doi:10.1136/bmjspcare-2021-003066)
Supplement: online supplemental appendix 2 [file bmjspcare-14-e3-s002.pdf]

Appendix 2: Diagrammatic representation of rough programme theories, as developed at the outset of the study
Accompanies: Petrova, Wong, Kuhn, Wellwood and Barclay, "Dying and time"

Programmes, initiatives, tools, activities …

EoLC patient identification approaches and tools

- "Surprise question"
- Structured prediction tools (examples?)
- GSF register

Approaches and tools for eliciting and recording care preferences (in the context of sensitive conversations) and developing a care plan around them

- GSF
- PPC (Preferred Priorities for Care)
- EPaCCS (Electronic Palliative Care Coordination Systems)
- service-specific care planning tools
- legalistic approaches and tools (e.g. ADRT, power of attorney)

Training

- In having sensitive/ difficult conversations
- Recognising signs of dying
- Using the structured tools

Incentives

- E.g. QOF for GSF register, CQUINS, 1% and 2% LES and DES

High quality EoLC starts with

- **Timely identification of EoLC patients**
- **Elicitation and recording of their care preferences**
- **The preparation of a care plan on the basis of those preferences**

Underpinning assumptions/ mechanisms:

As ~ 70% of deaths are predictable, timely identification is possible for the majority of patients. It needs to be enabled by the appropriate and consistent provision of awareness raising activities, training and tools.

High quality end of life care meets a patient's individual preferences. These need to be identified through sensitive conversations, and adequately recorded and updated.

Lack of skills and confidence amongst health professionals in discussing death and dying is a main barrier. Improving training provision is key.

Progress with the identification and management of cancer patients has been significant. We need to focus on other conditions and also address other sources of inequality.

Supportive context

Greater overall investment in and attention to end of life care

Local and national incentives for identifying EoLC patients, discussing their care preferences and preparing care plans

More open social conversation on death and dying

Appendix 2: Diagrammatic representation of rough programme theories, as developed at the outset of the study
Accompanies: Petrova, Wong, Kuhn, Wellwood and Barclay, “Dying and time”

Countervailing mechanisms

Prognosis is not that straightforward

- The disease trajectory for conditions other than cancer is far less predictable
- Research evidence suggests that predictions in EoLC are frequently inaccurate (e.g. White et al. 2016 systematic review)

Sharing a negative prognosis is not value-neutral – the value of maintaining hope may clash with values around truth (which is uncertainly anyway), preparation, freedom of choice and control over one’s life (and death)

Preferences are not fixed – patient preferences are not necessarily well shaped, can be dynamic, change on the basis of services available, etc.

Elicitation of preferences is complex
Having sensitive EoLC conversations cannot be taught in a 2-hour training session

This is not a conversation that fits in a 10-min appointment

Trust and the quality of the relationship with a patient are important. You need to have/ build the right relationship to have the conversation.

There is much noise in communication channels in principle, let alone in the case of conversations about death and dying, where euphemisms are often used (i.e. what clinicians have said and what patients have heard can be quite different)

Care planning
Ultimately, you are trying to predict something that is highly unpredictable.

If the services are not available, what you have planned for is totally irrelevant. It is even unethical as it may sound like a promise/ commitment when discussed with patients.

The identification of EoLC patients, the elicitation and recording of their preferences and the preparation of a care plan are more complex than may appear at first sight

Challenging contexts

Service level

If the services are not available, their availability unpredictable and we are going to do what we are going to do anyway, it is problematic, even unethical to be eliciting preferences.

Patient social context

Patient and his/ her family preferences may differ, and family members are also our patients. We need to consider them too in our planning.

Informational context

Data sharing only happens in pockets, not at all, or using traditional methods of inter-professional communication. We may be identifying and recording preferences and plans in our service, but if colleagues are not aware, much of that effort is wasted.

Appendix 2: Diagrammatic representation of rough programme theories, as developed at the outset of the study
Accompanies: Petrova, Wong, Kuhn, Wellwood and Barclay, "Dying and time"

Programmes, initiatives, services, etc.
(in addition to standard practice, e.g. GP and DN visits, care provided by the family)

Home-based services:
- Community palliative care teams
- Hospice@home services
- Rapid response teams
- Paid carers
- Lay volunteers

Provision of equipment for home care

Admission avoidance/ hospital stay reduction schemes:
- Better information provision to ambulance staff and A&E through data sharing initiatives
- Fast track discharge
- Palliative care teams which work across the hospital and community
- Training

Support for carers
- Respite
- Training
- Counselling, confidential telephone lines, carer groups, etc.

As most patients prefer to be cared for/ die at home, community care is both congruent with patient preferences and more financially sustainable

Underlying assumptions/ mechanisms

Home is the place where people feel most at peace, supported, safe and where they will be closest, in their final hours, to the ones who love them.

Community care is cheaper than hospital care.

Busy, open and impersonal hospital spaces can be a traumatic environment to die in and/ or witness your loved one die.

If a patient prefers to be cared for at home (and this information is known and shared appropriately) and if we provide sufficient support at home, hospital admissions at the end of life are, generally, both unwanted and unnecessary.

There is a growing range of high quality community EoLC services. However, we need to address gaps in provision and sources of inequality.

Supportive context

Greater investment in community services in general and community EoLC services in particular

Improvements in the local IT infrastructure (e.g. mobile working, data sharing)

Compassionate communities

Appendix 2: Diagrammatic representation of rough programme theories, as developed at the outset of the study
Accompanies: Petrova, Wong, Kuhn, Wellwood and Barclay, "Dying and time"

Countervailing mechanisms

There is a level of idealisation of home as a place of care/ death – e.g. help is not available at the press of a button and pain may be poorly controlled as services are not sufficient; the equipment turns the home into a hospital at home, etc.

Admissions may only appear inappropriate, even if a patient's core preference is to be cared for/ die at home and even if those preferences are known – e.g. patients and family get scared; carers are exhausted; community services to keep the patient at home are not available at the time they are needed and it is safest to take the patient to hospital

Cost savings do not mean no investment

Good community services cost money, and this often needs to be invested in advance.

There is limited evidence on the overall costs of community services.

Family time is often unaccounted for, and the costs which are thus shifted to families can be significant.

Providing services which meet the complex and dynamic patient preferences and the highest standards of EoLC in the community is fraught with difficulty. The financial advantages of community care are not as definitive as they may seem.

Challenging contexts

Service level

The services which are part of standard practice (e.g. GP and DN involvement) are under so much pressure that an important pillar of community services is not as reliable as assumed.

The provision of EoLC community services is unequal.

Financial context

Funding cuts

Funding arrangements can lead to perverse incentives or disincentives to community care.

Geography

Remote, rural and disadvantaged areas will typically have less community services in place.

Areas which fall at administrative/ team coverage boundaries can fall between two chairs.

Social context

Community EoLC services rely on some level of support from a person's social network. Not everybody has that.
