# Supplementary material for: Timely community palliative and end-of-life care: a realist synthesis
Source: BMJ Support Palliat Care. 2021 Dec 9;14(e3):e003066. doi: 10.1136/bmjspcare-2021-003066 (PMC11671952; doi:10.1136/bmjspcare-2021-003066)
Supplement: online supplemental appendix 3 [file bmjspcare-14-e3-s003.pdf]

Appendix 3: Stakeholder involvement

Here we report on the stakeholder involvement in our review using the Short Form of GRIPP2 (Guidance for Reporting Involvement of Patients and the Public) reporting checklist, while adding the *Definition* and *Theoretical underpinnings* parameters from the Long Form. Clarification of terminology and background assumptions was needed, as stakeholder involvement in realist research is broader than Patient and Public Involvement (PPI).

GRIPP2 forms as in:

> Staniszewska S, Brett J, Simera I et al. GRIPP2 reporting checklists: tools to improve reporting of patient and public involvement in research. *BMJ* 2017;358:j3453, http://dx.doi.org/10.1136/bmj.j3453

1. Definition

While we did not start from a formal definition of stakeholder involvement, we had extensive team discussions of how to proceed with it and how to optimise it, particularly relative to standard challenges faced by PPI, such as professionalisation or unrepresentativeness of PPI groups. Below is a post-factum articulation of our starting positions:

Stakeholder involvement is a process whereby non-researchers and non-study participants are consulted with the goal of improving the study relevance; focus; applicability; chances of successful recruitment and retention of participants (or, in a review, the identification of relevant sources); valid analysis; and effective dissemination of findings. It is also a form of ensuring greater accountability and broader social feedback for research, which can become too disconnected from its intended users and beneficiaries and the society which often funds it.

More broadly, from the perspective of social inclusion and justice, the patient and public involvement aspect of stakeholder involvement is one of numerous ways in which individuals and social groups which are not in powerful positions can be encouraged to influence social conversations and practices which affect them; develop/refine the knowledge and skills to enable them to do so successfully; and develop the confidence that their views are valuable and that there are ways for them to be heard.

At the start of the work, our main concerns about PPI involvement were:

1) the tendency for PPI groups to be insufficiently representative of the breadth and substrata within 'patients and the public' and to reproduce entrenched social inequalities (e.g. PPI members are often white, middle class, educated, articulate, retired);

2) the tendency for PPI group members to become professionalised (and, arguably, lose some of the novice's and outsider's ability to notice and challenge problems and inefficiencies);

3) the degree to which PPI is being advanced as a requirement and unquestionable good in health research (provided it is "done well", and not in a "tokenistic" way), limiting researchers' abilities to engage probingly and critically with it without being accused of elitism and paternalism. While being strong supporters of PPI, we do believe the practice needs a far more open and critical debate to which we are aiming to contribute.

In responding to reporting requirements throughout the study (including in using the GRIPP checklists here, which address PPI involvement), we had to manage an ambiguity characteristic of realist methods and other methods relying on broad stakeholder engagement and involvement. "Stakeholders" in such contexts includes not only patients and the public, but also professionals, policy makers, commissioners, etc. Moreover, no type of stakeholder in a realist study is, by default, privileged or more important than any other type of stakeholder. Different types of stakeholders are likely to have experience with, be sensitive to and 'see' different aspects of the big picture needed for the study.

Furthermore, as the involvement of professional stakeholders in research is typically structured around the needs of the study and the culture of research as opposed to the practices, rules, norms, etc. constituting a professional role (e.g. a health professional is a professional but outside of their field of practice), a professional stakeholder can also provide input 'simply' as a patient and member of the public.

It is an interesting and, we believe, open question if patients and the public should be the 'privileged' stakeholders (as having a reporting checklist only for this stakeholder group seems to imply). The question is particularly relevant in the case of research whose recommendations can be acted on first and foremost by health professionals, policy makers, commissioners, service developers, etc., even if its ultimate beneficiaries are intended to be patients and the broader society.

2. Theoretical underpinnings

Our literature review was conducted within a realist framework, as described in sources such as Pawson and Tilley (1997), Pawson (2002 a,b), Wong et al. (2013) and The RAMESES Project (2014). In realist studies, stakeholders with knowledge of a programme (intervention) are involved in ways that enable a researcher to put to test, and as a result confirm, falsify and refine, a theory about the programme's workings. Expertise (and inexpertise) concerning a programme theory varies from aspect to aspect of a problem and from one programme to another (Pawson and Tilley, 1997; p.159-160).

Tentatively, it can be expected that programme subjects (e.g. patients in a healthcare context) will be more sensitized to the mechanisms in operation within a programme rather than to its contextual

constraints and outcome patterns (*op. cit.*, p. 160). As mechanisms, within a realist framework, provide reasons and resources encouraging participants to change, patients are well positioned to comment whether this has been effective, albeit most likely presenting a rather personal view of the interaction between programme mechanisms and their (the patient's) pre-existing capacities (*ibid*.). Practitioners, in turn, are likely to have specific ideas on the aspects of the programme that make it work; likely to have experienced both successes and failures of it; and to have some awareness of people and places for whom and in which the programme works (*op. cit*., p. 161). Typically, they have a limited ability to systematise, abstract and generalise from their personal and institutional experience (*ibid*.).

Evaluators (researchers) are bringing in the theoretical knowledge – CMO-configurations they are familiar with from other programmes and more abstract propositions from social science theories. They are, however, stronger on form than content (*op. cit.*, p. 161).

Each of these stakeholders has something to teach the others and something to learn from them (*op. cit.*, p. 161).

Describing the role of stakeholders in realist studies, as per core methodological texts, is a research question in its own right. In our still evolving understanding, the realist approach does not ascribe a privileged status to patients and the public in comparison to other stakeholders in the research process. In contrast, mainstream PPI thinking, one of whose checklists we are using, appears to adopt such a view.

3. Aims

Relative to the theoretical underpinnings described above, stakeholders are involved in a realist study so as to contribute, with their experience and perspective, to the testing and refinement of a programme theory, which is the study's main focus and outcome.

4. Methods

4.1. Convening stakeholder groups

Within the core study team, we had several (4-5) extensive discussions about stakeholder involvement and the most effective ways of enabling it.

The Cambridge Palliative and End of Life Care (CPEOLC) Group, which hosted the study, has a long-standing tradition of PPI involvement. However, as key concerns raised in the team discussions had been the downsides of PPI professionalisation and the value of broadening the variety of perspectives we had access to, we decided against approaching the PPI network of the CPEOLC group.

Initially, we engaged with an existing Sustainability and Transformation Partnership (STP) End of Life Care Board, which also has PPI representation, as a form of Advisory Group for the review. This was because all key local (Cambridgeshire & Peterborough) stakeholders in palliative and end of life care

were already represented there. The original meeting provided us with some useful insights, but we decided to convene a project-specific Advisory Group.

We further approached a pre-existing PPI group, part of an infrastructure established and maintained by the National Institute for Health Research (NIHR) Cambridge Biomedical Research Centre (BRC). Unlike our CPEOLC network of PPI contributors, PPI members on this panel had no specific prior involvement in end of life care research. We assumed this would allow us to tap into less familiar points of view. 8 panel members attended the first meeting (early stages of the review) and 6 the second (towards the end of the review).

We also convened a project-specific Advisory Group using SB's networks of palliative and end of life care professionals. 16 people expressed interest to join the group, of whom 8 were able to attend the first project meeting and 6 the second meeting. Several group members who could not attend either meeting provided feedback over email.

4.2. Activities

The main mode of engagement with the two stakeholder groups were face-to-face meetings, separate with each group: one meeting with each of the groups in October 2018 and one meeting with each of the groups in November 2019. The main goals of the first set of meetings were to discuss the scope of the review and the emerging rough programme theory. The main goals of the second set of meetings were to receive feedback and solicit reflections on key findings, serving to test further and help us refine the CMO-configurations under development.

Two members of the core research team attended each of the meetings (MP with either SB or IW) with the exception of the second PPI meeting (MP only).

Following the first meeting, we produced a 20-page document that brought together the input of the professionals' Advisory Group and of the PPI Group. The document covered issues discussed at our original meetings and elicited initial Context-Mechanism-Outcome configurations. We circulated it to both groups with a request for feedback on "missing pieces", if any, and "priorities within the priorities". Feedback and additions were minimal, with overall comments that this was an impressive document.

Throughout the project and primarily around meetings with the groups, MP also had email exchanges with stakeholders, enabling further insight into the palliative end of life care-related experiences of patients, carers and health professionals and the workings of current services and programmes.

5. Outcomes of stakeholder involvement

5.1. Impact on the scope of the review

We received important guidance on the scope of the review from both groups. For instance, we were inclined to exclude research on care homes, hospices and day care centres. However, it

became apparent that such settings had been central to both positive and negative experiences of end of life care for members of the PPI Group while accompanying family members in their final days. The hospice representation on the professionals' Advisory Group also meant the prominence of the setting remained high, while, within the team, we had discussed excluding literature on hospices by virtue of them being "too specialist", even if community based.

Both meetings with the PPI Group underscored in emotional ways the importance of carers in palliative and end of life care. The research we had been identifying and screening, the policy documents reviewed, and the input of the professionals' Advisory Group would not have resulted in a similar prominence of the carer perspective. The latter remained a key consideration throughout the data identification, analysis and synthesis stage, both in terms of explanatory models and issues prioritised.

5.2. Contribution to theory development and refinement

The discissions at the inaugural meetings with both groups, and particularly with the professionals' Advisory Group, contributed significantly to developing the rough programme theory and specifying sub-questions within the broad review questions we had formulated for the study protocol. A detailed document on this contribution is available from the authors. Participants' experiences, ideas and reflections from those meetings also supported the formulation of CMO-configurations during data analysis.

5.3. Impact on achieving balance

The second PPI meeting was exceptionally helpful in highlighting, in rather emotional ways at times, that a significant proportion of patients and carers do not receive palliative and end of life care at all. In view of their, at times, excruciating experiences, members of the PPI Panel intensely disagreed with some of our criticisms of a policy overemphasising identification of the end of life stage. The strong reaction against some of our evidence-based claims alerted us to the importance of careful framing and of fairness to the more conventional perspective, which should not be undermined, only expanded.

5.4. Impact on motivation and accountability

We experienced both forms of stakeholder involvement as very motivating in conducting the review. We felt encouraged to proceed with it and more confident in its importance and relevance.

We also had clear reminders that we need to give sufficient priority to the dissemination of findings and target dissemination efforts at a variety of primary audiences.

6. Discussion and conclusions

The involvement of both stakeholder groups was key to the direction of the study, as the specific outcomes above demonstrate.

7. Reflections/critical perspective

7.1. Representativeness and fluctuations of membership

Even though we sought an external PPI group (external to the Cambridge Palliative and End of Life Care research group) which could draw on a much larger pool of potential participants, the group was still insufficiently representative of the broader public.

It was also inconsistent in membership, with three new members and a new coordinator attending the second meeting. As a result, we needed to re-introduce the project and make space for the palliative and end of life care experiences that have brought each of the panel members (and us) to the meeting. Creating the right emotional environment is crucial for PPI involvement in palliative and end of life care research. However, in new groups or groups with significant fluctuations of membership, it may mean that the substantive work moves to the background for the better part of a meeting.

7.2. Expectations

In a literature review, the shape of the study is strongly determined by the available literature. There is a risk that the particularities of the available research, together with the need to focus the review further and further from its initial ambitions, creates, at the end of a study, the impression that the research team have taken very little of the contribution of stakeholders on board.

Our second PPI meeting also made us wonder if PPI groups are not almost exclusively consulted at the beginning of a study, making a meeting focusing on sense checking and refining findings unexpected and, by extension, requiring a much more detailed introduction. We found the meeting in question exceptionally helpful, yet in a diffuse way (see 5.3. *Impact on achieving balance*), almost unrelated to the specifics of our findings (which were discussed in much greater detail with the professionals' Advisory Group). The findings moved to the background, as it felt far more important to open up space to discuss the issues that were coming up in the PPI Group there and then. Perhaps the Panel perceived their role as giving initial input on relevant issues, while the research team (represented by MP) was hoping to obtain feedback on highly specific and already formulated findings. Extra care may be needed in preparing and introducing such meetings, making clear they are "different" to normal PPI meetings.

7.3. Professionalisation – a revised perspective

A key challenge of PPI involvement we discussed at team meetings early on in the study was the level of professionalisation in PPI, including in the PPI Group associated with the Cambridge Palliative and End of Life Care Group – a group we had direct access to but decided not to approach for the purposes of this study. With the benefit of hindsight, we see many more of the advantages of such professionalisation. The iterativity of participation allows for the development of long-standing relationships between researchers and PPI representatives, and amongst the PPI representatives themselves. This, in turn, contributes to a safe, trusting environment which is key in palliative and

end of life care research. Repeated participation also allows for the clarification of roles and expectations and for ongoing, both formal and informal, training in research methods.

We would like to thank sincerely all stakeholders who contributed to this study.

REFERENCES:

Pawson R and Tilley N. Realistic evaluation. London: Sage; 1997.
Pawson R. Evidence-based Policy: In Search of a Method. Evaluation 2002; 8(2): 157-81.
Pawson R. Evidence-based Policy: The Promise of Realist Synthesis. Evaluation 2002; 8(3): 340-58.
Wong G, Westhorp G, Pawson R and Greenhalgh T. Realist Synthesis: RAMESES Training Materials. Jul
2013. http://www.ramesesproject.org/media/Realist_reviews_training_materials.pdf
The RAMESES Project. Quality Standards for Realist Synthesis (for researchers and peer reviewers). 2014. http://www.ramesesproject.org/media/RS_qual_standards_researchers.pdf
