# Supplementary material for: Timely community palliative and end-of-life care: a realist synthesis
Source: BMJ Support Palliat Care. 2021 Dec 9;14(e3):e003066. doi: 10.1136/bmjspcare-2021-003066 (PMC11671952; doi:10.1136/bmjspcare-2021-003066)
Supplement: online supplemental appendix 4 [file bmjspcare-14-e3-s004.pdf]

Appendix 4: Approach to searching and screening the literature

1. Generating the main dataset

The scope, databases and keywords for the literature searches were discussed from the start of the project, in the context of its regular team meetings, by IK (library and information specialist), MP (lead researcher, domain expert – palliative and end of life care), IW (systematic reviews expert), GW (realist methods expert and domain expert – general practice), SB (domain expert – palliative and end of life care and general practice). IK then designed test searches, whose contents and retrieval were reviewed by MP. Minor modifications were introduced. This work generated the main search strategy for the review. It combined four blocks of search terms around:

- palliative and end of life care;
- primary and community care;
- United Kingdom, using the filter for Medline by Ayiku et al.[1] and adapting it for other databases;
- programme (theory, model, philosophy).

Eight databases were searched from 1990 onwards, with the search run on 24th April 2018:

- Medline via OVID
- Embase via OVID
- CINAHL via EbscoHost
- PsycINFO via EbscoHost
- Web of Science
- ASSIA via ProQuest
- Sociological Abstracts via ProQuest
- SCIE Social Care Online

[1] Ayiku L, Levay P, Hudson T, Craven J, Barrett E, Finnegan A, Adams R. The MEDLINE UK filter: development and validation of a geographic search filter to retrieve research about the UK from OVID MEDLINE. *Health Info Libr J*. DOI: 10.1111/hir.12187

The complete search strategy for each of the databases is presented at the end of this document, under **Main search, version 3**.

The main search generated a deduplicated dataset of **3039** citations.

Subsequently, records between 1990 and 1997 were removed after a decision to use the 2008 End of Life Care Strategy for England[2] as a reference point and review publications 10 years before and 10 years after it. This decision aimed to ensure higher current relevance of the publications analysed and explore the impact of the Strategy on programme theories.

This resulted in a main dataset of **2,832** citations.

2. Initial screening of the main dataset – refinement of inclusion-exclusion criteria; levels of relevance and saturation

Relevance of a piece of evidence in the realist approach is determined relative to the programme theory being tested and refined. As the theory itself is under development – from the initial "rough" programme theory to the more refined realist programme theory which should be the outcome of the review – relevance is a dynamic and, at least initially, underspecified concept.

Inclusion and exclusion criteria thus cannot be decisively fixed during early stages of the review process or, if done, the explanatory potential of the review may be constrained. In the case of diverse and complex interventions, as was our case of programmes for palliative and end of life care in primary care and the community, the specification of definitive inclusion and exclusion is even more problematic.

Consequently, while we were guided by the set of inclusion-exclusion criteria outlined in the PROSPERO protocol (summarised in Box 1 below) and the rough programme theory developed within the team and consulted with stakeholders (Appendix 2), we still worked within relatively flexible boundaries of relevance, capable of accommodating the realist approach in the context of a highly complex topic.

---

[2] Department of Health. *End of Life Care Strategy: Promoting high quality care for all adults at the end of life.* Jul 2008. https://assets.publishing.service.gov.uk/government/uploads/system/uploads/attachment_data/file/136431/End_of_life_strategy.pdf (Accessed Sep 2020).

Box 1: Summary of inclusion-exclusion criteria as per PROSPERO protocol

https://www.crd.york.ac.uk/PROSPERO/display_record.php?RecordID=97218

Age: “Adults (over 18 years of age) whose death is perceived as imminent or who have advanced, progressive or incurable conditions. As realist reviews focus on theory development, we may, where relevant, consult literature on other populations, e.g. paediatric patients.”

Interventions/ exposures: “Programmes, interventions, initiatives, approaches, tools, etc. for the provision of palliative and end of life care in primary care and other community settings.”

Definition of end of life care referred to the last 12 months of life. Definition of palliative care (WHO) referred to physical, psychosocial and spiritual suffering. See details in full protocol.

Understanding of primary care and the community: mostly in opposition to hospital care and hospice inpatient care.

Examples of programmes of interest:
- the upskilling of General Practitioners and the support for optimising provision of palliative and end of life care in general practice (i.e. primary care-based programmes);
- care provided by palliative care specialists in the patients' homes or as hospice outpatient services;
- initiatives aimed at improving end of life care in care homes;
- programmes which are developed, funded and staffed fully or primarily by charities and the voluntary sector;
- programmes offered by the private sector, e.g. paid-for care in patients' homes;
- virtual community initiatives.

Main outcomes: 1) "good death", typically as experienced by the family and carers of the deceased; 2) quality of care, as experienced by the patient, their family and carers, and healthcare staff, or as measured by quantitative metrics; 3) cost effectiveness.

Additional outcomes: coordination of care; reduction of unnecessary and unwanted hospital admissions; place of death.

Study design: No restrictions on study design. Non-empirical work also included.

Citations were first screened at the level of title, abstract and keywords by MP. The protocol envisaged that a second reviewer would screen 20% of all citations. Double screening in the context of systematic reviews with a strong interpretative component is typically seen as a way of enriching the analysis as opposed to enabling the checking for and correction of errors. In this review, we considered it a way of enhancing the process of theory development. As the team member who had started the double screening process left the institution, this work was not completed. Relative to the numerous other corrective and enriching processes established for the study (study team meetings; meetings of researchers conducting realist reviews across the Evidence Synthesis Working

Group; meetings with a professionals' Advisory Group and a Patient and Public Involvement Group; individual consultations with GW, the methodological expert on the study, etc.) and the openness to interpretative differences of any conceptual/theoretical review, we decided not to make alternative arrangements for double screening.

As opposed to aiming for a binary include-exclude decision, the process of screening was one of annotating citations for level of perceived relevance. Often, a brief note was added to the EndNote record to explain the decision, especially if it was not immediately clear from the paper title.

Inclusion categories:

- **Include, (potentially) core contents** – these citations were perceived as matching fully, or very closely, the questions of interest. In the majority of cases, they also fitted fully the main inclusion criteria (palliative and end of life care, community, UK, adults).
- **Include** – the generic 'include' category.
- **Include, broad** – for citations that covered topics outside of palliative and end of life care, community settings, adults and/or the UK, but suggested arguments and evidence that were relevant to the rough programme theory and its testing and refinement. Typically, the deviation from exemplary relevance was on 1 or 2 of the main inclusion criteria. If it was on more, the paper was more likely to be annotated as 'maybe include' (see below). That said, no numerical rule was followed around degrees of relevance.

Exclusion categories:

- **Exclude** – the generic 'exclude' category. The majority of directly excluded papers were from the basic sciences and from developing world healthcare systems (whose context we have pre-judged as too different to be sufficiently relevant).
- **Exclude, search precision-relevant** – we noticed that a number of terms (such as "C-terminal", "N-terminal", "carboxyl-terminal", "terminal half-life") reappeared frequently and consistently picked false positives, while never picking true positives. We started annotating such papers as an opportunity to explore strategies for improving the specificity of subsequent searches.
- **Exclude, unrecognised duplicate** – despite having conducted an automatic de-duplication of the dataset, duplicates were still appearing.

- **Exclude, yet broad relevance** – such papers had some relevance to issues of interest, but those issues were typically too broad features of the topic (e.g. overall tendencies in the distribution of staff across the health workforce).
- **Exclude, but preserve for bereavement review** – those papers addressed bereavement in primary care and the community, with the team commencing a realist review on complicated grief around the time of the screening. Under different circumstances, papers from this category would have been distributed across 'exclude' and 'include' categories. A small number of bereavement papers were included in this review too, but only to the extent to which they illuminated care before death.

Uncertain categories

- **Maybe** – these were papers which 1) concerned settings, populations and geographies that fell under our exclusion criteria (e.g. hospital, children, non-UK), but some of the CMO configurations or elements they pointed towards appeared transferable; 2) had title/ abstract/ keywords which were not clear enough for a definitive decision; 3) discussed core ideas within the field of palliative and end of life care which could help elicit fundamental, but potentially too generic, CMO-configurations (as in papers on the history of palliative care); 4) explored highly specific settings and contexts of death and dying (e.g. prisons, suicides) which we were not excluding, but were not expecting to cover in detail either.
- **Include on limited information** – the title, abstract, and keywords suggested 'include', but there was a significant level of uncertainty to the decision due to limited information.
- **Exclude on limited information** – the title, abstract, and keywords did not give sufficient justification to follow up a paper to its full text, but there was a significant level of uncertainty to the decision due to limited information.

After screening 1,226 citations out of the main dataset (of 2,832), a level of saturation was achieved. Reaching saturation at this stage – roughly half of the dataset – was also taken as an opportunity to use half of the dataset for theory development (over and above that of the rough programme theory) and the other half for theory testing. The table below presents the proportions of the different levels of relevance of citations.

LEVEL OF RELEVANCE	N	% of 1226 total
Exclude, standard	250	20.4
Exclude, search precision-relevant	90	7.3
Exclude, remaining duplicates	25	2.0
Exclude, yet broad relevance	59	4.8
Exclude, bereavement review	20	1.6
Maybe or limited information to include or exclude	85	6.9
**Irrelevant or of low perceived relevance**	**529**	**43.1**
Include, core contents	253	20.6
Include, standard	250	20.4
Include, broad	194	15.8
**Relevant or potentially relevant**	**697**	**56.9**

3. Targeted searches

Targeted searches – both simple and refined (the latter developed by IK, the library and information specialist on the project) – were run at various stages in the process of narrowing the review focus.

Main topics included:

- 24/7 specialist palliative care services
- night sitting, night nursing, care at night
- rapid response services
- referrals in palliative and end of life care
- staff shortages (in the NHS generally and palliative care more specifically), with implications for time available for patient care
- impact of performance management approaches (measurements, targets, incentives, etc.) on the work done (how "time is invested")
- family size, structure and dynamics, and health of carers
- systematic reviews on prognosis in palliative and end of life care and/or predictions of survival or death.

Apart from the last topic (search strategy given at the end), all other topics were gradually excluded from the review focus. However, a number of the papers included in the review were found through those supplementary searches.

Main search, version 3

End of life AND Primary Care AND Britain

	A<br>End of life AND Primary Care AND Britain AND model	B<br>(End of life AND Primary Care AND Britain) NOT (End of life AND Primary Care AND Britain AND model)
Medline via OVID	1141	2812
Embase via OVID	1303	3151
CINAHL via EbscoHost	449	1037
PsycINFO via EbscoHost	460	716
Web of Science	527	1051
ASSIA via ProQuest	316	1106
Sociological Abstracts via ProQuest	25	49
SCIE Social Care Online	56	112
Total	4277	10034
Total deduplicated	3039	

1990 onwards

Searches run 24th April 2018

Medline

Ovid MEDLINE(R) Epub Ahead of Print, In-Process & Other Non-Indexed Citations, Ovid MEDLINE(R) Daily and Ovid MEDLINE(R) 1946 to Present

exp terminal care/ or exp palliative care/ or exp palliative medicine/ or exp terminally ill/ or exp hospices/ or ((end adj of adj life) or palliative* or terminal* or hospice*).ti,ab. or (last adj1 (week* or month* or day*)).ti,ab.

AND

(exp primary health care/ or exp community health services/ or exp home care services/ or exp general practice/ or exp family practice/ or exp charities/ or exp volunteers/ or exp organizations non-profit/ or exp community health nursing/ or exp spouses/ or exp home nursing/ or exp home care services/ or exp homes for the aged/) or

((Lay adj (care* or work*)) or volunteer* or (third adj sector) or (non-profit* or nonprofit*) or charit* or spouse* or wife* or wives* or husband* or partner* or (home* adj2 (care or residential* or nursing* or (old adj (folk* or person* or people*)))) or (primary adj (care or healthcare or (health adj care))) or (general adj practi*) or (family adj (practi* or doctor* or physician*)) or (community adj2 (care* or healthcare or nurs* or compassionat*)) or (district* adj nurs*) or (marie adj curie) or (macmillan adj3 (nurs* or service* or support*))).ti,ab.

And

(exp united kingdom/ or (national health service* or nhs).ti,ab,in. or (gb or "g.b." or Britain* or (british* not "british Columbia") or uk or "u.k." or united kingdom* or (england not "new england") or northern Ireland* or northern irish* or scotland* or Scottish* or ((wales or "south wales") not "new south wales") or welsh).ti,ab,jw,in.)

https://onlinelibrary.wiley.com/doi/epdf/10.1111/hir.12187

And

(theor* or model* or concept* or philosoph* or program*).mp.

Embase

Embase 1974 to 2018 April 02

exp *terminal care/ or exp *palliative therapy/ or exp *terminally ill patient/ or exp *hospice/ or ((end adj of adj life) or palliative* or terminal* or hospice*).ti,ab. or (last adj1 (week* or month* or day*)).ti,ab.

AND

(exp *primary medical care/ or exp *community care/ or exp *home care/ or exp *general practice/ or exp social welfare/ or exp non profit organization/ or exp volunteer/ or exp community health nursing/ or exp spouse/ or exp home care/ or exp nursing home/ or exp home for the aged/)

Or

((Lay adj (care* or work*)) or volunteer* or (third adj sector) or (non-profit* or nonprofit*) or charit* or spouse* or wife* or wives* or husband* or partner* or (home* adj2 (care or residential* or nursing* or (old adj (folk* or person* or people*)))) or (primary adj (care or healthcare or (health adj care))) or (general adj practi*) or (family adj (practi* or doctor* or physician*)) or (community adj2

(care* or healthcare or nurs* or compassionat*)) or (district* adj nurs*) or (marie adj curie) or (macmillan adj3 (nurs* or service* or support*))).ti,ab.

And

(exp *united kingdom/ or (national health service* or nhs).ti,ab,in. or (gb or "g.b." or Britain* or (british* not "british Columbia") or uk or "u.k." or united kingdom* or (england not "new england") or northern Ireland* or northern irish* or scotland* or Scottish* or ((wales or "south wales") not "new south wales") or welsh).ti,ab,jx,in.)

And

(theor* or model* or concept* or philosoph* or program*).mp.

Web of Science

TOPIC: (theor* or model* or concept* or philosoph* or program*)

Indexes=SCI-EXPANDED, SSCI, A&HCI, CPCI-S, CPCI-SSH, BKCI-S, BKCI-SSH, ESCI, CCR-EXPANDED, IC Timespan=All years

TOPIC: (united kingdom or uk or gb or great britain or england or scotland or northern ireland or wales or welsh or scottish or northern irish or great britain or great british or NHS or "national health service")

Indexes=SCI-EXPANDED, SSCI, A&HCI, CPCI-S, CPCI-SSH, BKCI-S, BKCI-SSH, ESCI, CCR-EXPANDED, IC Timespan=All years

TOPIC: (((Lay adj (care* or work*)) or volunteer* or (third near/1 sector) or (non-profit* or nonprofit*) or charit* or spouse* or wife* or wives* or husband* or partner* or (home* near/2 (care or residential* or nursing* or (old near/1 (folk* or person* or people*)))) or (primary near/1 (care or healthcare or (health near/1 care))) or (general near/1 practi*) or (family near/1 (practi* or doctor* or physician*)) or (community near/2 (care* or healthcare or nurs* or compassionat*)) or (district* near/1 nurs*) or (marie near/1 curie) or (macmillan near/3 (nurs* or service* or support*))))

Indexes=SCI-EXPANDED, SSCI, A&HCI, CPCI-S, CPCI-SSH, BKCI-S, BKCI-SSH, ESCI, CCR-EXPANDED, IC Timespan=All years

TOPIC: (terminal* or palliative* or hospice* or "end of life" or (last near/2 (week* or day* or month* or year*)))

Indexes=SCI-EXPANDED, SSCI, A&HCI, CPCI-S, CPCI-SSH, BKCI-S, BKCI-SSH, ESCI, CCR-EXPANDED, IC

Timespan=All years

PsycINFO

#	Query
S10	S3 AND S7 AND S8 AND S9
S9	theor* or model* or concept* or philosoph* or program*
S8	gb or "g.b." or britain* or british or uk or "u.k." or united kingdom* or england or northern irish or northern ireland or scotland or scottish or wales or welsh
S7	S4 OR S5 OR S6
S6	((((((DE "Volunteers") OR (DE "Nonprofit Organizations")) OR (DE "Spouses" OR DE "Husbands" OR DE "Wives")) OR (DE "Home Care")) OR (DE "Nursing Homes")) OR (DE "Residential Care Institutions" OR DE "Halfway Houses" OR DE "Hospitals" OR DE "Nursing Homes" OR DE "Orphanages")) AND (DE "Community Services" OR DE "Community Mental Health Services" OR DE "Community Welfare Services" OR DE "Crisis Intervention Services" OR DE "Home Visiting Programs" OR DE "Public Health Services" OR DE "Community Welfare Services")
S5	((((DE "Primary Health Care") OR (DE "Community Health" OR DE "Community Mental Health")) OR (DE "Community Services" OR DE "Community Mental Health Services" OR DE "Community Welfare Services" OR DE "Crisis Intervention Services" OR DE "Home Visiting Programs" OR DE "Public Health Services")) OR (DE "Home Care")) OR (DE "General Practitioners")
S4	((Lay n/1 (care* or work*)) or volunteer* or (third n/1 sector) or (non-profit* or nonprofit*) or charit* or spouse* or wife* or wives* or husband* or partner* or (home* n/2 (care or residential* or nursing* or (old n/1 (folk* or person* or people*)))) or (primary n/1 (care or healthcare or (health n/1 care))) or (general n/1 practi*) or (family n/1 (practi* or doctor* or physician*)) or (community n/2 (care* or healthcare or nurs*

	or compassionat*)) or (district* n/1 nurs*) or (marie adj curie) or (macmillan n/3 (nurs* or service* or support*)))
S3	S1 OR S2
S2	"end of life" or palliative* or terminal* or hospice* or (last n/1 (week* or month* or year* or day*))
S1	((DE "TerE "Palliative Care")) OR (DE "Hospice")

CINAHL

#	Query
S11	S3 AND S6 AND S9 AND S10
S10	theor* or model* or concept* or philosoph* or program*
S9	S7 OR S8
S8	(MH "United Kingdom+")
S7	gb or "g.b." or britain* or british or uk or "u.k." or united kingdom* or england or northern irish or northern ireland or scotland or scottish or wales or welsh
S6	S4 OR S5
S5	(MH "Family Practice") OR (MH "Charities") OR (MH "Voluntary Health Agencies+") OR (MH "Volunteer Workers") OR (MH "Spouses") OR (MH "Home Health Care+") OR (MH "Home Nursing") OR (MH "Nursing Home Personnel") OR (MH "Nursing Homes+") OR (MH "Community Health Nursing+") OR (MH "Residential Care+") OR (MH "Nursing Home Patients")

S4	(MH "Primary Health Care") OR (MH "Community Health Centers+") OR (MH "Community Health Services+") OR (MH "Community Health Nursing+") OR (MH "Home Health Care+") Or ((Lay n/1 (care* or work*)) or volunteer* or (third n/1 sector) or (non-profit* or nonprofit*) or charit* or spouse* or wife* or wives* or husband* or partner* or (home* n/2 (care or residential* or nursing* or (old n/1 (folk* or person* or people*)))) or (primary n/1 (care or healthcare or (health n/1 care))) or (general n/1 practi*) or (family n/1 (practi* or doctor* or physician*)) or (community n/2 (care* or healthcare or nurs* or compassionat*)) or (district* n/1 nurs*) or (marie adj curie) or (macmillan n/3 (nurs* or service* or support*)))
S3	S1 OR S2
S2	"end of life" or palliative* or terminal* or hospice* or (last n/1 (week* or month* or year* or day*))
S1	(MH "Terminal Care (Saba CCC)+") OR (MH "Terminal Care+") OR (MH "Terminally Ill Patients+") OR (MH "Palliative Care") OR (MH "Hospice and Palliative Nursing") OR (MH "Hospice Care") OR (MH "Hospices")

Sociological Abstracts

(ab(theor* OR model* OR concept* OR philosoph* OR program*) OR ti(theor* OR model* OR concept* OR philosoph* OR program*))

AND

(ab(gb or "g.b." or britain* or british or uk or "u.k." or united kingdom* or england or northern irish or northern ireland or scotland or scottish or wales or welsh) OR ti(gb or "g.b." or britain* or british or uk or "u.k." or united kingdom* or england or northern irish or northern ireland or scotland or scottish or wales or welsh))

AND

(ab((terminal* OR palliative* OR "end of life" OR hospice* OR "last week*" OR "last month*" OR "last day*")) OR ti((terminal* OR palliative* OR "end of life" OR hospice* OR "last week*" OR "last month*" OR "last day*")))

AND

((MAINSUBJECT.EXACT("Nursing Homes") OR MAINSUBJECT.EXACT("Health Care Services") OR MAINSUBJECT.EXACT("Volunteers") OR MAINSUBJECT.EXACT("Primary Health Care") OR MAINSUBJECT.EXACT("Spouses") OR MAINSUBJECT.EXACT("Home Care") OR MAINSUBJECT.EXACT("Charities")) OR ab((general practice OR family practice OR community care OR community healthcare OR district nurse OR home care OR spouse OR charity OR volunteer)) OR ti((general practice OR family practice OR community care OR community healthcare OR district nurse OR home care OR spouse OR charity OR volunteer)))

ASSIA

(MAINSUBJECT.EXACT.EXPLODE("Palliative Care") OR MAINSUBJECT.EXACT.EXPLODE("Terminal Illness") OR MAINSUBJECT.EXACT.EXPLODE("Hospices") OR (terminal* or palliative* or "end of life" or hospice* or "last week*" or "last month*" or "last day*"))

AND

(ab(gb or "g.b." or britain* or british or uk or "u.k." or united kingdom* or england or northern irish or northern ireland or scotland or scottish or wales or welsh) OR ti(gb or "g.b." or britain* or british or uk or "u.k." or united kingdom* or england or northern irish or northern ireland or scotland or scottish or wales or welsh))

AND

(ab(theor* OR model* OR concept* OR philosoph* OR program*) OR ti(theor* or model* or concept* or philosoph* or program*))

AND

((MAINSUBJECT.EXACT.EXPLODE("Nursing homes") OR MAINSUBJECT.EXACT.EXPLODE("Private nursing homes")) OR MAINSUBJECT.EXACT.EXPLODE("Charity") OR (MAINSUBJECT.EXACT.EXPLODE("District nurses") OR MAINSUBJECT.EXACT.EXPLODE("Long term residential care") OR MAINSUBJECT.EXACT.EXPLODE("Spouses") OR MAINSUBJECT.EXACT.EXPLODE("Private residential care")) OR (MAINSUBJECT.EXACT.EXPLODE("Volunteer support services") OR MAINSUBJECT.EXACT.EXPLODE("Community volunteers") OR MAINSUBJECT.EXACT.EXPLODE("Volunteers")) OR (general practice OR family practice OR community care OR community healthcare OR district nurse OR home care OR spouse OR charity OR volunteer))

SCIE Social Care Online

palliative or hospice or terminal* or "end of life" or "last week" or "last day" or "last month"

and

primary care or general practice or general practitioner or community care* or home care or district nurse or marie curie or macmillan or charity or charities or volunteer or voluntary or nursing home or care home or home for the aged or residential care

and

theory or model or programme or program or concept or philosophy

Targeted search on systematic reviews on prognosis in palliative and end of life care and/or predictions of survival or death

Database: Ovid MEDLINE(R) and Epub Ahead of Print, In-Process & Other Non-Indexed Citations, Daily and Versions(R) <1946 to January 27, 2020>

Search Strategy:

----

1 (predict* or prognos*).mp. [mp=title, abstract, original title, name of substance word, subject heading word, floating sub-heading word, keyword heading word, organism supplementary concept word, protocol supplementary concept word, rare disease supplementary concept word, unique identifier, synonyms] (2225603)

2 (survival or "end of life" or "imminent death").mp. [mp=title, abstract, original title, name of substance word, subject heading word, floating sub-heading word, keyword heading word, organism supplementary concept word, protocol supplementary concept word, rare disease supplementary concept word, unique identifier, synonyms] (1221447)

3 (surprise* adj2 question).mp. [mp=title, abstract, original title, name of substance word, subject heading word, floating sub-heading word, keyword heading word, organism supplementary concept word, protocol supplementary concept word, rare disease supplementary concept word, unique identifier, synonyms] (89)

4 1 and 2 and 3 (29)

5 (review* or systematic*).mp. [mp=title, abstract, original title, name of substance word, subject heading word, floating sub-heading word, keyword heading word, organism supplementary concept word, protocol supplementary concept word, rare disease supplementary concept word, unique identifier, synonyms] (3802284)

6 4 and 5 (4)

7 from 6 keep 1-4 (4)

8 2 and 3 (40)

9 5 and 8 (6)

10 9 not 7 (2)

11 from 10 keep 2 (1)

12 1 and 2 and 5 (77630)

13 ((predict* or prognos*) and (survival or "end of life" or "imminent death") and (review* or systematic*)).ti,ab. (45573)

14 ((predict* or prognos*) and (survival or "end of life" or "imminent death") and (review* or systematic*)).ti. (203)

15 14 not 9 (203)

16 limit 15 to yr="2000 -Current" (188)

17 7 or 11 (5)

18 3 and 5 (11)

19 18 not 9 (5)

20 from 16 keep 1-188 (188)

21 from 17 keep 1-5 (5)

22 from 19 keep 1,3-5 (4)

Revised Date: 07/2015

CINAHL, EBSCOhost

#	Query	Results
S8	(TI ((predict* or prognos*)) AND ((survival or "end of life" or "imminent death") AND ((review* or systematic*))) AND TI palliative	33
S7	TI ((predict* or prognos*)) AND ((survival or "end of life" or "imminent death") AND ((review* or systematic*))	2,945
S6	TI ((predict* or prognos*)) AND ((survival or "end of life" or "imminent death") AND ((review* or systematic*))	3,016
S5	((predict* or prognos*)) AND ((survival or "end of life" or "imminent death") AND ((review* or systematic*))	12,660
S4	S2 AND S3	16
S3	review* or systematic*	645,736
S2	surpris* n3 question*	97
S1	surprise n3 question*	77
