# Supplementary material for: Timely community palliative and end-of-life care: a realist synthesis
Source: BMJ Support Palliat Care. 2021 Dec 9;14(e3):e003066. doi: 10.1136/bmjspcare-2021-003066 (PMC11671952; doi:10.1136/bmjspcare-2021-003066)
Supplement: online supplemental appendix 5 [file bmjspcare-14-e3-s005.pdf]

Appendix 5: Further detail on programme types

Box 3, complete version: Types of palliative and end of life care programmes in primary care and the community in terms of broad programme logic expressed as CMO-configurations

Note: Here we offer a typology of palliative and end of life care programmes in primary care and the community in terms of the overarching, generic theory (taking the form of a CMO-configuration) to which they appear to subscribe. We have abstracted the high-level, generic CMOs from specific CMOs characterising the example programmes analysed, with the examples coming from the 253 "core contents" citations.

For instance, on the basis of the brief programme descriptions we have reviewed, we suggest that the abstract mechanism underpinning innovative discharge roles is the management of boundaries between services and settings. Similarly, we suggest that intermediate care beds can be thought of as a boundary management initiative – both between service types (hospital and community) and patient needs (requiring intense professional input and oversight – requiring more limited professional input and oversight). Thus, two programmes which may "look" very different belong, in our classification, to the same type by virtue of their shared theoretical underpinning – shared programme logic of mechanisms causing their outcomes. Much further work is required to elicit the theories behind the specific programmes included here and to test our choices of a "defining theory".

Type 1 programmes: Programmes seeking to improve the availability of services where systemic and critical gaps exist: in terms of time, location, intensity and responsiveness (when, where, how much and how quick)

When palliative care is needed in time periods outside of normal working hours and in underserved areas (C), end of life care outcomes will improve dramatically and efficiently (O) only if services are time-of-the-day-independent (M), adapted to the location where they are offered (M), flexible (M) and responsive (M).

Examples of Type 1 programmes that address time and timing

24/7 services
Out of hours (OOH) services
- OOH specialist palliative and end of life care services
- OOH pharmacy provision of drugs
- OOH generalist services
- "Informal" OOH services (e.g. GP providing personal phone number)

"Twilight" services (in the underserved periods between daytime and OOH services)
Night services
- Night nursing
- Night sitting

Hospice day care services
Rapid response services

Examples of Type 1 programmes that address location, coverage and proximity

Home-based services, including Hospice at Home
Hospice outpatient services
GP practice palliative and end of life care clinics
Community centres services
Host family respite
Rural services
Telecare services
Deprived areas services
(relative) Services moving closer to the users

Examples of Type 1 programmes that address intensity and responsiveness

Rapid response services
Improved standard practice (new types of prioritisation)

Type 2 programmes: Programmes defined through the broad aspect of our humanity and needs being addressed, often as forms of care and support provided by a particular professional or lay group

As suffering and pain are multimodal (C), we can achieve better quality of life for dying patients (O) when we acknowledge the numerous modalities of experiencing pain and suffering and by acting in (more) holistic ways (M).

Subtypes of Type 2 programmes in terms of the form of therapy or support offered

Alternative and complementary therapies
Arts and art therapy
Basic body comfort
- Hands-on enablement
- Equipment-enabled comfort

Basic human presence and support – being with, staying with; bridging the "dying world" and the "world outside"; small, intangible acts of kindness
Beauty and wellness
Clinical and medical care
Financial support and advice
Hands-on care
Holistic care
Movement, exercise, physiotherapy
Nature, gardens, the outdoors
Personal care support
Psychological support and counselling
Rehabilitation
Respite
- Day care respite
- Host family respite
- In-home respite
- Institutional respite
- Video respite

Social support
- Opportunities for meeting people
- Social activities
- Social environment as created by volunteers
- Social support from fellow patients

Type 3 programmes: Programmes addressing the management of boundaries and transitions

As the needs of dying patients at transition points can be extra complex (C) and different services often lack sufficient levels of integration and coordination (C), we can enable each patient to receive the most appropriate and timely care within resource limits (O) if we manage service boundaries and transitions better, in rational yet person-centred ways (M).

Examples of Type 3 programmes that address discharge management
- Rapid hospital discharge to enable home death
- Discharge roles (e.g. Discharge Community Link Nurses)
- Discharge letters and templates
- Discharge policies and pathways
- Discharge practices when palliative care needs reduced or prognosis modified

Examples of Type 3 programmes that address referral management
- Clarification of referral criteria (triggers) and development of documentation
- Rules on referral initiators – who can refer?
- Rules on referral timing – when to refer?
- Referral triggers – what needs to happen so as to refer?
- Referral audits for quality improvement

Examples of Type 3 programmes that address "midway solutions" between service types
- Intermediate care beds
- Community hospitals
- Hospice at Home services
- Primary care doctors with visiting rights to local hospitals

Examples of Type 3 programmes that address the management of transitions and working across settings
- "Alignment models", e.g. aligning the work of GPs and care homes
- Bridging roles – liaison roles, secondments, dual roles
- Case reviews across settings
- Electronic data sharing, Electronic Palliative Care Coordination Systems (EPaCCS)
- Hand-over protocols and forms, particularly for OOH
- Multidisciplinary team meetings
- Service integration work
- Transportation across settings
  - Partnerships between ambulance service and other settings

Type 4 programmes: Programmes prioritising patient-centredness, ownership and empowerment

As patients and their carers have a range of diverging end of life care needs, preferences and wishes (C), we are far more likely to achieve the goals of care that truly matter to them (O) if these are clearly elicited, recorded and acted upon (M) and, more broadly, if services are co-developed with patients and carers (M).

Subtypes of Type 4 programmes with examples

Programmes centred around tools for eliciting and recording patient preferences and wishes for end of life care
- Preferences elicitation and care planning tools – e.g. Preferred Priorities of Care, EPaCCS, ReSPECT
- Legally binding tools – e.g. Advanced Directives to Refuse Treatment (ADRT)
- Design of new tools
- Training and support for the use of existing tools
- Broader initiatives for increasing the uptake of such tools

Programmes based on shared decision-making innovations
- Patient-led case conferences

Programmes based on support for self-management

Programmes for co-developing services with patients

Peer support programmes
- Peer education programmes for older people about Advance Care Planning

Programmes for community-based discussions of end of life care

Type 5 programmes: Programmes addressing different phases of an illness or of the dying process

As the phase in the trajectory of an illness and/or the proximity to death of a patient has a profound impact on their treatments and care needs (C), we can improve outcomes for patients and support the sustainability of the health service (O) by structuring and delivering services in a phase-centric way which enables their optimisation, with no relevant needs missed and no unnecessary activities undertaken (M).

Subtypes of Type 5 programmes (perhaps more often presenting as service structures and pathways than new programmes)

Services around the delivery of a terminal diagnosis
Services during active treatment
Services after discharge
Services at recurrence/ exacerbation
Services in the final weeks and days of life
Anticipatory bereavement
Bereavement support

Type 6 programmes: Programmes taking a systemic approach

As the terminal phase of an illness or the process of dying can be very complex and fast changing and involve a large number of services (C), we are more likely to achieve positive outcomes for the patients, their family and the system (O) and less likely to encounter crisis situations (O) if terminal illness and/or the process of dying is approached in a systematic, proactive and anticipatory manner rather than a piecemeal and reactive one (M).

Subtypes of Type 6 programmes with examples

Programmes aiming to improve identification of patients in need of palliative and/or end of life care
- Development of new prediction and risk stratification tools
- Broader, more systematic implementation of prediction and risk stratification tools
- Improving staff abilities in identifying patients at the end of life
- Improving the skills of junior and lower level staff in communicating concerns about patients higher up the hierarchy
- Appropriate recording and communication of such information to other services, e.g. through Registers (EPaCCS)

Programmes enabling discussions of death, dying and care at the end of life

Programmes aiming to improve Advance Care Planning (ACP)
- Tools, proformas, templates
- Enhancing basic staff skills in using them
- More in-depth training on using ACP tools, acknowledging challenges such as differences between family and patient preferences, dynamics of preferences, service limitations, creating the right environment for the conversations, etc.
- Initiatives to support the broader, more systematic use of such tools

Programmes aiming to improve integration of care and handling diffusions of responsibility
- Case management initiatives
- Key worker initiatives
- Data sharing for improved informational continuity
- Palliative care coordination centres
- Bridging roles

Programmes aiming to develop or refine existing protocols and pathways

Programmes based on the use of decision-making tools

Programmes aiming to improve monitoring and evaluation systems and processes
- Enhanced annual reviews of patients
- Patient recall systems and processes
- Palliative and end of life care registers and dashboards
- Provision of (comparative) data on palliative and end of life care processes and outcomes

Programmes facilitating internal change through external support
- Peer facilitation for practices
- Educational facilitation

Programmes creating a broad supportive environment
- Financial incentives
- National guidance
- Local change management initiatives
- Identifying and supporting ‘champions’

“Meta-programmes” – highly systematic ways of developing new local initiatives and programmes

Type 7 programmes: Programmes seeking improvements through staff and volunteer development

When work environments value palliative and end of life care training and development as part of their business-as-usual rather than a matter of short-term projects (C), palliative and end of life care provision across the board improves (O) through investment in the knowledge, skills, motivation, attitudes, etc. of professionals and lay persons providing care (M) and through creating effective role structures and arrangements (M).

Subtypes of programmes and examples

Programmes based on developing new staff roles and forms of task distribution

- Extended nurse prescribing in palliative care
- Peer facilitators with 'dual roles' (e.g. GPs with special interest in PEoLC)
- Bridging roles

Programmes for staff training and support

- From palliative care specialists to generalist staff
- Training in specific skills
  - Communication
  - Palliative and end of life care prescribing
  - Advance Care Planning
  - etc.
- Training for specific staff groups
- Training by using different approaches, contexts and platforms (hands-on, online, on-the-job, etc.)
- Support for generalist staff, or even specialist staff, in dealing with rare diseases

Programmes expanding the roles of volunteers and community members

- Compassionate cities initiatives
- Volunteers in hospices
- Death dealers

Type 8 programmes?: Programmes defined through the support they provide to informal carers?

The presence of carer-focused programmes in the research literature, as sampled, was not on a par with the presence of programmes of the other seven types. Further research is needed on the degree to which support for informal carers in palliative and end of life care translates into programmes which are defined in terms of their carer-focused mechanisms rather than including them as a secondary component.
