# Supplementary material for: Timely community palliative and end-of-life care: a realist synthesis
Source: BMJ Support Palliat Care. 2021 Dec 9;14(e3):e003066. doi: 10.1136/bmjspcare-2021-003066 (PMC11671952; doi:10.1136/bmjspcare-2021-003066)
Supplement: online supplemental appendix 6 [file bmjspcare-14-e3-s006.pdf]

# Appendix 6: CMO-configurations and supporting evidence on the identification of patients at the end of life, short-term prognosis and associated referrals

The Appendix has 3 parts:

- Excerpts from UK policy documents and reports concerning the identification of end of life care (EoLC) patients
- Supplementary Table 1: Context-Mechanism-Outcome configurations around identification, prognosis and referrals based on them
- Supplementary Table 2: Abstract-level scoping of most recent (last 5 years) systematic reviews on prognosis

## 1. Excerpts from UK policy documents and reports concerning the identification of end of life care (EoLC) patients

The list of excerpts is illustrative. The documents chosen were considered as highly likely to have had significant impact on palliative and end of life care services and practice in England, by virtue of being prepared by broad coalitions of UK palliative and end of life care organisations; the UK Government/ Department of Health and Social Care; the UK Parliament; and NICE, the National Institute for Health and Care Excellence (a non-departmental public body of the UK Department of Health and Social Care, which provides national guidance and advice to improve health and social care).[1,2] The excerpts are also illustrative for each of the documents. We have selected 2-7 claims per source which represent its position and which were, ideally, succinct and focused on identification and prognosis only.

Even if far from exhaustive, we believe the list gives a sufficiently reliable overview of the mainstream position around patient identification in current UK palliative and end of life care policy and practice. We have also made sure to include caveat statements (around the challenges of identification and uncertainty at the end of life), even if they are not too prominent in a document. They are indicated by (uncertainty) preceding a statement. Our arguments criticising the mainstream view concern the lack of sufficient recognition of the challenges of prognosis and identification rather than a complete lack of it. However, this lack of balance, we claim, leads to a range of important unintended negative consequences.

The documents are arranged in chronological order. Statements are direct quotes (indicated by italics; or if the document quotes a contributor, by italics and inverted commas, with the source added). Minimal connecting or clarifying statements have been added by us (normal font).

Q followed by a number (e.g. Q7) is a unique identifier for quotes referred to in the table of Context-Mechanism-Outcome configurations further below.

---

[1] https://en.wikipedia.org/wiki/National_Institute_for_Health_and_Care_Excellence

[2] https://www.nice.org.uk/about

## Department of Health. End of Life Care Strategy (Jul 2008)[3]

Q 1. *The* [EoLC] *care pathway involves the following steps:*

- *Identification of people approaching the end of life and initiating discussions about preferences for end of life care;*

  [6 further steps follow] (p. 9-10)

Q 2. *[M]any health and social care staff have had insufficient training in identifying those who are approaching the end of life, in communicating with them or in delivering optimal care. To address this, a major workforce development initiative is now needed, with particular emphasis on staff for whom end of life care is only one aspect of their work.* (p. 12)

Q 3. *(*uncertainty) *The definition of the beginning of end of life care is variable according to individual person and professional perspectives.* (p. 47)

Q 4. *The major challenges in relation to the delivery of high quality end of life care in the community include:*

- *Poor identification, assessment and coordination of end of life care within some general practices;*

  [6 further challenges follow] (p. 84)

Q 5. (uncertainty) *It is difficult, if not impossible, to calculate the cost of end of life care in this country. This is partly because of the difficulty in defining exactly when end of life care starts.* (p. 150)

---

[3] Department of Health. *End of Life Care Strategy: Promoting high quality care for all adults at the end of life.* Jul 2008. https://assets.publishing.service.gov.uk/government/uploads/system/uploads/attachment_data/file/136431/End_of_life_strategy.pdf (Accessed Feb 2020)

## House of Commons Health Committee report (Mar 2015)[4]

*Q 6. The situation is unlikely to improve unless clinicians feel confident to identify people who may be near the end of life and to start conversations with their patients about their wishes.* (p. 3, Summary)

*Q 7. "The NHS continues to struggle to properly identify people who are in the last stages of life, unless they have a terminal or otherwise life-threatening condition … This in turn means people are not able to plan properly or encouraged to discuss decisions about their future care … A failure to understand multi-morbidity and frailty and the trajectory of people's health living with either (or often both) means the NHS is not agile, failing to allow care and support to switch tracks quickly when someone is approaching the end of life."* (from Age UK written evidence to inquiry; p. 14)

*Q 8. The Sue Ryder Care Centre for the Study of Supportive, Palliative and End of Life Care has also found that staff caring for frail and seriously ill older people have difficulty recognising the point at which a transition to a palliative care approach may be appropriate.* (p. 14-15)

*Q 9. Many of those who provided written evidence to this inquiry expressed the opinion that health care staff need support to develop their competence in identifying, providing care to and communicating with people at the end of life and their families and carers.* (p. 20)

*Q 10. "Our case work has shown that there is a need to communicate in a way that is both sensitive but also makes clear the prognosis and what options there are for care based on the outcomes individuals may want for themselves."* (evidence provided by the Parliamentary and Health Service Ombudsman; p. 20)

*Q 11. "When patients are not informed about their prognosis and are not asked about their care and medical treatment preferences, they are denied the right to participate in decisions and might not have the death they want."* (evidence provided by Compassion in Dying; p. 21)

*Q 12. Witnesses to the inquiry expressed concern that there is a lack of research evidence to inform the development of models for early identification of those people who would benefit from receiving palliative care and how to deliver palliative and end of life care services to non-cancer patients.* (p. 39)

---

[4] House of Commons Health Committee. *End of Life Care. Fifth Report of Session 2014-15.* Mar 2015. https://publications.parliament.uk/pa/cm201415/cmselect/cmhealth/805/805.pdf (Accessed Feb 2020)

## National Palliative and End of Life Care Partnership. Ambitions for Palliative and End of Life Care: A national framework for local action 2015-2020 (Sep 2015)[5]

*Q 13.* (uncertainty acknowledged; then largely overridden) *We know that much about recognising dying and impending death is uncertain and challenging. However, timely identification and honesty where there is uncertainty is key to the quality of care – all else follows.* (p. 18)

*Q 14. Effective systems for person centred care need to encompass: systematic ways of reaching people who are approaching the end of life, effective assessment as well as effective decision making support, care coordination, care planning, and care delivery.* (p. 19)

## Department of Health. The Government Response to the Review of Choice in End of Life Care (Jul 2016)[6]

*Q 15. Good care is also about identifying people approaching the end of life earlier so that there is time and opportunity to prepare, discuss and plan care, and time to put that care in place to meet people's individual needs and preferences.* (p. 19)

*Q 16.* (uncertainty) *The AMBER care bundle is a simple approach used in hospitals when there is clinical uncertainty of recovery. The approach supports staff, patients and families who wish to continue with treatment in the hope of a recovery, while encouraging open conversation about people's wishes and preferences should things get worse rather than better.* (p. 21)

*Q 17. [A]ll CCGs should also be clear about the efficiency gains to be made when people are able to make plans and express preferences early about their care so that unnecessary hospital admissions are avoided. We know that people's experiences of care are better and services more coordinated and efficient when clinical staff can identify people's needs earlier and share and implement end of life care plans more easily.* (p. 25-26)

---

[5] National Palliative and End of Life Care Partnership. *Ambitions for Palliative and End of Life Care: A national framework for local action 2015-2020*. Sep 2015. http://endoflifecareambitions.org.uk/wp-content/uploads/2015/09/Ambitions-for-Palliative-and-End-of-Life-Care.pdf (Accessed Feb 2020)

[6] Department of Health. *Our Commitment to you for end of life care: The Government Response to the Review of Choice in End of Life Care.* Jul 2016. https://assets.publishing.service.gov.uk/government/uploads/system/uploads/attachment_data/file/536326/choice-response.pdf (Accessed Feb 2020)

## National Institute for Health and Care Excellence guideline for service delivery (Oct 2019)[7]

*Q 18.* (uncertainty) *End of life care includes the care and support given in the final weeks and months of life, and the planning and preparation for this. For some conditions, this could be months or years.* (p. 35)

*Q 19.* First recommendation in Guideline is about: *1.1 Identifying adults who may be approaching the end of their life, their carers and other people important to them*. (p. 7)

*Q 20. People managing and delivering services should develop systems to identify adults who are likely to be approaching the end of their life (for example, using tools such as the Gold Standards Framework, the Amber Care Bundle or the Supportive and Palliative Care Indicators Tool [SPICT]). This will enable health and social care practitioners to start discussions about advance care planning, provide the care needed, and to support people's preferences for where they would like to be cared for and die.* (p. 7)

*Q 21.* (uncertainty acknowledged; then largely overridden) *Although the evidence was limited, the committee agreed that identifying adults who may be approaching the end of their life supports health and social care practitioners to start discussions about advance care planning. This should ensure that the person near the end of life is provided with the support that they may need now or later to help them stay where they would like to be cared for and die. It also gives them time to consider and re-evaluate their needs with their health and social care practitioners.* (p. 22)

*Q 22. The committee wanted to emphasise the importance of identifying people systematically. There are already some systems in use for identifying people approaching the end of their life, which are given as examples in the recommendations. However, there were no studies comparing and evaluating their effectiveness in service delivery so the committee could not recommend a particular system.* (p. 22)

---

[7] National Institute for Health and Care Excellence. *End of life care for adults: service delivery.* NICE guideline [NG 142]. Oct 2019. https://www.nice.org.uk/guidance/ng142 (Accessed Feb 2020).

## 2. Supplementary Table 1: Context-Mechanism-Outcome configurations around identification, prognosis and referrals based on them

*The row below is illustrative to help explain the structure of the table and the abbreviations and symbols used in it, presented in the notes that follow.*

| **Parameter type and identifier** | **Context-Mechanism-Outcome (CMO) configurations or elements** | **Sources** | **Key pieces of evidence, theoretical claims and interpretations** |
|---|---|---|---|
| **PROGNOSIS, TIMELY IDENTIFICATION** | | | |
| **1.1.**<br><br>**A, C, M, O**<br><br>Original programme theory | **Timely identification of end of life stage as enabling preparation**<br><br>The identification of patients who are likely to be in the last year of their lives (A/O $_{intermediate}$)<br><br>allows sufficient time for discussing, planning, organising and coordinating care<br>around a patient's needs, wishes and preferences (M), for instance around their preferred place of care (O);<br>their family's needs and capacity (M/C);<br>the availability of local services (C);<br>and in view of the sustainability of the health system (C).<br><br>In terms of broader life (C), such identification allows … *[continues below]* | Sims-Gould et al. 2010 quoting Steinhauser et al. (trigger)<br><br>Core policy documents (see pp. 2-5 above)<br><br>Roikjær et al. 2019 *** BROADER (hospital)<br><br>Domain knowledge of team | **<u>(A, M, O) Identification enables preparation which, in turn, enables meeting an individual's needs and preferences and achieving a good death</u>**<br><br>Programme theory 'seed' statements<br><br>**Department of Health, 2016. The Government Response to the Review of Choice in End of Life Care**<br><br>"Good care is also about identifying people approaching the end of life earlier so that there is time and opportunity to prepare, discuss and plan care, and time to put that care in place to meet people's individual needs and preferences." (p. 19)<br><br>**National Institute for Health and Care Excellence guideline for service delivery, 2019**<br><br>"People managing and delivering services should develop systems to identify adults who are likely to be approaching the end of their life … *[continues below]* |

## Context-Mechanism-Outcome (CMO) configurations or elements

The CMOs presented in the table can be complete configurations, partial ones (e.g. M-O, C-O) or, occasionally, individual elements (C, M, O). If only the latter, they have been included because they appear to have significant explanatory potential, but either cannot be weaved in a complete CMOs yet or can contribute to many (typically the C-element). At times, A, for programme activity, is also added to make the explanations clearer and better connected to 'visible' features of a programme. Longer CMO-configurations are introduced by a brief heading, which can be based on any of the elements.

## CMO colour coding

**Green CMOs** or elements are the ones intended and (partly) articulated by the original programme theory. The versions offered in the table are refinements of the rough programme theory.

**Black** indicates that particular negative **CMO** configurations are recognised by the original programme theory. In fact, the latter is often developed as an attempt to address them.

**Red** indicates **CMO** configurations which are not, or only minimally, recognised by the original programme theory but limit its applicability. In such configurations, M is a countervailing mechanism, C is a context blocking the functioning of the programme theory mechanisms, and O is a resulting outcome.

**Yellow** is for CMO configurations or elements which are consistent with the original programme theory but are mentioned only minimally and remain mostly tacit.

## Abbreviations

The following are non-standard conceptual subtypes and notations (non-standard in the realist method) we have been experimenting with for additional accuracy of attributions:

A – programme activity

$A_{fail}$ – used to indicate a failure to perform a programme activity

$O_{multiple}$ – sets of outcomes which tend to co-occur

$O_{intermediate}$ – indicates outcomes that are elements of a chain of steps and dependencies which may lead to primary outcomes. Primary outcomes may be patient-relevant, carer-relevant, health professionals-relevant and health system-relevant.

M/O, C/M or other combinations – when an element can perform more than one formal function, depending on the perspective.

### Symbols differentiating reference types (sources column)

**Asterisks** indicate a process of literature searching and identification other than the core search (no asterisk means the reference is from the core search):

> * indicates references from a focused, targeted search – searches conducted with the goal of further specifying and testing the theories under development (search strategies in the Literature searching appendix);
> ** indicates references obtained through citation tracking – following up on work referenced in the papers we have been analysing or using forward citation tracking (looking up papers which cite the one being reviewed);
> *** indicates a reference from the pre-existing collections of the authors, recommendations from colleagues or serendipitously identified papers.

**BROADER** indicates that the source did not fall fully within the scope of the review in terms of its theme (palliative and end of life care), geography (England, potentially UK) and population (adults). Using such broader resources is entirely in line with the realist approach and, in fact, an expression of its exploratory logic. The particular "deviation" from an exemplary source is shown in brackets:

- BROADER (country – e.g. Australia)
- BROADER (domain – e.g. mental health)
- BROADER (population – e.g. children).

**Sims-Gould et al. 2010 (trigger)** – i.e. smaller font sources followed by "(trigger)" were the first sources to prompt us to code an issue, but the latter was a relatively marginal point in them. Typically, we have sought further sources to refine the CMO-configurations of interest and to substantiate them with evidence/ theoretical claims.

> The realist approach relies heavily on the so called "nuggets of evidence". One way to describe them is as minor, peripheral claims or data in a source document, which, however, provide a pertinent confirmation/ refutation of a hypothesis from the realist study. The awareness of the value of nuggets of evidence meant that, especially early on, we have been coding minor mentions of issues because of their relevance rather than richness and depth.

Furthermore, most primary care and community interventions in palliative and end of life care are complex interventions, meaning they have many elements, many of which do not receive focused attention in a publication.

Finally, publications in any research field have a sufficiently shared discourse and background. Core ideas emerge quickly from any set of publications. Which came first is a matter of the approach to literature searching and the chronology of analysis. Also, any researcher familiar with the domain (and we are) can formulate a broad range of statements from its discourse provided a trigger.

Such sources are included for transparency and to support explorations of the potential impact on the review outcomes of the literature searching approach and chronology of reading/coding.

**Domain knowledge of team** – occasionally, this has been added to the source column. Some CMO-configurations were refined or expanded through the background knowledge of the domain experts on the team (SB and MP for PEoLC and SB and GW for general practice). If a statement appears in the CMO-configuration without a corresponding evidence in the last column, then it was based on such broader domain knowledge. This has only been done for claims we consider uncontroversial, "givens" in the field of (UK) palliative and end of life care, and which, precisely because of being taken for granted by insiders, are not always made explicit.

## Key pieces of evidence, theoretical claims and interpretations

If the evidence, theoretical claims and interpretations in the last column are coming from an empirical study and from its own data (rather than its background literature review, for instance), we have included a brief description of its
*(Setting/ Participants) and*
*(Methods)*
to help readers evaluate the credibility of the evidence and its relevance to their own work. If the authors' description is not transparent enough of important study limitations, we add further critical comments and caveats to the brief Methods description. These setting/participants and methods overviews have been developed in a holistic way, e.g. without the use of a systematic checklist to assess or include parameters.

When the supporting statements added to this column are not empirical evidence or if, while based on evidence, make a significant leap from it (as in the discussion section of a paper, for instance), we have added an indication:

- Programme theory 'seed' statements – for excerpts from policy documents or reports we have used as a 'seed' to grow a rough programme theory. They are relevant evidence in the sense of serving to demonstrate the validity of our rough programme theory. They are often based on some evidence, but this is rarely made explicit in the statement.
- Explanation for pattern of data – typically offered by the authors of a paper. It is based on evidence but makes a theoretical leap from it.
- Framing sections evidence/ statement – evidence or statements which come from the background (introduction) or discussion of a paper. As they are presented briefly, they often lack detail and indications of the trustworthiness of the original source.

| Parameter type and identifier | Context-Mechanism-Outcome (CMO) configurations or elements | Sources | Key pieces of evidence, theoretical claims and interpretations |
|---|---|---|---|
| **PROGNOSIS, TIMELY IDENTIFICATION** | | | |
| **1.1.**<br><br>**A, C, M, O**<br><br>Original programme theory | **Timely identification of end of life stage as enabling preparation**<br><br>The identification of patients who are likely to be in the last year of their lives (A/O $_{\text{intermediate}}$)<br><br>allows sufficient time for discussing, planning, organising and coordinating care<br>around a patient's needs, wishes and preferences (M), for instance around their preferred place of care (O);<br>their family's needs and capacity (M/C);<br>the availability of local services (C);<br>and in view of the sustainability of the health system (C).<br><br>In broader life, outside of the contexts of healthcare (C), such identification allows both patients and carers to prepare for death and develop a level of acceptance of it (M/O), such as through<br>putting their affairs in order (M/O),<br>making the best of the time they have got left (M/O),<br>completing what has been left undone or finding better closure for it (M/O),<br>settling and healing relationships (M/O),<br>expressing love, regret, forgiveness, gratitude, appreciation … (M), | Sims-Gould et al. 2010 quoting Steinhauser et al. (trigger)<br><br>Core policy documents (see pages 2-5 above)<br><br>Roikjær et al. 2019 *** BROADER (hospital)<br><br>Domain knowledge of team | **(A, M, O) Identification enables preparation which, in turn, enables meeting an individual's needs and preferences and achieving a good death**<br><br>Programme theory 'seed' statements<br><br>**Department of Health, 2016. The Government Response to the Review of Choice in End of Life Care**<br><br>"Good care is also about identifying people approaching the end of life earlier so that there is time and opportunity to prepare, discuss and plan care, and time to put that care in place to meet people's individual needs and preferences." (p. 19)<br><br>**National Institute for Health and Care Excellence guideline for service delivery, 2019**<br><br>"People managing and delivering services should develop systems to identify adults who are likely to be approaching the end of their life (for example, using tools such as the Gold Standards Framework, the Amber Care Bundle or the Supportive and Palliative Care Indicators Tool [SPICT]). This will enable health and social care practitioners to start discussions about advance care planning, provide the care needed, and to support people's preferences for where they would like to be cared for and die." (p. 7) |

| | | | |
|---|---|---|---|
| | reminiscing about their life (M) and finding a sense of value and meaning in it (M/O), and<br>leaving a legacy for future generations (M/O).<br><br>Identification of the end of life stage (A) is thus crucial for enabling the best possible outcomes for the patient, their family and other people important to them, and for the sustainability of the health system (e.g. by avoiding unnecessary admissions and interventions) ($O_{multiple}$). | | **See also the policy quotes on pages 2-5, in particular Q7, Q11, Q17 and Q21.**<br><br>**<u>(M, O) Having time to prepare for death and reflect on one's life seen as aspects of a good death by patients and carers</u>**<br><br>**Sims-Gould et al. 2010**<br><br>"Steinhauser et al. (5) studied the perspectives of patients and their families on a good death and found that it would include: completion (adequate time to prepare for death); pain and symptom management; clear decision making; affirmation of the whole person; the opportunity to reflect on one's own personal accomplishments and time to be with others."<br><br>**<u>(M, $O_{intermediate}$) Positive non-medical ways of preparing for death and positive experiences/ outcomes that may result from them</u>**<br><br>**Roikjær et al. 2019**<br><br>"The interventions [using personal narratives in palliative care] were all relatively brief psychotherapy based on reminiscence theory in which patients re-evaluate past experiences with the intention of resolving and integrating past conflicts, bringing new significance and meaning to their life[44] and empirical work on either dignity or end-of-life experience.[45]"<br><br>"The framework underlying dignity therapy and legacy building was the model of dignity,[45,46] which focuses on dignity-conserving tasks such as settling relationships, sharing words of love and preparing legacies of memory and shared values."<br><br>"[The] outlook model has tasks very similar to the dignity model although they name them development tasks: sense of completion in |

| | | | |
|---|---|---|---|
| | | | relationships with family, expressions of regret and forgiveness, acceptance of gratitude and appreciation and sense of meaning of one's life."<br><br>"All interventions have the sense of meaning as a core concept. Furthermore, all interventions share the underlying assumption that these tasks can be facilitated through the personal narrative with components of life review, forgiveness and legacy and positively affect the outcomes of quality of life, psychosocial and existential distress." |
| **1.2.**<br><br>**A, C, M, O**<br><br>Original programme theory<br><br>Functional equivalent of 1.1, negative framing | **Dangers of delayed identification of the end of life stage**<br><br>Delayed identification of the end of life stage ($A_{fail}$/$O_{intermediate}$) may mean that patients, families and other people close to them, as well as health professionals, do not have time to prepare for the death (M).<br><br>Patients may then<br>feel robbed of time they believed they had (M/O),<br>be denied the opportunity to have a choice in how they die (M/O),<br>experience severe distress (O) and, ultimately,<br>not have the death they wanted (O).<br><br>In the context of overburdened services (C) and lack of awareness of a patient's preferences (M),<br>health professionals<br>may not make the best decisions (M), even when trying to do their best (M/C) and, for instance,<br>undertake unnecessary interventions (M/O) or<br>not be able to arrange the right services in time (M/O).<br>This may result in suboptimal care; bad, even horrible, deaths; sense of guilt and frustration for the | Mintzer and Zagrabbe 2007 * BROADER (United States) (trigger)<br><br>House of Commons 2015<br><br>Department of Health 2008<br><br>Domain knowledge of team | **(A, M, O) Lack of timely identification and/or communication of prognosis deprives patients of choice at the end of life and may lead to a bad death**<br><br>Programme theory 'seed' statement<br><br>**House of Commons Health Committee report, 2015**<br><br>"When patients are not informed about their prognosis and are not asked about their care and medical treatment preferences, they are denied the right to participate in decisions and might not have the death they want." (p. 21; evidence provided by *Compassion in Dying*)<br><br>**(A, M, O) Lack of timely identification and/or communication of prognosis creates hard-to-manage uncertainty for families and other people close to the dying person and deprives them of choices they should have had**<br><br>Programme theory 'seed' statements<br><br>**Department of Health. End of Life Care Strategy, 2008**<br><br>"Although not directly told, Keith's wife suspected that his life was limited. As a result she was unsure about contacting her four children. |

| | | | |
|---|---|---|---|
| | health professionals; unnecessary costs and litigation for the health system (O $_{multiple}$).<br><br>Families and other people close to the patient may also<br>feel robbed of time and choice they feel they should have had (M/O),<br>go through avoidable distress and traumatic experiences around the time of death (O) and<br>be left to deal with feelings such as guilt and complicated grief for years to come (O). | | She also struggled with the decision about informing Keith's priest, as she did not want to alarm her husband but at the same time she knew he would wish to see a priest. She also felt upset and cheated that she had not been able to say goodbye to her husband before he was re-ventilated." (p. 25)<br><br>**House of Commons Health Committee report, 2015**<br><br>"The decisions made by healthcare professionals about a patient's care are often made with the best intentions and to try to ensure the highest care quality. However, in cases where decisions are made without the opportunity for the patient and their family to have their preferences assessed fairly, the patient ends up feeling distressed by their lack of choice, and that they have received a poor service." (p. 24; evidence provided by *The Parliamentary and Health Service Ombudsman*)<br><br>**(A, M, O) Lack of timely identification leads to delayed referrals to specialist care. As a result, patients cannot be provided with optimal support. This is also frustrating for PEoLC practitioners.**<br><br>Framing sections evidence/ statement<br><br>**Mintzer and Zagrabbe 2007**<br><br>"Often, hospice is called in very late, within the last week or two of life—sometimes the last day or two—when optimal support and preparation for death cannot be provided. Such late referrals are frustrating to practitioners who deal with end-of-life care." |

| | | | |
|---|---|---|---|
| **1.3.**<br><br>**A, C, M, O**<br><br>Original programme theory | **Limitations of knowledge, skills and information as a core reason for delayed identification**<br><br>Delays and omissions in identifying patients who may be approaching the end of their lives ($A_{fail}$/$O_{intermediate}$)<br><br>are often a matter of insufficient training in palliative and end of life care (M),<br>insufficient experience (M),<br>insufficient knowledge of/ information about a particular patient shared by other colleagues within the health system (M).<br><br>They are also more likely in diseases other than cancer (C), as their disease trajectories are less predictable (M). | Department of Health 2008<br><br>House of Commons 2015<br><br>Domain knowledge of team | <u>**(A/O, M) Failures of identification often result from the lack of knowledge, skills and training of staff**</u><br><br>Programme theory 'seed' statements<br><br>**Department of Health. End of Life Care Strategy, 2008**<br><br>[M]any health and social care staff have had insufficient training in identifying those who are approaching the end of life, in communicating with them or in delivering optimal care. To address this, a major workforce development initiative is now needed, with particular emphasis on staff for whom end of life care is only one aspect of their work. (p. 12)<br><br>**House of Commons Health Committee report, 2015**<br><br>"Many of those who provided written evidence to this inquiry expressed the opinion that health care staff need support to develop their competence in identifying, providing care to and communicating with people at the end of life and their families and carers." (p. 20)<br><br>**See also the policy quotes on pages 2-5, in particular Q6 and Q8**.<br><br><u>**(A/O, M, C) Identification of end of life stage is much easier in cancer and, respectively, challenges of identification are more likely in other conditions**</u><br><br>Programme theory 'seed' statements<br><br>**Department of Health. End of Life Care Strategy, 2008**<br><br>"3.3 Some people with long term conditions remain in reasonably good health until shortly before their death, with a steep decline in |

| | | | |
|---|---|---|---|
| | | | the last few weeks or months of life. Others will experience a more gradual decline, interspersed with episodes of acute ill health from which they may, or may not, recover. A third group are very frail for months or years before death, with a steady progressive decline.<br><br>3.4 These three patterns, or trajectories, are illustrated in Figure 1 *[Note, the review team – not shown here]*. Some authors have suggested that the first pattern may be typical of cancer, the second may be typical for people with organ failure (e.g. those with heart failure or chronic obstructive pulmonary disease), and the third may be typical for people with dementia.<br><br>3.5 However, empirical evidence from a cohort of patients who died of a variety of conditions over a two year period indicates that the picture is more complex (Figure 2).” (pp. 45-7)<br><br>“The trajectories of different conditions do, however, vary. The predictability of prognosis towards the end of life is generally somewhat greater for cancer than for other conditions.” (p. 97)<br><br><br>**House of Commons Health Committee report, 2015**<br><br>“The British Medical Journal has described three distinct illness trajectories for people with progressive chronic illnesses:<br>• a trajectory with steady progression and usually a clear terminal phase; mostly cancer<br>• a trajectory with gradual decline, punctuated by episodes of acute deterioration and some recovery, with more sudden, seemingly unexpected death; for example, respiratory and heart failure<br>• and a trajectory with prolonged gradual decline; typical of frail elderly people or people with dementia.” (p. 13)<br><br>*Note, the review team: The BMJ paper referred above is the paper mentioned in the EoLC strategy (Murray S et al. 2008). This time,* |

| | | | |
|---|---|---|---|
| | | | *however, the added complexity indicated by the Strategy (which then points to Figure 2, representing much more complex data provided by M Gott) has been lost.*<br><br>"They [British Heart Foundation] note that GPs admit that introducing palliative care is fairly straightforward for people with cancer, who typically have a clear terminal decline, but much more difficult for patients with other life-threatening illnesses." (p. 15) |
| **1.4.**<br><br>**A, C, M, O** | **Timely identification works better in theory than in practice**<br><br>Prognostic judgements in end of life care (A) – the foundation for triggering end of life specific courses of action and services (M) –<br><br>are made through various combinations (M) of probabilistic objective criteria (M),<br>professional judgement (M), and/ or<br>subjective intuitions (M)<br><br>using a variety of prediction modalities and frameworks (A, M)<br>by health professionals<br>of different professional backgrounds (C),<br>of different levels of skills and experience (C),<br><br>in different phases of an illness or frailty (C)<br>in dynamic situations (C)<br>with different degree of input from other professionals (C)<br>and relative to dynamic patient presentations (C),<br><br>resulting in problematic prognostic accuracy (O). | White et al. 2016 ***<br><br>White et al. 2017 ***<br><br>Downar et al 2017 ***<br><br>Bluhm et al. 2016 **<br><br>McGaughey J 2017 * | **<u>(O) Overall accuracy of prognosis in EoLC</u>**<br><br>**White et al. 2016**<br><br>*(Setting/ participants) Palliative populations and settings; "real patients" rather than hypothetical cases; English language papers.*<br><br>*(Methods) Systematic review of 42 studies on predictions of survival in palliative patients, over 12,000 prognostic estimates. Databases: MEDLINE, Embase, CINAHL, and the Cochrane Database of Systematic Reviews and Trials from inception to 2015. Papers assessed for bias (QUIPS tool) but not excluded on the basis of it.*<br><br>Distribution of percentages of accuracy for categorical survival estimates (re-organisation of data from Fig 2 of White et al., original presentation by type of categories)<br><br>**% accuracy CI Estimates Categories Reference**<br><br>**< 30%**<br>23.3% 20.0-26.9 600 4 Llobera 2000<br>27.2% 21.7-33.2 243 8 Holmebakk 2011<br>27.3% 15.0-42.8 44 6 Glare 2001<br>27.6% 23.2-32.3 395 4 Fairchild 2014<br><br>**30%-39%** |

| | | | |
|---|---|---|---|
| | Such challenges around the accuracy of prognosis are further exacerbated by emotional factors (M), such as reluctance to share bad news (M);<br>perceptions of a preference, on the part of patients, to maintain hope (M);<br>or some health professionals' own resistance to "admitting failure" in not being able to do more for a patient (M). | | 30.2% 24.6-36.4 248 2 Shah 2006<br>31.7% 21.9-42.9 82 7 Stiel 2010<br>31.9% 22.7-42.3 94 2 Bruera 1992<br>32.0% 19.5-46.7 50 5 Kao 2011<br>33.3% 29.4-37.3 580 3 Gripp 2007<br>34.0% 31.8-36.2 1835 7 Hui 2011<br><br>**40%-49%**<br>41.0% 35.1-47.1 273 5 Zibelman 2014<br>45.0% 35.0-55.3 100 6 Glare 2004<br><br>**50-59%**<br>51.5% 44.9-58.1 233 3 Vigano 1999<br>55.6% 38.1-72.1 36 7 Selby 2011<br>55.8% 51.3-60.1 511 3 Brandt 2006<br>56.7% 49.5-63.6 203 3 Muers 1996<br>57.4% 54.3-60.6 987 3 Gwilliam 2013<br>57.6% 52.7-62.3 429 4 Fromme 2010<br><br>**Over 60%**<br>72.8% 66.9-78.2 254 7 Thomas 2009<br>78.4% 75.8-80.7 1128 2 Addington-Hall 1990<br><br>**Downar J et al. 2017**<br><br>*(Setting/ participants) Patients with cancer (5 studies), renal failure (7), end-stage heart disease, end-stage lung disease, heterogenous population with critical illness, a primary care practice population (1 study each). Median incidence of death for the cohorts 15.1%.*<br><br>*(Methods) Systematic review of 16 studies/ 17 cohorts (11 621 patients) on the Surprise Question ("Would I be surprised if this patient died in the next 12 months?"). Death at 6 to 18 months the outcome of interest. Eleven databases/ platforms searched. Overall risk of bias,* |

| | | | |
|---|---|---|---|
| | | | *as per Quality in Prognosis Studies tool: high in 4 studies, moderate in 10 studies and low in 2 studies.*<br><br>For death at 6 to 18 months, the pooled prognostic characteristics for the Surprise Question (SQ) were:<br><br>**67.0% sensitivity** (95% CI 55.7%–76.7%) – amongst those who died, those who were expected to die as per the Surprise Question;<br><br>**80.2% specificity** (73.3%–85.6%) – amongst those who survived, those who were expected to survive as per the Surprise Question;<br><br>**37.1% positive predictive value** (95% CI 30.2%–44.6%) – the proportion of patients who died when the clinician predicted dying;[8]<br><br>**93.1% negative predictive value** (95% CI 91.0%–94.8%) – the proportion of patients who survived when the clinician predicted survival.<br><br>"The surprise question performs poorly to modestly as a predictive tool for death, with worse performance in noncancer illness."<br><br>"Prognostic performance was worse for noncancer illness, missing more than one third of those who died and more than two-thirds of positive results proved to be false."<br><br>"Based on these findings, the SQ should not be used as a stand-alone prognostic tool, and we do not know whether it is more accurate for identifying patients with unmet palliative needs than it is for those in the final year of life." |

[8] Simple formulations of PPV and NVP taken from White et al. 2017

| | | | |
|---|---|---|---|
| | | | **White et al. 2017**<br><br>*(Setting/ population) Patients with end-stage renal disease (8 studies), cancer (6), heart failure (4), sepsis (1), COPD (1) and variety of diagnoses (6). Patients died within the specified timeframe on 4217 occasions (16%). Country: 10 studies UK; 9 US; Germany, Hong Kong, India, Italy, Japan, New Zealand, Spain (one study each). English language publications only.*<br><br>*(Methods) Systematic review of 26 papers on the Surprise Question, relevant data extracted from 22 papers. 25,718 estimates. Eight databases (no retrieval from one). Quality assessed with the Newcastle-Ottawa scale; papers not excluded but appraisals used in sensitivity analysis.*<br><br>Wide variation in the reported accuracy of the SQ:<br><br>**11.6%** to **95.6%** – range for **sensitivity**<br>**13.8%** to **98.2%** – range for **specificity**<br>**13.9%** to **78.6%** – range for **positive predictive value**<br>**61.3%** to **99%** – range for **negative predictive value**<br><br>*Note, the review team: The reviews of Downar et al. 2017 and White et al. 2017 on the surprise question appear to be targeting the same papers, although one frames the review around predicting death and the other around identifying end of life patients. There are more papers and predictions in the review of White et al. 2017. Further work is needed to assess the degree of overlap in their data sources, but both reviews identify suboptimal and also variable performance of the SQ.*<br><br>*Note, the review team: Sources of data which can be used to assess the accuracy of predictions at a very local level include:*<br>- *evaluations of anticipatory prescribing practices;*<br>- *data from Electronic Palliative Care Coordination Systems* |

| | | | |
|---|---|---|---|
| | | | <u>**(C) Professional background, impact**</u><br><br>**White et al. 2016**<br><br>*See above for brief overview of methods and setting/ participants (first White et al. reference)*<br><br>No consistent evidence that one professional group or sub-group of clinicians was any more accurate than any other profession or sub-group.<br><br><u>**(C) Experience, impact**</u><br><br>**White et al. 2016**<br><br>Level of experience as improving accuracy – mixed evidence (confirmed in 2 studies, refuted 3).<br><br><u>**(C) Multidisciplinary team working, impact**</u><br><br>**White et al. 2016**<br><br>Accuracy may be better when the prognosis is made by a multidisciplinary team (2 studies).<br><br><u>**(C) Proximity to death, impact**</u><br><br>**White et al. 2016**<br><br>Time frame of the prognosis (e.g. imminent death vs. within 12 months) appeared to affect both the accuracy overall and the relative accuracy of different professionals (2 studies).<br><br>**White et al. 2017** |

| | | | |
|---|---|---|---|
| | | | "The meta-regression indicated that the increase in time frame did not impact on the diagnostic accuracy of the SQ: comparing up to 30 days with 12 months (difference in accuracy = 0.8%, 95% CI –12.8 to 14.5, P = 0.901) and comparing up to 6 months with 12 months (difference = 4.3%, 95% CI –10.8 to 19.4, P = 0.561)."<br><br><br>**<u>(C + C) Professional background and proximity to death</u>**<br><br>**White et al. 2016**<br><br>Nurses and healthcare assistants may be better at recognising imminent death than other professionals (2 studies).<br><br><br>**<u>(A, M) Type of estimate, impact</u>**<br><br>**White et al. 2016**<br><br>Probabilistic estimates (4 papers) may be slightly more accurate than categorical or continuous temporal estimates of survival.<br><br>*For evidence on professionals' emotions around sharing a negative prognosis (Bluhm et al. 2016), see 2.6.1. below.* |
| **1.5.**<br><br>**C, M, O** | **Ignoring the observations of low-level staff**<br><br>Some staff who provide hands-on care (C) to patients may make highly accurate observations of less conspicuous changes to their condition (M), allowing them to predict a transition towards the end of life (O). However, their input is often ignored (M) and opportunities for care "switching tracks" quickly into end of life care are missed (O), as they are considered low-level personnel without the training and skills | Sims-Gould et al. 2010 BROADER (Canada) | **<u>(M, O) Observations of patient condition made by low-level staff ignored by more senior staff</u>**<br><br>**Sims-Gould et al. 2010**<br><br>*(Setting/ participants) Ontario, Canada, 110-bed long-term faith-based, not-for-profit care home; 18 deaths or residents with dementia in the previous year; experiences of dying and end of life care for patients with dementia of personal support workers and non-clinical workers. Personal support workers (PSWs, also called 'care aides')* |

| | | | |
|---|---|---|---|
| | needed for such judgements (M). Time pressures for more senior staff (C) exacerbate the tendency to ignore what appears unimportant/ unreliable as information (M). | | *deliver the majority of hands-on resident care; non-clinical workers here include maintenance and support staff.*<br><br>*(Methods) Part of a broader study using "focused ethnography". In this paper – data from 44 personal support workers (17 interviews and focus groups, unclear how many of which), and 4 non-clinical workers (4 interviews). Thematic analysis of data by three researchers, both independent work and team discussions. Findings presented to staff for validation.*<br><br>*"It is frustrating, and I know I've had an experience of people that are deteriorating really, really quickly, and [the nurses] are still telling you, 'No, get them up, get them in the dining room,' and you're...in tears."*<br><br>*"Yeah, you are just bottom of the barrel. What do you know about that person, you know? Even just day-to-day care, if you go and tell an RN something is different about somebody — 'Oh, they were like that a month ago. That's happened before.'"* (p. 127)<br><br>**(C) Time constraints exacerbate the tendency**<br><br>"Time constraints often fracture communication or create a feeling that one's voice or perspective is not valid or valued by others." (p. 127) |
| **1.6.**<br><br>**C, M, O** | **Awareness of long-standing needs and PEoLC**<br><br>Some terminally ill patients (cancer diagnosis) (C), whose needs for symptom control and help with self-care have been relatively long-standing (C), may be less likely to receive input from community specialist palliative nurses in the last year of life (A/O), possibly | Addington-Hall and Altmann 2000 | **(C, O) Association between longer-standing health needs and provision of PEoLC care**<br><br>**Addington-Hall and Altmann 2000**<br>*(Setting/ participants) England, 20 district health authorities (self-selected but nationally representative); community specialist palliative nurses (Macmillan, hospice at home, others); by now, old data (deaths in last quarter of 1990).* |

| | | | |
|---|---|---|---|
| | due to being perceived as chronic disease patients rather than PEoLC patients (M).<br><br>Early identification of terminal phase (A) may thus, paradoxically, be associated with reduced likelihood of being prioritised for community PEoLC (M/O). | | *(Methods) Random sampling of 270 deaths (through death certificates) per health district, though cancer deaths sampled disproportionately due to interest of health districts (54%); semi-structured interviews with bereaved carers about the last 12 months of life; 71% response rate for cancer deaths (2074/2915).*<br><br>"Patients who had had symptoms for more than 6 months were less likely to receive care from these nurses"<br><br>In logistic regression, being dependent on others for help with self-care for more than 1 year (along with four variables representing types of cancer) was associated with a decreased likelihood of receiving community specialist palliative care.<br><br>**(M) Perception of patients as chronic disease rather than palliative**<br><br>**Addington-Hall and Altmann 2000**<br><br>Authors' explanation of pattern of data<br><br>"Patients who had had symptoms for more than 6 months were less likely to receive care from these nurses, suggesting that these patients may have been seen as falling within the rubric of chronic disease care rather than palliative care." |
| **1.7.**<br><br>**C, M, O** | **The uncertainty of predictions may be "reverse engineered" to secure better care for patients**<br><br>In the context of service capacity limitations (C), a commitment to do one's best for a patient (M/C) and/or active seeking of help by the patient family (M/C), health professionals may "reverse engineer" the uncertainty of predictions at the end of life (M) | Lucas et al. 2008 | **(M, O) Revising prognosis so that it fits referral criteria to enable a patient to receive enhanced services**<br><br>**Lucas et al. 2008**<br><br>*(Setting/ participants) Bradford, UK (large and deprived borough); hospice at home team covering 3 areas with a combined population of 386,000. Service targeted at patients with a prognosis of 6 weeks or* |

| | | | |
|---|---|---|---|
| | and refer a patient on the basis of a "reconsidered" (but, potentially, no less accurate) prognosis (M). Patients are thus enabled to access the services that would meet their needs (O), but which were out of bounds due to their prognosis-focused referral criteria (M). | | *less, irrespective of diagnosis. Practical nursing support, supplementing that provided by the patient's own nursing team. Team composition: 1 Sister, 2 Staff Nurses, 6 Marie Curie trained Health Care Assistants, and Agency staff. Service context of high staff turnover, considerable work pressure and absence of adequate admin support.*<br><br>*(Methods) Data collected from Jul 2001 (inception of service) to Jun 2006. Postal questionnaire used as part of clinical audit. Respondents: carers ($N_{eligible}$ = 570, $n_{sample}$ = 289, response rate 50.7%), district nurses (n = 508, 89% response rate, no reminders) and GP's (n = 444, 78%, no reminders). 15 questions for carers; 12 for DNs and 3 for GPs. Ample free-text comments, even if questionnaire did not afford it (e.g. extra pages added, written in margins). Qual data analysed using grounded theory techniques.*<br><br>*"*Anecdotally, we identified that in some cases GPs were referring people with non-cancer diagnosis and claiming a prognosis of six weeks or less in order to give their patients access to the enhanced services hospice at home could offer. In effect the inexact science of prognosis is being used in two ways – one to exclude some people and one to include them in this sought after service even when GP's may not have an evidence based judgement to reassure all parties that the six week prognosis was realistic. In effect the six week rule was manipulated to maximise care for individual patients." (p. 11) |

| TIMELY REFERRAL<br>to community PEoLC services, following or not from short prognosis/ identification of EoLC stage | | | |
|---|---|---|---|
| **2.1.**<br><br>**rough**<br>**A, C, M, O**<br>**A, C, M, O (functional equivalent, negative framing)** | **Benefits of timely (generally earlier) referral to palliative and end of life care services in the community**<br><br>Timely referral to (community) PEoLC services (A) allows sufficient time for evaluating and organising care around (M) the patient and family needs, wishes, preferences and life circumstances, e.g. suitability of home for home care (M, C),<br>and also relative to the capacity and timelines of local services (C).<br><br>This enables the best possible outcomes for the patient, their family, and the sustainability of the health system (e.g. by avoiding unnecessary admissions and interventions). It also makes it more likely that the patient dies at home, if this has been their wish ($O_{multiple}$).<br><br>In contrast, late referrals ($A_{fail}$) may preclude opportunities (M) for<br>the adequate and comprehensive assessment of needs (M),<br>eliciting a patient's wishes and preferences (M),<br>arranging care in view of the above (M),<br>as well as<br>for the patient, family and generalist staff to benefit from highly effective, fine-tuned, flexible decisions and courses of action (M) around reducing pain and suffering and increasing quality of life (O), enabled by | Fellowes et al. 2003 (trigger)<br><br>Mintzer and Zagrabbe 2007 * BROADER (United States) (trigger)<br><br>National Institute for Health and Care Excellence 2019<br><br>Department of Health 2008<br><br>Domain knowledge of team | **(A, O, C) Timely, which is generally earlier, referral improves outcomes for cancer patients, but is unclear if this is the case for non-cancer patients**<br><br>Programme theory 'seed' statement<br><br>**National Institute for Health and Care Excellence guideline for service delivery, 2019**<br><br>"There is a body of research into the optimal timing of referral to specialist palliative care in cancer patients, which generally points to earlier referral leading to better patient-reported outcomes. The committee noted that similar evidence is very limited for patients with a non-cancer diagnosis, for example in patients with progressive organ failure, such as advanced heart failure, or patients with life-limiting neurological disease, such as motor neurone disease or dementia. Such patients are typically referred very late to specialist palliative care, if at all. Further research would compare outcomes for people having a combination of early identification and specialist palliative care input with those for people having usual care." (p. 19)<br><br>**(A, O) Late referrals as a source of frustration for specialists**<br><br>Framing sections evidence/ statement<br><br>**Mintzer and Zagrabbe 2007** |

| | | | |
|---|---|---|---|
| | the greater knowledge, skills and experience of specialist staff (M/C).<br><br>This may mean that patients endure (long) periods of preventable pain and suffering, are move vulnerable to unnecessary admissions, and/or do not have their end of life care wishes met ($O_{multiple}$).<br><br>Families and other informal carers may also not receive the support they need (M/O) and, as a result, struggle with providing care (M/O), experience significant deterioration of their own health and well-being (M/O), and not be able to cope with crises in home care (M), resulting in unnecessary hospital admissions (O). After the death, they may experience feelings of guilt and complicated grief (O) and, potentially, initiate complaints against the health services (O), which had let them down (M).<br><br>Late referrals ($A_{fail}$) also result in frustration for palliative and end of life care professionals (O). This is because opportunities for providing optimal care have been missed (M). Also, with the often short timelines in end of life care and often limited capacity of PEoLC services (C), specialists have not been given the chance to respond in the ways they would have wanted or were expected of them (M). This can create/ contribute to interprofessional tensions and mistrust (O/M/C).<br><br>There is, however, uncertainty whether earlier referrals, which are generally associated with better patient-reported outcomes in cancer, are similarly recommended for patients with non-cancer conditions. (C) | | "Often, hospice is called in very late, within the last week or two of life—sometimes the last day or two—when optimal support and preparation for death cannot be provided. Such late referrals are frustrating to practitioners who deal with end-of-life care."<br><br>**(A, O) Late or no referrals as a source of complaints against the health system**<br><br>**Department of Health. End of Life Care Strategy, 2008**<br><br>"Its [of the Healthcare Commission] 'Spotlight on Complaints' report assessed just over a total of 16,000 complaints made about NHS organisations between July 2004 and July 2006. Approximately half of these related to care given in acute hospitals. Of these, no less than 54% related in some way to end of life care. In its latest report 'Spotlight on Complaints 2' (April 2008) the Commission examined 50 cases where the primary complaint was about end of life care. These complaints were mainly about poor communication, lack of basic comfort, privacy and psychological care and late or no referral for specialist palliative care. Relatives frequently commented that they seemed to be the first to recognise that the patient was dying. Inappropriate invasive procedures were often undertaken, even in the dying phase." (pp. 24-25)<br><br>*Note, the review team:*<br><br>*The rough theory we have described to the left includes formulations based on the background knowledge of the team which have not yet been linked to a sufficient number of illustrative sources (contents of this column). "Timely/ early vs. late referrals" at this high level of generality was de-prioritised as a potential focus for further targeted searches, as it was expected to follow a similar logic to that around timely/ early and delayed identification (1.1. and 1.2. above).* |

| | | | |
|---|---|---|---|
| | | | *We thus focused the articulation of CMOs and associated evidence on more specific issues, as represented from 2.2. to 2.10. below.* |
| **2.2.**<br><br>**C, M, O** | **Unclear referral criteria and/or limited awareness of them**<br><br>Even if generalist staff have sufficient awareness of local community PEoLC services and value them (M/C), their referral patterns may still be suboptimal (O) due to uncertainty about appropriate referral times and associated services (M).<br><br>*Hypothesis (M), this review: Perceptions of lack of clarity of a criterion/ set of criteria often relate to the need to interpret whether a particular case meets a criterion/ set of criteria or not. In this sense the problem may be in the grey area of whether something counts as meeting a criterion (as would be, for instance, "end of life stage") rather than in the lack of clear articulation and communication of relevant criteria. The solution then is not so much in clarifying the referral criteria, but in exploring the potentially sizeable grey zones of non-exemplary cases.* | Fellowes et al. 2003 | **(M) Uncertainty and confusion about appropriate referral times**<br><br>**Fellowes et al. 2003**<br><br>*(Setting/ participants) UK, Marie Curie Nursing Service*<br><br>*(Methods) Study-specific questionnaire to District Nurses in 37 community trusts (4 randomly selected, stratified for urban/ rural, per each of the 10 Marie Curie Nursing Service districts in the UK). Questionnaires sent randomly to 40 or all (if less) DNs within a participating trust. 879 out of 1,379 surveys (64% response rate). A significant number of missing responses to some questions, but also almost 250 DNs provided additional, free-text comments.*<br><br>"The MCNS [Marie Curie Nursing Service] is valued, but confusion exists about appropriate referral times and the services provided."<br><br>**Fellowes et al. 2003**<br><br>Framing sections evidence/ statement<br><br>"However, the study also found that other health care professionals were uncertain about when they should refer people to the service. The study also found a perception within the MCNS that some referrals were inappropriate (Higginson and Wilkinson, 2002)."<br><br>*Note, the review team: We do not see direct evidence in Higginson and Wilkinson 2002 to justify the interpretation of Fellows et al. but leave their claim and our challenge for further critical assessment.* |

| | | | |
|---|---|---|---|
| **2.3.**<br><br>**M, O** | **Potential over-confidence in own knowledge of generalist staff**<br><br>Generalist staff may perceive their PEoLC knowledge and ability to deal optimally with dying patients as better than they are (M), resulting in fewer or later referrals to specialist services than beneficial for patients (O). | Fellowes et al. 2003 | **(M) Potential discrepancy between actual and perceived PEoLC knowledge of a significant proportion of generalist staff**<br><br>**Fellowes et al. 2003**<br><br>*(Setting/ participants) UK, Marie Curie Nursing Service*<br>*(Methods, brief) Questionnaire completed by 879 District Nurses (64%).*<br>*See 2.2. for details*<br><br>"It is surprising that almost 70 per cent of district nurses felt that they had sufficient expertise to provide adequate palliative care but only 13 per cent had attained a recognised palliative care qualification."<br><br>Top reason for not referring: "able to give this patient appropriate palliative care myself", selected "often" by 68.7% of respondents (820 responses to question). |
| 2.3.1.<br><br>**A, M, O (tentative hypothesis)** | **Unintended consequences of brief PEoLC training events for generalist staff?**<br><br>Brief PEoLC training events for generalist staff (A) may raise *confidence* in own knowledge and skills more than they raise *knowledge and skills* (M/O). The intended outcome is that generalists are better able to look after PEoLC patients by themselves (O). This may, however, be accompanied, and potentially counterbalanced, by an unintended outcome that generalists refer to specialist care less than would be beneficial for patients (O). | Fellowes et al. 2003 (trigger) | (A, M, O) Brief PEoLC training events for generalists may have unintended outcomes on specialist referrals<br><br>This review, potential explanation for pattern of data; requires further evidence<br><br>*Fellowes et al. 2003 discuss the issue that almost 70% of respondents felt they had sufficient expertise to provide palliative care while only 13% had a recognised qualification in it (see immediately above). The authors, however, do not link this configuration to a further finding on training, namely that 91.8% of respondents had attended palliative or terminal care study days.*<br><br>*Could it be that brief training initiatives raise confidence more than they improve knowledge and skills, resulting in decisions not to refer when this would have been the best course of action? If this is, indeed, an unintended consequence observed in some cases, is its occurrence* |

| | | | |
|---|---|---|---|
| | | | *affected by characteristics of the specialist teams (who may also be providing the training)?*<br><br>*Deprioritised for further searches.* |
| **2.4.**<br><br>**C, M, O** | **Family and informal carer needs as a primary reason for referring to specialist services and for the timing of the referral**<br><br>The needs of the family and informal carers may be the primary trigger for referring to specialist PEoLC community services (M). While this is an appropriate referral trigger (A), the referral might nonetheless be considered delayed (O) by specialists if the needs of the dying patient, e.g. of symptom control, are assessed by them as having required earlier expert involvement (M).<br><br>At the same time, some families may have declined earlier offers of involvement of specialist PEoLC community services (M/C), constraining the referral choices of generalist staff (O). | Fellowes et al. 2003 | **(M) Family and carer needs as the primary trigger for referrals to specialist PEoLC community services**<br><br>**Fellowes et al. 2003**<br><br>*(Setting/ participants) UK, Marie Curie Nursing Service*<br>*(Methods, brief) Questionnaire completed by 879 District Nurses (64%).*<br>*See 2.2. for details*<br><br>*Reasons prompting referral with medium to high likelihood (reason, %, number of respondents to question)*<br><br>Factors impacting on family or carers — 88.1% (840)<br>Anxiety/ other psychological problems — 64.8% (810)<br>Practical problems — 62.8% (814)<br>Social problems — 59.9% (819)<br>Other physical problems — 57.1% (798)<br>Pain — 42.6% (829)<br>Communication problems — 34.0% (801)<br><br>*Factors that influence "often" the timing of referral*<br><br>Needs of family/carer — 96.5% (868)<br>Stage of disease — 86.8% (838)<br>Timing of referral of patient to me — 34.3% (796)<br>Workload of local Marie Curie nurses — 10.6% (802) |

| | | | |
|---|---|---|---|
| | | | My workload 3.3% (819)<br><br>**<u>(C) Trajectory of family preferences of patient referral</u>**<br><br>**Fellowes et al. 2003**<br><br>"[A] referral at a late stage was often at the wishes of the family who did not want MCNS input earlier."<br><br>"[A] significant number of district nurses stated that the patient's and his or her family's unwillingness to have the service was a reason for non-referral." |
| **2.5.**<br><br>**A, C, M, O** | **Terminal stage as the other primary reason for referring to specialist services**<br><br>While PEoLC community services may be targeted at patients with a broad range of needs – e.g. pain relief and symptom control while curative treatments are still ongoing, all palliative care stages, the whole last year of life, etc. (A/C), generalists may consider such services seriously only in the last few months, weeks or even days of life (M/C), potentially prompted by the growing needs for support of families and carers (M/C, see 2.4). As a result, referrals occur most often in terminal stages (O). | Fellowes et al. 2003 | **<u>(O) Referrals as occurring most often in terminal stages</u>**<br><br>**Fellowes et al. 2003** (details in 2.2; raw numbers below are for number of valid responses to a particular question)<br><br>*Timing of referral relative to disease stage/ proximity to death (responses 'often')*<br><br>Soon after diagnosis of incurable illness 8.6% (799)<br>When palliative care needed 47.7% (819)<br>When terminal care needed 88.3% (849)<br>When end-stage care needed 94.6% (857)<br><br>**<u>(C) Service specifications are for a far broader range of needs and a lengthier timeline</u>**<br><br>**Fellowes et al. 2003** |

| | | | |
|---|---|---|---|
| | | | "MCNS state that the service is available for any patient with a palliative care need (Box 1), yet less than half of district nurses (47.7 per cent) said they would often consider referring patients during the palliative phase."<br><br>**<u>(C, M, O) Interaction between terminal stage and family needs in decisions for referral</u>**<br><br>**Fellowes et al., 2003**<br><br>Authors' explanation for pattern of data<br><br>"The impression given is that consideration for referral is focused on the terminal and end stages of illness. This in itself is not surprising as these are the times when patient dependency and the support needs of the carers are at their highest." |
| **2.6.**<br><br>**C, M, O** | **Impact of the increasing availability of curative therapies**<br><br>The increasing availability of curative therapies (C), and of oral preparations in particular (C), contributes to later and later referrals (O), as there is almost always a further line of therapy that can be tried (M). At the same time, only a small proportion of patients respond to such therapies (O), the gains are minimal (O), and many patients die without receiving any, or adequate, palliative care (O). | Mintzer and Zagrabbe 2007 * BROADER (US)<br><br>National Cancer Institute 2020 BROADER (US) | **<u>(C, M, O) Increased availability of curative therapies leads to curative attempts closer and closer to the end of life. Palliative care is, as a result, delayed while the effectiveness of therapies given as second-line or beyond is limited</u>**<br><br>**Mintzer and Zagrabbe 2007**<br><br>*(Setting/ participants) United States, oncology-hospice interface, Medicare*<br><br>*(Methods) Review of the literature on antineoplastic agents from the past 10 years; review of subsample in detail – pivotal trials on newer agents in non-small cell lung cancer for effectiveness as second-line* |

| | | | |
|---|---|---|---|
| | | | *therapy and beyond; review of the records of 50 patients with breast, lung and colon cancer.*<br><br>Authors' explanation of pattern of observations/data<br><br>An "increasingly significant cause of delayed referral: the proliferation of antineoplastic agents that are active, but only in a few of the patients who are treated".<br><br>The authors suggest that "treatment closer to the end of life is an inevitability that is likely to increase with the increasing number of agents available". They see the increased availability of oral preparations as likely to further the tendency.<br><br>At the same time only a small percentage of patients respond to therapies given as second-line or beyond (while it takes 6-12 weeks to assess response) (pp. 128-129)<br><br>**(M/O) Curative care attempted closer to death**<br><br>**Mintzer and Zagrabbe 2007**<br><br>Evidence from the review of records (50): "a median interval from the date of the last regimen given to date of death of 30 days, with a range of 3 days to 8 months" (p. 128)<br><br>**(C) Increased availability of curative therapies**<br><br>**Mintzer and Zagrabbe 2007** |

| | | | |
|---|---|---|---|
| | | | Circa 2007 (year of publication), Mintzer and Zagrabbe identify 26 agents which were approved by the FDA in the preceding decade and have come to be used routinely for the treatment of a variety of malignancies.<br><br>**National Cancer Institute 2020**<br><br>As of Feb 2020, the A to Z list of cancer drugs of the National Cancer Institute (US) lists **561** approved drugs approved for cancer or conditions related to cancer (https://www.cancer.gov/about-cancer/treatment/drugs). Update, Aug 2021: the number has increased to **641**.<br><br>**(O) Limited effectiveness of therapies given as second-line or beyond**<br><br>*Note, the review team: This category of relevant evidence was prompted by actual evidence in Mintzer and Zagrabbe 2007. However, as it came from only 3 trials and the paper is now approaching 15 years since data collection, we chose to indicate it as a necessary type of evidence but not include the data.* |
| **2.6.1.**<br><br>**A, C, M, O** | **Non-palliative professionals as responding to patient wishes for further curative attempts, in the context of ever-present uncertainty and need for hope, rather than their clinical judgement**<br><br>Non-palliative health professionals may be judged as being overly aggressive in treatments at the end of life (M/C); resistant to palliative care (M/C); overly committed to a curative ethos (M/C); driven by a mindset of having to do something (M/C), even by a hubris that much more is within their power at the life and death line than it actually is (M/C), and | Mintzer and Zagrabbe 2007 * BROADER (US)<br><br>Bluhm et al. 2016 ** BROADER (US) | **(C, M, O) Attempts at curative treatment are frequently made following the desires of patients and families rather than an oncologist's best judgement, yet are still meaningful in the context of ever-present uncertainty and maintaining hope in those who need it**<br><br>**Mintzer and Zagrabbe 2007**<br><br>Anecdotal evidence from personal experience that it is often patients and families that desire to try out other treatments, even when these are not recommended by the oncologist. |

| | | | |
|---|---|---|---|
| | perceiving death as a failure (M/C). As a result, patients are subjected to unnecessary interventions (A/O), which prolong suffering rather than life (O).<br><br>While this may be a valid line of explanation in some cases, it interacts in complex ways with a far less judgemental one:<br><br>A key driver of decisions to continue with curative treatments (A) are often the wishes of the patient and their family (M) rather than a non-palliative professional's clinical judgement (M). At the same time, following the former is a meaningful choice in a context of increasing treatment options (C), an awareness that there is always the off-change, the 1% uncertainty, the miracle even (C), and the value of hope till the very end for some patients and families (C). Persevering with a curative course of action very close to the end of life may be further supported by a non-palliative professional's own difficult emotions (M/C). These are often easier to manage by "doing something" and responding to the patient wishes and preferences for trying once more than by accepting that the patient is dying (M). | | **Bluhm et al. 2016**<br><br>*(Setting/ participants) US Midwest; oncologists from three types of settings: academic, private practice, and an oncology fellowship program. All participants routinely prescribed cytotoxic chemotherapy.*<br><br>*(Methods) Recruitment through physicians and administrators known to study team. Semi-structured face-to-face interviews with 17 oncologists. Qualitative content analysis. Reading and rereading of 3 transcripts by 2 team members, coding scheme developed on the basis of them. Line-by-line coding of the same transcripts by both team members; discussion; independent coding of transcripts 4-6; final coding scheme agreed, analysis completed by one team member.*<br><br>Interview quotes (paper does not give indication of source, e.g. interview number):<br><br>*"Usually I think if physicians end up treating to the death bed, which we sometimes do, it's because the family or the patient [is] very insistent."*<br><br>*"And so when the patient and their family [are] saying 'Well, is there anything else,' you sort of feel like who are you to close that door, [be]cause you actually don't know with 100% certainty."*<br><br>*"He's in the ICU, he's actively dying, and he's telling us, 'Please don't stop the chemotherapy.' You know what I mean? So his fight, that's what made him happy. I think if we hadn't treated him, we'd have felt better, but we might have kind of killed him in a way then, too."*<br><br>*"I know a colleague who treats to the grave, and I think he tries to have these conversations with patients about end of life, but he always wants to present some hope, [be]cause you do. You want to balance reality with hope, and I think in his case, it always comes out* |

| | | | |
|---|---|---|---|
| | | | *in such a way that people choose the hope side and go home with a prescription."*<br><br>**(M/C) Perceptions of oncologists as overly aggressive in their attempts for curative treatment even when palliation would have been the far better option**<br><br>**Mintzer and Zagrabbe 2007**<br><br>(in the context of discussing the proliferation of new therapies and the above anecdotal evidence) "Some have perceived oncologists as being overly aggressive with treatment. Often the use of chemotherapy or newer targeted therapies that, in retrospect, have been given close to the end of life is viewed as an error in judgment." (128)<br><br>**(M/C) Curative and "do something" ethos amongst oncologists**<br><br>**Bluhm et al. 2016**<br><br>*"I didn't go into medicine to help people die."*<br><br>*"That's not just hard for them [patients] to handle, that's hard for us to handle. We see that every day. We don't want to tell somebody we can't do anything for them. So it's not just what they expect but also what we expect and maybe not being able to deal with the shortcomings of our practice … the bottom line is, you feel bad when you don't have anything to offer someone."*<br><br>*"Sometimes there is this urge like you have to offer something [be]cause you're called to be the oncologist."* |

| | | | |
|---|---|---|---|
| | | | *"That's how we think. That's how oncologists are trained to think. They have to offer people something."*<br><br>**(M/C) Oncologists' challenge of managing their own difficult emotions**<br><br>**Bluhm et al. 2016**<br><br>"Clinical factors take priority in determining late chemotherapy decisions when clear treatment choices exist. When clinical factors are ambiguous, emotion becomes a highly salient influence. Oncologists view late chemotherapy to be patient driven and use it to palliate emotional distress and maintain patient hope even when physical benefit is unexpected. Oncologists experience unique and difficult challenges when caring for dying patients, including emotionally draining communication, overwhelming responsibility for life/death, limitations of oncology to heal, and prognostic uncertainty. These challenges are also eased by offering late chemotherapy."<br><br>*"Before I walk in the door and I know I'm going to do this, I still take a deep breath and walk in. And when I leave I still say my own little things in my head to myself, even when the shtick gets rote…. Believe me, there are times where I feel like I'm having an out-of-body experience because the things I'm saying are coming out so routinely that I almost feel like I'm listening to myself or watching myself. And seeing people's reactions when you do that is emotionally draining no matter how many times you've done it, okay? It still is."*<br><br>*"I see those [test results] and my stomach just turns, you know. It's just so wrenching."*<br>*"It's pretty emotional, it's pretty intense in the room. You can kind of feel the heaviness, and it almost feels like every time you do this, a ton of bricks has fallen on top of you, if you let yourself go there."* |

| | | | |
|---|---|---|---|
| | | | **<u>(M/C) The burden of what some oncologists perceive as ultimately their decision and responsibility of deciding when to “let” a patient die</u>**<br><br>**Bluhm et al. 2016**<br><br>*“What depresses me is not that patients die. I know that. It is how I have to actively make the decision about when I’m going to let that happen. That’s what gets so hard, year after year, after year, is being, in a way, like God. It’s like looking at somebody you’re taking care of for awhile, a long time sometimes, and thinking to yourself, you know what, it’s just time for this guy to die. And that’s a hard decision [be]cause basically it’s my decision.”*<br><br>**<u>(M/C) The background awareness that there is uncertainty; that the off-chance, 1%, “miracle” at the end of life has happened before and could happen again</u>**<br><br>**Bluhm et al. 2016**<br><br>*“[I]f the crystal ball is telling me this guy is going to die in a week no matter what you do, you don’t treat him. Period. But it’s just not always that clear cut.”*<br><br>*“I wish I had been able to say, “Let’s not pursue chemotherapy,” but at the same time, it was just that, what if? What if, what if, what if?”*<br><br>*“Sometimes while people are at death’s door they get better. Maybe 1% are going to be cured and 99% will be dead in a month, but you don’t have a way of knowing that. Would you withhold it if the patient wanted it?”* |

| | | | |
|---|---|---|---|
| | | | *"You hear the attending physicians talk about that one person who really wanted treatment and they thought, there's no way they're ever gonna benefit from this. And sure enough, their disease responds and they have control, and the lung cancer patient that you thought was gonna be dead in 2 months is now alive at 2 years. I mean they're not many here. You can count [th]em on one hand. But there's always that story."* |
| **2.6.2.**<br><br>**C, M, O** | **Discipline-specific levels of awareness of treatment options**<br><br>Palliative care specialists (C) will be, typically, less aware (M) of innovative curative therapies in comparison to colleagues in the respective branches of medicine with curative goals (C). This too may result in conflict and antagonism about appropriate practices and timelines for PEoLC referrals (O/M/C). | Mintzer and Zagrabbe 2007 * BROADER (US) | **(M, C, O) Different levels of awareness of new curative options associated with a health professional's specialty, also leading to interprofessional tensions**<br><br>**Mintzer and Zagrabbe, 2007**<br><br>"Although those working in palliative care may be somewhat aware of these advances, palliative care professionals may not be aware of how many new agents have recently become available nor of the implications of these newer therapies in delaying hospice referrals."<br><br>"Failure to recognize this phenomenon will also result in increased frustration and sometimes antagonism between oncologists and palliative care/hospice providers." |
| **2.7.**<br><br>**C, M, O** | **System-level factors affecting late referral or non-referral**<br><br>Referral decisions ($O_{intermediate}$) may be affected by a range of factors associated with the structure and functioning of the health service and its entities (M/ C): | Fellowes et al. 2003 | **(C, M) Contribution of 'other' factors to referral decisions and their timing**<br><br>**(M/C) Timing of preceding referral**<br><br>**Fellowes et al. 2003 (also in 2.2 and 2.4.)**<br><br>*(Setting/ participants) UK, Marie Curie Nursing Service* |

| | | | |
|---|---|---|---|
| | ○ referral of patient to referring health professional in the first place (or patient help seeking) (M/C);<br>○ own workload (M/C);<br>○ perceptions of workload of specialist community services (M/C);<br>○ lack of confidence in, or even negative perceptions of, a particular PEoLC service (M/C);<br>○ accounting for service limitations (actual or perceived) (M/C);<br>○ concerns of overspending (M/C);<br>○ technical problems with the referral system (M/C);<br>○ the level of detail required at referral (M);<br>○ the use of alternative services (M/C);<br>○ the need to create a complete package of care, which means that any element in this package may, in some cases, be 'called upon' only if other elements are also becoming available (M/C).<br><br>While such factors are unlikely to affect decisions in cases where referral is clearly indicated (C), they may have decision-changing power in cases where the need for and benefit of the referral is less certain (C). Their accumulation may also reverse-engineer (M) perceptions of referral urgency (O $_{intermediate}$).<br><br>The incorporation of such factors in one's decision making (M) is likely to vary across health professionals (C), resulting in different referral | | *(Methods) Study-specific questionnaire to District Nurses in 37 community trusts (4 randomly selected, stratified for urban/ rural, per each of the 10 Marie Curie Nursing Service districts in the UK). Questionnaires sent randomly to 40 or all (if less) DNs within a participating trust. 879 out of 1,379 surveys (64% response rate). A significant number of missing responses to some questions, but also almost 250 DNs provided additional, free-text comments.*<br><br>34.3% of responding DNs state the time a patient has been referred to them influences their timing of referral.<br><br>11.8% state that referrals are often delayed or prevented as a patient has been referred too late to them to refer onwards.<br><br>**(M/C) Experiences of own and perceptions of others' workload**<br><br>**Fellowes et al. 2003**<br><br>10.6% state that workload of local Marie Curie nurses influences their timing of referral, while 3.3% state this of their own workload.<br><br>**(M/C) Lack of confidence in PEoLC services**<br><br>**Fellowes et al. 2003**<br><br>7.6% of respondents "indicated that late referral or non-referral of patients was due to the belief that the local MCNS could not provide appropriate care"<br><br>(explanation for pattern of data) "The relatively small proportion of nurses who indicated they would refer patients with pain and communication problems seems to indicate a lack of confidence in the skills and knowledge of the MCNS." |

| | | | |
|---|---|---|---|
| | patterns (O $_{intermediate}$) across individuals and teams (C).<br><br>Importantly, overall high positive perceptions of a service (C) do *not* 'switch off' factors which delay or limit (M) referrals (O $_{intermediate}$). | | "There may also be a lack of awareness that all grades of MCNS staff are expected to complete a recognised qualification in palliative care on appointment and to attend regular updates."<br><br>**(M/C) Concerns about overspending**<br><br>**Fellowes et al. 2003**<br><br>7.0% of respondents express concern that they would overspend on Marie Curie nurses.<br><br>"A minority of district nurses had only limited use of the MCNS, stating that … [they] were restricted in the frequency of referral for financial reasons."<br><br>**(M) Challenges of using referral system and processes**<br><br>**Fellowes et al. 2003**<br><br>9.5% of respondents reported frequent delays or obstruction of referrals due to booking system.<br><br>"Some district nurses attributed unavailability to both a shortage of nurses and to NurseLink, which they perceived as distant and inflexible in dealing with local needs."<br><br>"A small number of respondents commented on other aspects of communication, such as … the extent of details required at referral."<br><br>**(M/C) Referral to other services**<br><br>**Fellowes et al., 2003** |

| | | | |
|---|---|---|---|
| | | | 22.3 % of respondents reported referring to a Macmillan nurse.<br><br>“When asked for any other reasons that had prevented or delayed referral to the MCNS, the main reason given was referral to other professionals such as local services.”<br><br>**(M/C) Accounting for and dissatisfaction with service limitations**<br><br>**Fellowes et al., 2003**<br><br>46.0% reported that referrals are often delayed or prevented because the service has no capacity (no Marie Curie nurse available) when needed.<br><br>“However, district nurses expressed some dissatisfaction over the availability of the MCNS for rural areas, for holiday periods, for day care, for regular respite care, and for care at short notice.”<br><br>**(M/C) The need to coordinate care provision from several services**<br><br>**Fellowes et al. 2003**<br>“They also highlighted problems that arose when trying to coordinate a number of palliative care services for 24-hour care.”<br><br>*“Unfortunately, Marie Curie cannot provide 24-hour care in exceptional circumstances. With help from Social Work Department and Hospice at Home, a complete package of care can be provided … it’s very stressful for district nurses not knowing until the last minute what care can be provided, but overall the service is wonderful.”* (free-text response) |

| | | | |
|---|---|---|---|
| **2.8.** | **IT systems and their potential for improving referrals at the end of life** | Bede et al. 2010 (trigger) | *Note, the review team: PEoLC interventions whose programme theories involve the use of information technology are highly prominent in the UK, with a national drive to develop Electronic Palliative Care Coordination Systems since 2008. In another study of members of this team, we have identified over 500 challenges to and over 320 drivers of developing and implementing EPaCCS and data sharing projects more broadly. The articulation of CMOs around IT systems in PEoLC is a significant independent project.* |
| **2.9.**<br><br>**A, M, O** | **Early referral for home care in relation to home death**<br><br>Early referral to services supporting home care (A) may reduce the likelihood that a patient dies at home (O). This may be because families and/or professionals find it difficult to sustain care at home for extended periods of time (M). | Higginson and Wilkinson 2002<br><br>Grande et al. 2003 | **(A-O association) Early referral – lesser likelihood of home death**<br><br>**Higginson and Wilkinson 2002**<br><br>*(Setting/ participants) Marie Curie Nursing Services in the UK (all four countries). At the time (late 90s) 5000 part-time nurses providing hands-on 24-hour palliative nursing care (though not specialist) for patients with advanced and progressive cancer and their families, particularly in period leading up to death; registered, enrolled nurses or care assistants; referral through primary care team, particularly district nurses.*<br><br>*NB: 2019 numbers of Marie Curie nurses are roughly half that figure, although hours unclear – 2,160 (https://www.mariecurie.org.uk/who/what-we-do, accessed 24 Jun 19)*<br><br>*(Methods) Data from the Marie Curie NurseLink system used by 147 out of 220 NHS trusts using Marie Curie nurses (67%; 65% of Marie Curie Nursing Services budget);*<br>*data collected on all patients referred to the service between 1 Jan 1997 and 28 Feb 1999*<br>*26 months*<br>*total of 26,632 patients;* |

| | | | |
|---|---|---|---|
| | | | *data on cancer death registrations in England for 1997, from ONS (Office of National Statistics); statistical analysis – frequency distributions, univariate analysis and multivariate analysis on predictors of not dying at home;*<br><br>*high level of missing data for place of death (recorded 13, 311, missing for 13,321) and of these 94% died at home.*<br><br>Shorter time between referral and death found to be associated with home death (OR 0.99; CI 0.99 – 0.99, narrow CI explained by high numbers in sample).<br><br>**Grande et al. 2003**<br><br>*(Setting/ participants) Cambridgeshire, UK; cancer patients. All National Health Service home care received in the last year of life for patients who died at home vs. patients who died in inpatient care (the latter including hospital, hospice, nursing or residential home, or in transition).*<br><br>*(Methods) Post hoc exploratory case-control study of 127 home deaths and 200 inpatient deaths. 16-month period. One sample of patients referred to a local Hospital at Home service for palliative care (121). The other sample of patients from a local cancer registry (206). Retrospective electronic record linkage of patients' community and inpatient care during the last year of life (robust process of identifying and matching records).*<br><br>*Service type* — ***Start of service as median days before death*** *for patients to this service in last year of life* — *Significance*<br>*Home death (N)* — *Inpatient death (N)*<br>*District nursing* — *71 days (104)* — *141.5 days (132)* — *p=0.001* |

| | | | |
|---|---|---|---|
| | | | *Night nursing 7.5 days (32) 26 days (17) p=0.001*<br><br>*Macmillan 91.5 days (40) 81.5 days (32) non-sign*<br><br>*Marie Curie 12 days (63) 30.5 days (30) p=0.008*<br><br>*Other community trust care 38 days (19) 58 days (18) non-sign*<br><br>*Flexible care 20 days (18) 50.5 days (12) non-sign*<br><br>*Hospital@Home nursing 6 days (51) 21 days (10) p=0.005*<br><br>For inpatient services, data on inpatient hospice care show, in contrast, that starting care earlier is associated with home death (median of 35 days for the patients who died at home and 12 days for the patients who died as inpatients). Receiving care from the specialist hospital teams also shows a tendency towards increasing likelihood of home death (p=0.07)<br><br>*Explanation of pattern of data*<br><br>“Patients who began their home nursing early were less likely to die at home than those who began such care late. This suggests that it may be difficult to sustain end-of-life care at home for an extended period.”<br><br>“This may be due to the strain this places on informal care resources, as family carers shoulder the greater part of the burden.” |
| **2.10.**<br><br>**A, C, M, O** | **Earlier and later referral relative to time of diagnosis**<br><br>At least in cancer patients (C), later referral to a palliative care physician relative to the time of diagnosis (A) may increase the likelihood that a | Maida 2002 BROADER (Canada) | **(A-O association) Longer time between diagnosis and referral and likelihood of home death**<br><br>**Maida 2002** |

| | | | |
|---|---|---|---|
| | patient dies at home (O). This may be because they have developed greater acceptance of their terminal prognosis (M/C) and/or because have been "through more trials, tribulations, and treatment failures, and spent more time in institutions" (M/C). They may thus be more likely to seek, accept and plan for home-based palliative care as opposed to more invasive, hospital-based care with curative or life-prolonging goals (M). | | *(Setting/ participants) Combined community and hospital-based palliative care service in Toronto, Ontario, Canada. Patients residing in five districts of metropolitan Toronto, within 20 km radius of base hospital. Referrals made by oncologists, surgeons and general practitioners. Patients seen initially within 24 hours of referral. Around the clock on-call coverage by the palliative care physicians (unclear if only at home). All patients receive regularly scheduled RN visits. Patients aware of terminal diagnosis and expressed a preference to die at home.*<br><br>*(Methods) Retrospective chart review (both medical and nursing charts) of 402 sequential cancer patients referred to service. Period between 1 Apr 1997 and 8 Apr 1999 (24 months). Further information collected at visits (e.g. number of caregivers) or from web-based sources (Toronto Real Estate Board's web-service for home valuation). Analysis in SAS, both parametric and non-parametric.*<br><br>Mean number of months between diagnosis and referral: 27.3 months, SD 2.34 for those who died at home vs. 11.6, SD 2.61 for those who died at hospital; $p < 0.001$. *(Note it is not months before death, but between diagnosis and referral, i.e. potential "delay" in referral)*<br><br>**(M/C) Period of time since diagnosis in relation to acceptance of death and desire to avoid the downsides of treatment**<br><br>**Maida 2002**<br>(explanation of pattern of data) Longer period of time between diagnosis and referral to palliative care physician means that such patients will have lived "with the knowledge and ramifications of their condition for longer". This has given them the opportunity to develop greater acceptance of their terminal prognosis. Such patients have, overall, also gone "through more trials, tribulations, and treatment failures, and spent more time in institutions". Perhaps they had a greater desire to return home finally to die. (p. 285) |

## 3. Supplementary Table 2: Abstract-level scoping of systematic reviews on prognosis from the last 5 years (2015-2020)

This table includes core information from a scoping search on recent research on prognosis, relative to expectations that advancements in research can point towards better prognostic tools/ approaches for identifying patients at the end of life.

The search strategy is described in the Literature searching appendix (Appendix 4). We have included abstracts of papers published between 2015 and 2020. We excluded: non-systematic reviews (e.g. narrative reviews or supposedly systematic reviews using a single database); research on children; papers on procedures with curative intent and/or exclusive focus on longer survival timeframes (e.g. 5 or 10 years). In cases where overall survival at 5 or 10 years was rather low, e.g. below 20%, we did, however, include the papers. We excluded studies we have already reported on in the CMO table above.

For the needs of this preliminary scoping search, we only accessed the abstracts of papers. If the paper had no abstract or it did not provide sufficient information about its findings, it was not added to the table.

Some frequent abbreviations in the authors' abstracts are:

OS – overall survival
DFF – disease free survival
PFS – progression free survival
RFS – relapse free survival
DSS – disease specific survival

| Reference (authors and year) | Condition, condition + therapy, or setting | No studies reviewed | No participants | Predictors of survival or predictive approach explored | Findings | Broad conclusion |
|---|---|---|---|---|---|---|
| 1. Bu et al. 2015 | malignant tumors | 30 | 4497 | MicroRNA-126 | "[H]igh level of miR-126 was a predictor for favorable survival of carcinomas, with pooled HR of 0.77 (95% CI 0.64-0.93) for OS, 0.64 (95%CI 0.48-0.85) for DFS, and 0.70 (95% CI 0.50-0.98) for PFS/RFS/DSS."<br><br>"However, high level of circulating miR-126 predicted a significantly worse OS in patients with cancer (HR = 1.65, 95% CI 1.09-2.51)." | Single marker (i.e. relative importance unclear), prognostic/ predictive value confirmed, but opposing valence depending on context |
| 2. Li et al. 2015 | non-small cell lung cancer | 10 | | rapamycin (mTOR) and phosphorylated mTOR (p-mTOR) | "The results indicated that no statistically significant association was found between mTOR/p-mTOR expression and NSCLC patients' prognosis." | No/ insufficient/ weak evidence |
| 3. Petrelli et al. 2015 | survival with bevacizumab-based therapy in colorectal cancer patients | 29 | 11,585 | Potential prognostic factors in 3 or more source papers (see column to the right) | Five parameters were associated with survival in >=3 papers:<br><br>(1) a longer progression-free interval [PFS: HR 0.87, 95 % confidence interval (CI) 0.78-0.97; P = 0.01];<br><br>(2) a single site of metastases (HR 0.63, 95 % CI 0.56-0.71; P < 0.00001); | Prognostic/ predictive value of small set of parameters confirmed, highly specific context of condition and/or therapy |

| | | | | | | |
|---|---|---|---|---|---|---|
| | | | | | (3) elevated lactate dehydrogenase (LDH: HR 2.08, 95 % CI 1.69-2.57; P < 0.00001);<br><br>(4) KRAS mutation (HR 1.66, 95 % CI 1.36-2.03; P < 0.00001);<br><br>(5) poor performance status (PS: HR 1.99, 95 % CI 1.41-2.82; P < 0.0001).<br><br>"Clinical variables associated with prolonged survival, after first-line treatment with chemotherapy + BEV for metastatic CRC patients, included long PFS, low LDH levels, KRAS wild-type status, good PS and a single site of metastasis." | |
| 4. Qi et al. 2015 | Budd-Chiari syndrome | 79 studies | | 1-, 5- and 10-year survival relative to treatment modality<br><br>Bilirubin<br>creatinine<br>ascites | Median 1-year survival rate:<br><br>93% after interventional radiological treatment<br>81% after surgery other than liver transplantation<br>82.5% after liver transplantation<br>68.1% after medical therapy alone.<br><br>"Bilirubin, creatinine and ascites were more frequently identified as significant prognostic factors in univariate analyses. But their statistical significance was less frequently achieved in multivariate analyses." | No/ insufficient/ weak evidence |
| 5. Tang et al. 2015 | Breast cancer | 41 articles | | MicroRNAs (miRNAs) – 27 types, as identified in the source studies, with | "For the studies evaluating miR-21's association with clinical outcomes, the median HR in the studies was 2.32 (interquartile range [IQR] = 1.04-3.40), and the pooled HR suggested that high expression of miR-21 has a negative | Single marker (i.e. relative importance unclear), prognostic/ |

| | | | | | | |
|---|---|---|---|---|---|---|
| | | | | micRNA-21 (miR-21) studied most often | impact on overall survival (OS; HR = 1.46, 95% CI, 1.25-1.70; p<0.05) and disease/recurrence-free survival in breast cancer (HR = 1.49, 95% CI, 1.17-1.90; p<0.01).”<br><br>“We also found that higher expression levels of miR-210 significantly predicted poorer outcome, with median HR in the reported studies of 4.07 (IQR = 1.54-4.43) and a pooled HR of 2.94 (95% CI, 2.08-4.17; p<0.05).” | predictive value confirmed |
| 6. Thuy et al. 2015 | Glioblastoma multiforme (GBM) | | 14,678 | 39 unique genetic or molecular factors | A few hundred genetic and molecular predictors have been implicated in the literature, however with the exception of IDH1 and O6-MGMT, there is uncertainty regarding their true prognostic relevance.<br><br>Seventy-four random effects meta-analyses were performed on 39 unique genetic or molecular factors.<br><br>(no further findings reported in abstract) | Multiple (>30) prognostic/ predictive factors, findings unclear from abstract |
| 7. Wang et al. 2015 | cancer | 15 | 2,597 | MicroRNA-34a (miR-34a) | “Overexpression of miR-34a may predict good overall survival ([OS], HR =0.76, 95% confidence interval: 0.55-1.06, P=0.105), but the effect was not significant enough. Subgroup analysis results showed miR-34a was an ideal predictor for digestive system cancer (OS, HR =0.50, 95% confidence interval: 0.25-0.99, P=0.048). The predictive effects of elevated expression of miR-34a on the OS of untreated and treated patients were not of obvious differences.” | Single marker (i.e. relative importance unclear), weak evidence for overall predictive/ prognostic value, strong evidence for a specific context |

| | | | | | | |
|---|---|---|---|---|---|---|
| 8. Wei and Niu 2015 | Various cancers | 9 | 932 | metastasis-associated lung adenocarcinoma transcript 1 (MALAT1) | "Elevated MALAT1 expression was significantly correlated with poor OS (HR 2.02; 95% CI: 1.62-2.52; P < 0.001; I(2) = 0%). Subgroup analysis indicated that tumor type, histology type, ethnicity, and measurement technique did not affect the prognostic value of MALAT1 for OS. The HR of elevated MALAT1 for DFS was 2.78 (95% CI: 1.87-4.15; P < 0.001; I(2) = 0%)." | Single marker (i.e. relative importance unclear), prognostic/ predictive value confirmed |
| 9. Zhou C et al. 2015 | patients with liver diseases who have undergone transjugular intrahepatic portosystemic shunt (TIPS) | 11 | | Comparison of the *Child-Turcotte-Pugh* (CTP) score and the *model for end-stage liver disease* (MELD) score for predicting survival in such patients | "In the meta-analyses, MELD score was superior to CP score in predicting 3-month survival after TIPS (mean ES, 0.63; 95% confidence interval [CI], 0.13-1.14; P=0.01), but the predictive capability in 1-month, 6-month, and 12-month survival was not significant (1-month: mean ES, 0.79; 95% CI, -0.24-1.83; P=0.13; 6-month: mean ES, 0.46; 95% CI, -2.46-3.37; P=0.76; 12-month: mean ES, 0.36; 95% CI, -0.25-0.96; P=0.25).<br><br>CONCLUSIONS: No enough evidence are confirmed so far that MELD score is better than CTP score to assess the overall prognosis after TIPS, especially long-term predictions, but 3-month predictive capability of MELD score significantly outperform CTP score." | Tools (scores, indices) – inconsistent or poor performance |
| 10. Cheon et al. 2016 | Advanced cancer | 15 | | Clinicians' predictions of survival | "Clinicians in five studies underestimated patients' survival (estimated to observed survival ratio between 0.5 and 0.92). In contrast, 12 studies reported clinicians' overestimation of survival (ratio between 1.06 and 6)."<br><br>"CPS in advanced cancer patients is often inaccurate and overestimated." | Inaccuracy of clinician predictions |

| | | | | | | |
|---|---|---|---|---|---|---|
| 11. Lee et al. 2016 | early-stage endometrial cancer | 11 | | positive peritoneal cytology | “In patients with surgical stage 1 or 2 early-stage endometrial cancer, the incidence of Grade 3 was higher and 5-year overall survival was worse in patients with positive peritoneal cytology compared with negative peritoneal cytology. However, the incidence of Grade 1 was lower in those with positive peritoneal cytology compared with negative peritoneal cytology. In patients with surgical stage 1 early-stage endometrial cancer, the incidence of myometrial invasion >=1/2 tended to be higher and 5-year progression-free survival was worse in the positive peritoneal cytology group than the negative peritoneal cytology group. However, the incidence of myometrial invasion <1/2 was lower in the positive peritoneal cytology group than the negative peritoneal cytology group.” | Single marker (i.e. relative importance unclear), prognostic/ predictive value confirmed, but opposing valence depending on context |
| 12. Leuzzi et al. 2016 | non-small-cell lung cancer (NSCLC) | 10 | | baseline C-reactive protein (CPR) | “In overall analysis, elevated pretreatment CRP values were significantly associated with poor overall survival (HR 1.60, 95% CI 1.30-1.97, p<0.001, I2 = 71.9%). Similar results were observed across considered strata. However, higher mortality risk was reported in studies in which CRP was combined with other factors (HR 1.96, 95% CI 1.58-2.45) and in those using a cutoff value of 3 mg/L (HR 1.89, 95% CI 1.52-2.35).<br>CONCLUSIONS: Based on our analysis, baseline high CRP level is significantly associated with poor prognosis in early-stage NSCLC. Further prospective controlled studies are needed to confirm these data.” | Single marker (i.e. relative importance unclear), prognostic/ predictive value confirmed, further research recommended |

| | | | | | | |
|---|---|---|---|---|---|---|
| 13. Marques et al. 2016 | Head and neck cancer | 12 | | mTOR pathway protein immunoexpression | "The meta-analysis revealed that the frequency of overall expression of mTOR pathway proteins was 74.42% (CI: 63.3 to 84.0, P < 0.001, n = 2016 samples). The survival meta-analysis showed a pooled hazard ratio for OS and DFS of 1.44 (95% confidence interval [95% CI] 1.14-1.73) and 1.18 (95% CI 0.71-1.64), respectively.<br>CONCLUSION: This systematic review and meta-analysis support evidence that mTOR pathway proteins can be used as predictive markers for survival in patients with HNC because their expression was significantly associated with poor OS and short DFS." | Single marker (i.e. relative importance unclear), prognostic/ predictive value confirmed |
| 14. Qian et al. 2016 | systemic lupus erythematosus (SLE)-associated pulmonary arterial hypertension (PAH) | 6 | 323 | Main study goal to assess survival of such patients; predictors as identified in literature | "WHO Functional class (Fc) III/IV was found to be an independent prognostic factor of mortality. Higher mean pulmonary arterial pressure (mPAP), higher pulmonary vascular resistance (PVR), lower six minutes walking distance (6MWD), higher brain natriuretic peptide (BNP) and higher N-terminal proBNP (NT-proBNP) level were also related to poor survival." | Prognostic/ predictive value of small set of parameters confirmed, highly specific context of condition and/or therapy |
| 15. Shan et al. 2016 | Cancer | 44 | | Dicer status | "24 of 44 articles revealed low Dicer status as a predictor of poor prognosis. The aggregate result of overall survival (OS) indicated that low Dicer expression level resulted in poor clinical outcomes, and subgroup of IHC and RT-PCR method both revealed the same result. Overall analysis of progression-free survival (PFS) showed the same result as OS, and both the two subgroups divided by laboratory method revealed positive results. Subgroup analysis by tumor types showed low dicer levels were | Single marker (i.e. relative importance unclear), prognostic/ predictive value confirmed, further research recommended |

| | | | | | | |
|---|---|---|---|---|---|---|
| | | | | | associated with poor prognosis in ovarian cancer (HR = 1.93, 95% CI: 1.19-3.15), otorhinolaryngological tumors (HR = 2.39, 95% CI: 1.70-3.36), hematological malignancies (HR = 2.45, 95% CI: 1.69-3.56) and neuroblastoma (HR = 4.03, 95% CI: 1.91-8.50).<br>CONCLUSION: Low Dicer status was associated with poor prognosis in ovarian cancer, otorhinolaryngological tumors and hematological malignancies. More homogeneous studies with high quality are needed to further confirm our conclusion and make Dicer a useful parameter in clinical application." | |
| 16. Wu et al. 2016 | Cancer – 11 different tumor types | 18 | | high mobility group box 1 (HMGB1) overexpression | "HMGB1 overexpression was significantly associated with poorer OS (HR: 1.99; 95% CI, 1.71-2.31) and PFS (HR: 2.26; 95% CI, 1.65-3.10) irrespective of cancer types including gastric cancer, colorectal cancer, hepatocellular carcinoma, pancreatic cancer, nasopharyngeal carcinoma, head and neck squamous-cell carcinoma, esophageal cancer, malignant pleural mesothelioma, bladder cancer, prostate cancer, and cervical carcinoma. Subgroup analyses indicated geographical area and size of studies did not affect the prognostic effects of HMGB1 for OS. Morever, HMGB1 overexpression had a consistent correlation with poorer OS when detected by immunohistochemistry in tissues and enzyme-linked immunosorbent assay in serum, whereas the correlation did not exist by quantitative real-time reverse-transcription polymerase chain reaction in tissues. HMGB1 overexpression is associated with poorer | Single marker (i.e. relative importance unclear), prognostic/ predictive value confirmed |

| | | | | | | |
|---|---|---|---|---|---|---|
| | | | | | prognosis in patients with various types of cancer, suggesting that it is a prognostic factor and potential biomarker for survival in cancer.” | |
| 17. Chen et al. 2017 | cancer | 7 | 652 | Long noncoding RNA HOTTIP - HOXA transcript at the distal tip (HOTTIP), a functional lncRNA transcribed from the 5' tip of the HOXA locus | “The results showed a significant positive association between HOTTIP levels and LNM (Odds ratio, OR = 2.30, 95 % CI: 1.58-3.35, p < 0.0001) in a fixed-effects model (I2 = 0 %, p = 0.949) and it could also predict poor OS in cancer patients (Hazard ratio HR = 2.24, 95% CI: 1.74-2.90, p < 0.00001) in a fixed-effects model (I2 = 0%, p = 0.925). In conclusion, this meta-analysis demonstrated that the higher expression level of HOTTIP is correlated with positive LNM and poor OS in different types of cancer and HOTTIP might serve as a novel predictor of LNM and survival in human cancer.”<br><br>LNM = lymph node metastasis | Single marker (i.e. relative importance unclear), prognostic/ predictive value confirmed |
| 18. Mahar et al. 2017 | Colorectal cancer | | | Clinical prognostic tools for survival outcomes | Significant heterogeneity in colorectal cancer prognostication tool quality exists. Methodology is incompletely or inadequately reported. Evaluations of the internal or external validity of the prognostic model are rarely performed. Prognostication tools are important devices for patient management, but tool reliability is compromised by poor quality. Guidance for future development of prognostication tools in colorectal cancer is needed. | Tools (scores, indices) – inconsistent or poor performance |
| 19. Nater et al. 2017 | symptomatic spinal metastasis (SSM) | 17 | | preoperative predictors of survival, neurological, functional and | 46 predictors of survival identified<br><br>The strength of the overall body of evidence was very low for 39 and low for 7 predictors. | No/ insufficient/ weak evidence |

| | | | | | | |
|---|---|---|---|---|---|---|
| | | | | HRQoL outcomes in surgical patients with SSM | | |
| 20. Petrelli et al. 2017 JAMA Oncology | Colon cancer | 66 | 1437846 | Primary tumor location (Left-Sided vs Right-Sided) | “Left sided primary tumor location was associated with a significantly reduced risk of death (HR, 0.82; 95% CI, 0.79-0.84; P < .001) and this was independent of stage, race, adjuvant chemotherapy, year of study, number of participants, and quality of included studies.” | Single marker (i.e. relative importance unclear), prognostic/ predictive value confirmed |
| 21. Petrelli et al. 2017 Urology | radical nephroureterectomy for upper urinary tract urothelial carcinoma | | | Prognostic factors that influence overall survival described in the literature | “The clinicopathological factors associated with an increased risk of death were age, multifocality, lymphovascular invasion, pT3-4 stage, pT2 vs <pT2 stage, node-positive disease, tumor grade, tumor size, and positive surgical margins.” | Single marker (i.e. relative importance unclear), prognostic/ predictive value confirmed |
| 22. Smith et al. 2017 | COPD | 10 | | known prognostic variables and scores that predict prognosis in COPD, specifically including variables that contribute to risk assessment of patients for death within 12 months | “No multivariable indices were developed with the specific aim of predicting all-cause mortality in stable COPD within 12 months. Only nine indices were identified from four studies, which had been validated for this time period. Tools developed using expert knowledge were also identified, including the Gold Standards Framework Prognostic Indicator Guidance, the RADboud Indicators of Palliative Care Needs, the Supportive and Palliative Care Indicators Tool and the Necesidades Paliativas program tool.<br>CONCLUSION: A number of variables contributing to the prediction of all-cause mortality in COPD were identified. However, there are very few studies that are designed to | Tools (scores, indices) – unspecific |

| | | | | | | |
|---|---|---|---|---|---|---|
| | | | | | assess, or report, the prediction of mortality at or less than 12 months. The quality of evidence remains low, such that no single variable or multivariable score can currently be recommended.” | |
| 23. Urun et al. 2017 | advanced urothelial cancer treated with platinum based chemotherapy | 13 | 1475 | excision repair cross-complementing group 1 (ERCC1) | “ERCC1 positivity was significantly associated with worse progression-free survival (pooled HR: 1.54, 95% CI: 1.13-2.11, p=0.006). There was no significant association with overall survival (pooled HR1.63, 95% CI: 0.93-2.88, p=0.09) and disease-free survival (pooled HR: 1.092, 95% CI: 0.63-1.90, p=0.75).” | No/ insufficient/ weak evidence |
| 24. Yang et al. 2017 | epithelial ovarian cancer | 12 | 3,154 | neutrophil-to-lymphocyte ratio (NLR) | “Elevated NLR in EOC patients was associated with worse PFS (summarized HR=1.80; 95% CI = 1.22-2.65; I2 = 79.1%) and OS (summarized HR = 1.72; 95% CI = 1.18-2.51; I2 = 73.5%) compared with low NLR. No evidence of publication bias was detected by funnel plot analysis and formal statistical tests. Although the results were robust in all subgroup analyses, not all results were statistically significant. We determined that adjustments for CA-125 level and performance status might be sources of heterogeneity. These combined results indicate that preoperative NLR is an important predictor of prognosis in EOC patients. Since the high heterogeneity and retrospective study design of included studies, these results require further validation with prospective cohort and trials enrolling larger patient populations and conducting longer follow-up examinations.” | Single marker (i.e. relative importance unclear), prognostic/ predictive value confirmed, further research recommended |

| | | | | | | |
|---|---|---|---|---|---|---|
| 25. Zabaleta et al. 2017 | pulmonary metastasectomy in colorectal cancer patients | 28 | | history of resected liver metastases | "In more than half of the papers analyzed (63.2%), patients with a history of resected liver metastases had a lower survival rate than those who did not have such a history, and the difference was statistically significant in eight of these studies. However, data were presented differently, and authors reported mean survival time, survival rates, or hazard ratios. CONCLUSIONS: A history of liver metastases seems to be a negative prognostic factor, but the individual data need to undergo a meta-analysis." | Single marker (i.e. relative importance unclear), prognostic/ predictive value confirmed, further research recommended |
| 26. Zhang et al. 2017 | Colorectal cancer | 23 | | Metabolomic profile of patients | "Metabolites related to cellular respiration, carbohydrate, lipid, protein and nucleotide metabolism were significantly altered in CRC. Altered metabolites were also related to prognosis, survival and recurrence of CRC. This review could represent the most comprehensive information and summary about CRC metabolism to date. It certificates that metabolomics had great potential on both discovering clinical biomarkers and elucidating previously unknown mechanisms of CRC pathogenesis." | Prognostic/ predictive value confirmed (5 types of factors) |
| 27. Zhao et al. 2017 | pulmonary metastasectomy in renal cell cancer patients | 16 | 1447 | prognostic factors as identified in the literature | "The poor prognostic factors were lymph node involvement (LNI) of primary RCC (HR 3.44, 95% confidence interval (CI) 1.78-6.67, P = 0.001), incomplete resection of metastases (HR 3.74, 95% CI 2.49-5.61, P = 0.000), multiple metastases (HR 1.55, 95% CI 1.18-2.03, P = 0.002), larger metastases (HR 1.45, 95% CI 1.26-1.66, P = 0.000), LNI of metastases (HR 3.06, 95% CI 1.52-6.19, P = 0.002), synchronous | Prognostic/ predictive value of small set of parameters confirmed, highly specific context of condition and/or therapy |

| | | | | | | |
|---|---|---|---|---|---|---|
| | | | | | metastasis (HR 2.49, 95% CI 1.46-4.24, P = 0.001) and short disease free interval (DFI)." | |
| 28. Bollen et al. 2018 | Spinal bone metastases | 22 | | "A total of 43 different prognostic factors were investigated in the included studies, of which 17 were relevant to pre-treatment survival estimation." | "The prognostic factors most frequently associated with survival were the primary tumor and the performance status. The prognostic factors most frequently not associated with survival were age, gender, number and location of the SBM and the presence of a pathologic fracture. Conclusions: Prognostication for patients with SBM should be based on an accurate primary tumor classification, combined with a performance score. The benefit of adding other prognostic factors is doubtful." | Familiar parameters validated from a large number of candidates |
| 29. Bosma et al. 2018 | Ewing sarcoma | 21 | | "24 prognostic factors were investigated, 14 relevant for this review" | "Prognostic factors associated with survival include metastasis at diagnosis, large tumors (volume>=200ml or largest diameter>=8cm), primary tumors located in the axial skeleton, especially pelvic and a histological response of less than 100%. These factors should be included as risk factors in the development of prediction models for ES." | Prognostic/ predictive value of small set of parameters confirmed, highly specific context of condition and/or therapy |
| 30. Chi et al. 2018 | cancer | 14 | 4136 | albumin to globulin ratio (AGR) | "The analysis based on random-effect model demonstrated that low AGR was significantly associated with poor OS in various cancers (HR=1.87, 95% CI 1.50-2.34; P < 0.001)."<br><br>"[A] large scale of samples and prospective studies are needed in the future to validate the role of AGR in practice." | Single marker (i.e. relative importance unclear), prognostic/ predictive value confirmed, further research recommended |

| | | | | | | |
|---|---|---|---|---|---|---|
| 31. Dan et al. 2018 | cancer | 11 | 1,797 | miR-375 | “The pooled HR for overall/cumulative survival (OS/CS) was 1.90 (95% confidence interval (CI) 1.57-2.29) and the pooled HR for disease-free, recurrence-free or progression-free survival (DFS/RFS/PFS) was 1.93 (95% CI 1.39-2.67), indicating low miR-375 expression was associated with significantly poorer outcomes compared to normal/high miR-375 expression. Subgroup analysis revealed miR-375 might be a good prognostic factor in cancer, regardless of population, sample type, and cancer type. The prognostic value of miR-375 in non-Chinese patients was particularly high (pooled HR > 2). CONCLUSION: Low miR-375 expression could represent a valuable prognostic marker in various cancers. Circulating miR-375 levels may provide a useful non-invasive, practical prognostic biomarker. However, the prognostic value of miR-375 in specific cancer types remains unclear; further studies are warranted.” | Single marker (i.e. relative importance unclear), prognostic/ predictive value confirmed, further research recommended |
| 32. Georgakis et al. 2018 | Gliomatosis cerebri (GC) | | 523 patient level data | | The median OS and PFS were 13 and 10 months, with 5-year rates of 18% and 13%, respectively.<br><br>Age >=65 years at diagnosis (hazard ratio for OS [HR<sub>OS</sub>], 2.32; 95% confidence interval [CI], 1.62-3.31),<br><br>high-grade tumor (HR<sub>PFS</sub> for grade III, 1.57; 95% CI, 1.02-2.40; HR<sub>PFS</sub> for grade IV, 1.74; 95% CI, [0.98-3.10), | Prognostic/ predictive value confirmed (16 factors) |

| | | | | | | |
|---|---|---|---|---|---|---|
| | | | | | GC type II (HR<sub>OS</sub>, 1.49; 95% CI, 1.12-1.98; HR<sub>PFS</sub>, 1.56; 95% CI, 1.04-2.34),<br><br>more central nervous system (CNS) regions involved (HR<sub>OS</sub>, 1.09; 95% CI, 1.01-1.18),<br><br>focal neurological deficits (HR<sub>OS</sub>, 1.41; 95% CI, 1.07-1.86),<br><br>cerebellar symptoms (HR<sub>PFS</sub>, 2.20; 95% CI, 1.42-3.39),<br><br>more symptoms at presentation (HR<sub>OS</sub>, 1.21; 95% CI, 1.05-1.40),<br><br>Karnofsky performance scale score <70 (HR<sub>OS</sub>, 3.58; 95% CI, 1.73-7.39; HR<sub>PFS</sub>, 4.48; 95% CI, 1.39-14.4),<br><br>magnetic resonance imaging contrast enhancement (HR<sub>OS</sub>, 1.48; 95% CI, 1.12-1.96; HR<sub>PFS</sub>, 1.74; 95% CI, 1.18-2.55),<br><br>symmetric bilateral CNS invasion (HR<sub>OS</sub>, 1.42; 95% CI, 1.03-1.96),<br><br>and high proliferation index (Ki-67 >5%; HR<sub>OS</sub>, 2.32; 95% CI, 1.11-4.86)<br><br>were independent predictors of poor outcomes.<br><br>In contrast, | |

| | | | | | | |
|---|---|---|---|---|---|---|
| | | | | | seizure occurrence (HR<sub>OS</sub>, 0.77; 95% CI, 0.60-1.00; HR<sub>PFS</sub>, 0.68; 95% CI, 0.47-0.95),<br><br>isocitrate dehydrogenase 1 mutation (HR<sub>OS</sub>, 0.16; 95% CI, 0.05-0.49),<br><br>and O6-methylguanine-DNA-methyltransferase promoter methylation (HR<sub>OS</sub>, 0.23; 95% CI, 0.09-0.59)<br>were associated with prolonged survival.<br><br>Chemotherapy and surgical resection were associated with improved outcomes, but radiotherapy, whether monotherapy or combined with chemotherapy, was not superior to chemotherapy alone.<br><br>[text broken, otherwise identical to original] | |
| 33. Huang et al. 2018 | Lung cancer | 55 | 22,719 | decreased pretreatment haemoglobin level | "The results indicated that decreased haemoglobin level was significantly associated with poor overall survival of patients with lung cancer (HR 1.51, 95% CI 1.42-1.61), both in non-small cell lung cancer (HR 1.57, 95% CI 1.44-1.72) and in small cell lung cancer (HR 1.56, 95% CI 1.21-2.02). We also found that the lower the haemoglobin level, the shorter was the overall survival of patients with lung cancer (HR 1.11, 95% CI 1.06-1.16)." | Single marker (i.e. relative importance unclear), prognostic/ predictive value confirmed |
| 34. Kloter et al. 2018 | cancer | 19 | | Heart rate variability | "[H]igher HRV correlated positively with patients' progression of disease and outcome. Thus, we conclude that individuals with higher HRV and advanced coping mechanisms seem to have a better prognosis in cancer progression." | Single marker (i.e. relative importance unclear), prognostic/ |

| | | | | | | |
|---|---|---|---|---|---|---|
| | | | | | | predictive value confirmed |
| 35. Lakshminarayana et al. 2018 | oral squamous cell carcinoma (OSCC) | 36 | | Molecular pathways and genes involved in oral cancer | "Three major interlinked pathways found were the nuclear factor kappa B (NF-kB), PI3K-AKT, and Wnt pathways. The commonly mutated genes were cyclin D1 (CCND1), Rb, p53, FLJ10540, and TC21. The NF-kB, PI3K-AKT, and Wnt pathways are most frequently involved in the molecular pathogenesis of oral cancer. However, the CCND1, Rb, p53, FLJ10540, and TC21 genes were found to be more accurate in determining patients' overall survival." | Prognostic/ predictive value of small set of parameters confirmed, highly specific context of condition and/or therapy |
| 36. Li et al. 2018 | Gastric cancer | 8 | 950 | Expression of VEGFRs | "The combined HR of studies evaluating total VEGFRs overexpression was 1.42 (95% CI 1.01-2.00, P=0.044), suggesting that it had prognosis significance in overall survival of gastric cancer. Subgroup analysis showed that it was VEGFR-2 (HR 1.81, 95% CI 1.31-2.49, P<0.001) but not VEGFR-3 (HR 0.91, 95% CI 0.45-1.82, P=0.787) overexpression was associated with an increased risk of median overall survival (mOS) and it can be a potentially predictive biomarker for gastric cancer." | Single marker (i.e. relative importance unclear), prognostic/ predictive value confirmed |
| 37. Liu et al. 2018 | Patients in palliative care | 15 | 7455 assessments | Palliative prognostic index (PPI) | "[F]our studies were assessed by meta-analysis. The sensitivity of the PPI for 3-week survival ranged from 51 to 92% and specificity ranged from 60.0 to 94.0%, respectively. The sensitivity and specificity of the PPI for 6-week survival were from 46.0 to 89.1% and from 51.7 to 84.4%, respectively. The pooled sensitivity and specificity of the PPI for 3-week survival were 68% (6 as cutoff) and 76% (6 as cutoff), respectively. As for 6-week survival prediction, | Standards needed – disagree with authors' criteria of "useful prognosticator" |

| | | | | | | |
|---|---|---|---|---|---|---|
| | | | | | the pooled sensitivity and specificity were 68% (4 as cutoff) and 82% (4 as cutoff), respectively. Conclusion: The PPI is a useful prognosticator of life expectancy of patients in palliative care, especially for patients with short survival time. However, there were no universal cutoff, and the predicted life span varies."<br><br>"Due to small number of studies and poor qualities of them, result may alter as more studies with better quality are enrolled in the future." | |
| 38. Montagnani et al. 2018 | liver resection in metastatic gastric cancer (GC) | 33 | 1304 | Prognostic factors identified in the literature | "Our analysis demonstrates a 5yOS rate of 22% (95%CI: 18-26%) and 10yOS rate of 11% (95%CI: 7-18%) among patients undergoing radical hepatectomy. A favorable effect on OS was shown by several factors linked to primary cancer (lower T and N stage, no lympho-vascular or serosal invasion) and burden of hepatic disease (<=3 metastases, unilobar involvement, greatest lesion<5cm, negative resection margins). Moreover, lower CEA and CA19.9 levels and post-resection chemotherapy were associated with improved OS." | Prognostic/ predictive value of small set of parameters confirmed, highly specific context of condition and/or therapy |
| 39. Pereira et al. 2018 | Advanced cancer (metastatic disease) | 9 | 1496 | Phase angle<br><br>"Phase angle (PA) is a ratio between the reactance and resistance obtained by bioelectric impedance analysis and has been interpreted as a cell | "Low PA was associated with worse nutrition status evaluated by body mass index, serum albumin level, transferrin, and fat-free mass. The median OS of the included papers varied from 25.5–330 days, and all studies analyzed showed a significant association between PA and OS, in that patients with low PA had worse OS. Future studies are necessary to justify the use of PA in therapeutic decisions for this population and to evaluate whether nutrition | Single marker (i.e. relative importance unclear), prognostic/ predictive value confirmed, further research recommended |

| | | | | | | |
|---|---|---|---|---|---|---|
| | | | | membrane integrity indicator and a predictor of total body cell mass. A low PA may suggest deterioration of the cell membrane, which in advanced cancer patients may result in a reduced overall survival (OS).” | status can influence the association between PA and survival.” | |
| 40. Pinart et al. 2018 | metastatic castration-resistant prostate cancer | 12 | 8750 | Prognostic models identified in the literature | “Models included 4-11 predictor variables, mostly hemoglobin, baseline PSA, alkaline phosphatase, performance status, and lactate dehydrogenase. Very few incorporated Gleason score. Two models included predictors related to docetaxel and mitoxantrone treatments. Model performance after internal validation showed similar discrimination power ranging from 0.62 to 0.73. Overall survival models were mainly constructed as nomograms or risk groups/score. Two models obtained an overall judgment of low risk of bias.<br>CONCLUSIONS: Most models were not suitable for clinical use due to methodological shortcomings and lack of external validation. Further external validation and/or model updating is required to increase prognostic accuracy and clinical applicability prior to their incorporation in clinical practice as a useful tool in patient management.” | Most models not suitable for clinical use; further validation/ updating required |
| 41. ter Veer et al. 2018 | metastatic oesophagogastric cancer | 46 | 15,392 | Prognostic and predictive factors as | “Prognostic factors were identified from multivariate regression analyses in study reports. Factors were considered potentially | Multiple factors – potentially |

| | | | | | | |
|---|---|---|---|---|---|---|
| | | | | identified in the source studies | clinically relevant if statistically significant (P ≤ 0.05) in multivariate analysis in ≥50% of the total number of patients in the pooled sample of the RCTs and were reported with a pooled sample size of ≥600 patients in the first-line or ≥300 patients in the beyond first-line setting. Predictive factors were identified from time-to-event stratified treatment comparisons and deemed potentially clinically relevant if the P-value for interaction between subgroups was ≤0.20 and the hazard ratio in one of the subgroups was significant (P ≤ 0.05).”<br><br>“Seventeen prognostic factors for overall survival in the first-line and four in the beyond first-line treatment setting were potentially clinically relevant. Twenty-one predictive factors in first-line and nine in beyond first-line treatment setting were potentially relevant regarding treatment efficacy.”<br><br>“The prognostic and predictive factors identified in this systematic review can be used to characterise patients in clinical practice, be included in future trial designs, enrich prognostic tools and generate hypotheses to be tested in future research to promote patient-centred treatment.” | clinically relevant |
| 42. Wang C et al. 2018 | pulmonary metastasectomy in liver cancer | 17 | 513 | Prognostic factors as identified from the source studies | “The poor prognostic factors were disease-free interval (DFI) < 12 months (HR = 2.421 95% CI 1.384 4.236) and existence of cirrhosis (HR = 1.936 95% CI 1.031 3.636).” | Familiar parameters validated from a large number of candidates |

| | | | | | | |
|---|---|---|---|---|---|---|
| 43. Wang J et al. 2018 | extracorporeal cardiopulmonary resuscitation | 16 | 1162 | Prognostic factors as identified from the source studies | The following survival predictors of ECPR were identified:<br><br>out-of-hospital cardiac arrest (CA) (OR 0.58, 95% CI 0.36-0.93, P = .02)<br><br>in-hospital CA (OR 1.73, 95% CI 1.08-2.77, P = .02)<br><br>witnessed CA (OR 5.2, 95% CI 1.18-22.88, P = .01)<br><br>bystander cardiopulmonary resuscitation (CPR) (OR 7.35, 95% CI 2.32-23.25, P < .01)<br><br>initial shockable rhythm (OR 2.29, 95% CI 1.53-3.42, P < .01)<br><br>1st recorded nonshockable rhythm (OR 0.44, 95% CI 0.29-0.66, P < .01)<br><br>CPR duration (MD -13.84 minutes, 95% CI -21 to -6.69, P < .0001)<br>arrest-to-extracorporeal membrane oxygenation (ECMO) (MD -17.88 minutes, 95% CI -23.59 to -12.17, P < .01)<br><br>PH (MD 0.14, 95% CI 0.08-0.21, P < .01)<br><br>lactate (MD -3.66 mmol/L, 95% CI -7.15 to -0.17, P = .04)<br><br>percutaneous coronary intervention (PCI) (OR 1.63, 95% CI 1.02-2.58, P = .04). | Prognostic/ predictive value confirmed (11 factors) |

| | | | | | | |
|---|---|---|---|---|---|---|
| 44. Yang et al. 2018 | Breast cancer | 11 | 1467 | Circulating cell-free DNA (cfDNA) | “cfDNA was shown to be significantly associated with PFS (HR 2.02, 95% CI 1.51-2.72, P < .001, I = 82%) and OS (HR 1.75, 95% CI 1.01-3.05, P < .001, I = 92%). The results of subgroup analyses also revealed that cfDNA was a good predictor of prognosis in breast cancer patients.<br>CONCLUSION: Our meta-analysis indicated that cfDNA was associated with poor PFS and OS, thus it may help to predict outcomes of patients with breast cancer. However, further studies are needed to confirm our results.” | Single marker (i.e. relative importance unclear), prognostic/ predictive value confirmed, further research recommended |
| 45. Zhang et al. 2018 | cancer | 10 | 913 | LINC00152, a novel long noncoding RNA (lncRNA) | “[E]levated LINC00152 could predict unfavorable OS with pooled HR of 1.66 (95% CI: 1.29-2.13, p<.0001) and poor DFS (HR=2.13, 95% CI: 1.39-3.25, p=.0005) in cancer patients.<br>CONCLUSION: LINC00152 was correlated with advanced clinicopathological features and poor prognosis as a novel predictive biomarker in various cancers.” | Single marker (i.e. relative importance unclear), prognostic/ predictive value confirmed, novel factor, further research required |
| 46. Aleksova et al. 2019 | Heart transplantation | 21 | | Risk prediction scores – 16 identified in the literature | “Seven (44%) scores were validated in external cohorts and 8 (50%) assessed model performance. Overall model discrimination ranged from poor to moderate (C-statistic/area under the receiver operating characteristics 0.54-0.77). The IMPACT score was the most widely validated, was well calibrated in two large registries, and was best at discriminating 3-month survival (C-statistic 0.76). Most scores did not perform particularly well in any cohort in which they were assessed. This review shows that there are insufficient data to recommend | Tools (scores, indices) – inconsistent or poor performance |

| | | | | | | |
|---|---|---|---|---|---|---|
| | | | | | the use of one model over the others for prediction of post-HT outcomes.” | |
| 47. Antoniou et al. 2019 | Abdominal Aortic Aneurysm | 7 | 1,440 | degenerative loss of skeletal muscle | “Patients with low skeletal muscle mass had a significantly higher hazard of mortality than those without low skeletal muscle mass (HR 1.66, 95% CI 1.15-2.40; p = .007). Subgroup analysis including only patients who underwent EVAR showed a marginal survival benefit for patients without low skeletal muscle mass (HR 1.86, 95% CI 1.00-3.43; p = .05). Meta-analysis of two studies found no significant difference in peri-operative mortality (RD 0.04, 95% CI -0.13 to 0.21) and morbidity (OR 1.58, 95% CI 0.90-2.76; p = .11) between patients with and without low skeletal muscle mass. CONCLUSION: There is a significant link between low skeletal muscle mass and mortality in patients undergoing AAA repair. Prospective studies validating the use of body composition for risk prediction after aortic surgery are required before this tool can be used to support decision making and patient selection.” | Single marker (i.e. relative importance unclear), prognostic/ predictive value confirmed, further research recommended |
| 48. Cao et al. 2019 | Cervical cancer | 19 | 6521 | pretreatment thrombocytosis | “The summary results indicated that an elevated platelet count was significantly associated with a poor OS (HR 1.50; 95% CI 1.19-1.88; P = 0.001), PFS (HR 1.33; 95% CI 1.07-1.64; P = 0.010), and RFS (HR 1.66; 95% CI 1.20-2.28; P = 0.002). Sensitivity analysis indicated that the pooled PFS was variable after sequential exclusion of individual studies. The predictive value of pretreatment thrombocytosis on OS differed according to the publication year (P = 0.039), country (P = | Single marker (i.e. relative importance unclear), prognostic/ predictive value confirmed, further research recommended |

| | | | | | | |
|---|---|---|---|---|---|---|
| | | | | | 0.013), and sample size (P = 0.029), and the role of pretreatment thrombocytosis on PFS could be affected by the study quality (P = 0.046). CONCLUSION: The findings of this study indicated that an elevated platelet count before treatment was associated with poor OS, PFS, and RFS. These results require further verification in large-scale prospective studies." | |
| 49. Feng Q et al. 2019 | Primary gastric cancer | 101 model developments and 32 external validations | | Models for predicting overall survival | "The median (range) of training sample size, number of death, and number of final predictors were 360 (29 to 15320), 193 (14 to 9560), and 5 (2 to 53), respectively. Ninety-one models were developed from routine clinical data. Statistical assumptions were reported to be checked in only nine models. Most model developments (94/101) used complete-case analysis. Discrimination and calibration were not reported in 33 and 55 models, respectively. The majority of models (81/101) have never been externally validated. None of the models have been evaluated regarding clinical impact." | Most models not suitable for clinical use; further validation/ updating required |
| 50. Feng W et al. 2019 | patients with metastatic colorectal cancer receiving bevacizumab as first-line chemotherapy | 7 | 1,219 | serum lactate dehydrogenase (LDH) level | "Meta-analysis of all studies revealed that high serum LDH level is associated with shorter PFS (HR: 1.43, 95% CI: 1.05-1.94; P=0.023) and OS (HR: 1.667, 95% CI: 1.230-2.259; P=0.001) times in mCRC patients treated with bevacizumab-based first-line chemotherapy. However, there was no significant association between serum LDH and objective response rate. Conclusions: High serum LDH level is significantly associated with shorter PFS and OS time and may have utility as a prognostic factor for mCRC patients receiving bevacizumab as first-line chemotherapy and as a predictive | Single marker (i.e. relative importance unclear), prognostic/ predictive value confirmed |

| | | | | | | |
|---|---|---|---|---|---|---|
| | | | | | factor for those receiving bevacizumab-based therapy at other times." | |
| 51. Fernando et al. 2019 | in-hospital cardiac arrest | 23 | | Pre-arrest and intra-arrest prognostic factors | Pre-arrest factors associated with reduced odds of survival after in-hospital cardiac arrest:<br><br>- male sex (odds ratio 0.84, 95% confidence interval 0.73 to 0.95, moderate certainty),<br><br>- age 60 or older (0.50, 0.40 to 0.62, low certainty),<br><br>- active malignancy (0.57, 0.45 to 0.71, high certainty),<br><br>- history of chronic kidney disease (0.56, 0.40 to 0.78, high certainty)<br><br>Intra-arrest factors associated with increased odds of survival:<br><br>- witnessed arrest (2.71, 2.17 to 3.38, high certainty),<br><br>- monitored arrest (2.23, 1.41 to 3.52, high certainty),<br><br>- arrest during daytime hours (1.41, 1.20 to 1.66, high certainty),<br><br>- initial shockable rhythm (5.28, 3.78 to 7.39, high certainty).<br><br>Intra-arrest factors associated with reduced odds of survival: | Prognostic/ predictive value confirmed (10 factors) |

| | | | | | | |
|---|---|---|---|---|---|---|
| | | | | | - intubation during arrest (0.54, 0.42 to 0.70, moderate certainty)<br><br>- duration of resuscitation of at least 15 minutes (0.12, 0.07 to 0.19, high certainty). | |
| 52. Glasmacher et al. 2019 | c9orf72RE disorders, including amyotrophic lateral sclerosis (ALS) and frontotemporal dementia (FTD) | 206 | 1060 | prognostic factors in c9ALS, c9FTD, c9ALS-FTD, and atypical phenotypes | "The median (95% CI) survival (in years) differed significantly between patients with c9ALS (2.8 [2.67-3.00]), c9FTD (9.0 [8.09-9.91]), and c9ALS-FTD (3.0 [2.73-3.27]); survival in atypical phenotypes varied substantially. Older age at onset was associated with shorter survival in c9ALS (HR, 1.03; 95% CI, 1.02-1.04; P < .001), c9FTD (HR, 1.04; 95% CI, 1.02-1.06; P < .001), and c9ALS-FTD (HR, 1.02; 95% CI, 1.004-1.04; P = .016). Bulbar onset was associated with shorter survival in c9ALS (HR, 1.64; 95% CI, 1.27-2.08; P < .001). Age at onset and bulbar onset ALS remained significant in multivariable regression including variables indicating potential diagnostic ascertainment bias, selection bias, and reporting bias. Family history, sex, study continent, FTD subtype, or the presence of additional pathogenic sequence variants were not significantly associated with survival. Clinical phenotypes in patients with neuropathologically confirmed frontotemporal lobar degeneration-TDP-43, motor neuron disease-TDP-43 and frontotemporal lobar degeneration-motor neuron disease-TDP-43 were heterogenous and impacted on survival." | Prognostic/ predictive value of small set of parameters confirmed, highly specific context of condition and/or therapy |
| 53. Graizel et al. 2019 | oral squamous cell carcinoma | 11 | 1040 | cancer-associated fibroblasts (CAFs) | "Univariate Cox regressions showed that high CAF density was a negative prognostic factor in | Single marker (i.e. relative |

| | | | | | | |
|---|---|---|---|---|---|---|
| | | | | | studies with female and male majority [OR 5.329 (95% CI 3.223-8.811), p < 0.001, and OR 2.208 (95% CI 1.717-2.839), p < 0.001, respectively]. High CAF density with male majority was associated with a more favorable prognosis [OR 0.996 (95% CI 0.979-1.013), p < 0.001]. Multivariate Cox regressions showed that death risk was significantly higher among patients with high CAF density compared to low CAF [OR 2.741 (95% CI 2.220-3.384) p < 0.001]. High mean age and male proportion were significantly protective [OR 0.940 (95% CI 0.925-9.955), p < 0.001, OR 0.125 (95% CI 0.018-0.867), p = 0.035), respectively]. CONCLUSIONS: CAFs increased death risk, male majority, and higher mean age were protective. A clinically validated cutoff for CAF density could serve as a reliable prognostic tool." | importance unclear), prognostic/ predictive value confirmed |
| 54. He et al. 2019 | colorectal cancer with surgical resection | 83 original prediction models and 52 separate external validation studies were identified | | Prediction models | "We identified five models (Basingstoke score, Fong score, Nordinger score, Peritoneal Surface Disease Severity Score and Valentini nomogram) that were validated in at least two external datasets with a median summarized C-statistic of 0.67 (range: 0.57-0.74). These models can potentially assist clinical decision-making. Besides developing new models, future research should also focus on validating existing prediction models and investigating their real-word impact and cost-effectiveness for CRC prognosis in clinical practice." | Most models not suitable for clinical use; further validation/ updating required |
| 55. Jiang et al. 2019 | non-small cell lung cancer (NSCLC) | 10? (abstract ambiguous) | | circulating tumor cells (CTCs) prior to initial treatment | "Randomized model analyzing multivariate Cox Proportional Hazards Regression indicated that higher abundance of CTCs significantly predicts poorer prognosis of lung cancer cases basing | Single marker (i.e. relative importance unclear), |

| | | | | | | |
|---|---|---|---|---|---|---|
| | | | | | both on PFS (Z = 2.31, P = 0.02) and OS of advanced cases (Z = 2.44, P = 0.01), and systematic study also indicated the similar results.<br>CONCLUSION: High CTCs prior to initial treatment can predict shorter PFS and OS in NSCLC, and further studies are warranted in the future." | prognostic/ predictive value confirmed, further research recommended |
| 56. Li S et al. 2019 | Breast cancer | 19 | 12,505 | programmed cell death ligand-1 expression | "PD-L1 expression was significantly associated with lymph node metastasis (P < .001), high tumor grade (P < .001), negative hormone receptor (P < .001), human epidermal growth factor receptor 2 (HER2) positivity (P < .001), high Ki67 (P < .001), and high tumor-infiltrating lymphocytes (TILs) (P < .001). PD-L1 expression had no significant impact on CSS (pooled HR 0.83, 95% CI = 0.64-1.09, P = .19) or MFS (pooled HR 1.11, 95% CI = 0.62-1.97, P = .72), but significantly correlated with shortened OS (pooled HR 1.52, 95% CI = 1.14-2.03, P = .004) and DFS (pooled HR 1.31, 95% CI = 1.14-1.51, P < .000). Subgroup analysis showed that not PD-L1 RNA expression, but protein expression was associated with shorter survival, in addition, the adverse prognostic effect of PD-L1 expression remained in luminal A, luminal B, and HER2 subtype, not in basal-like or triple-negative subtype." | Single marker (i.e. relative importance unclear), prognostic/ predictive value confirmed |
| 57. Li W et al. 2019 | brain metastasis in non-small cell lung cancer | 18 | 4373 | mutation of the epidermal growth factor receptor (EGFR) | "Mutated EGFR associated with significantly improved OS compared with wild type. Subgroup analyses suggested that this relationship persisted in studies conducted in Eastern, with retrospective design, with sample size >=500, mean age of patients >=65.0 years, | Single marker (i.e. relative importance unclear), prognostic/ |

| | | | | | | |
|---|---|---|---|---|---|---|
| | | | | | percentage male < 50.0%, percentage of patients receiving tyrosine kinase inhibitor >=30.0%. Finally, although significant publication bias was observed using the Egger test, the results were not changed after adjustment using the trim and fill method. CONCLUSIONS: This meta-analysis suggests that EGFR mutation is an important predictive factor linked to improved OS for NSCLC patients with brain metastases. It can serve as a useful index in the prognostic assessment of NSCLC patients with brain metastases." | predictive value confirmed |
| 58. Mierzynska et al. 2019 | cancer | 44 | | baseline patient-reported outcomes (PROs) | "Of the 44 studies published between 2006 and 2018 that were included in our review, more standardisation and rigour of the methods used for prognostic factor analysis was found compared with the previous review. 41 (93%) of the trials reported at least one PRO domain as independently prognostic. The most common significant prognostic factors reported were physical functioning (17 [39%] studies) and global health or quality of life (15 [34%] studies). These findings highlight the value of PROs as prognostic or stratification factors in research across most types of cancer." | PROMS (no new information to patients) |
| 59. Pergialiotis et al. 2019 | Ovarian cancer | 11<br>(5 in meta-analysis) | 1816<br>(856 in meta-analysis) | prechemotherapy hemoglobin (Hgb) levels | "Compared with patients with anemia, patients with Hgb levels >12 g/dL had increased odds of overall survival (odds ratio, 1.72; 95% confidence interval: 1.41, 2.10)."<br><br>"Current evidence suggests that prechemotherapy Hgb levels below the threshold of 12 g/dL can potentially predict worse overall survival of OC patients. Future research is required in the field to elucidate | Single marker (i.e. relative importance unclear), prognostic/ predictive value confirmed, further research recommended |

| | | | | | | |
|---|---|---|---|---|---|---|
| | | | | | whether several independent variables such as the stage and histology of disease and rates of optimal debulking affect the clinical significance of this association.” | |
| 60. Rutherford et al. 2019 | colorectal cancer (CRC) | 27 | 12,544 (unclear from abstract if refers to all 27 or the 25 studies which find association with survival) | Patient-reported outcomes | In 25 of 27 studies (n = 12,544), at least one PRO was significantly associated with survival. Physical functioning, fatigue, pain and appetite loss predicted OS more often than other PROs in metastatic disease (19/27 studies). One study explored PRO predictors in early-stage CRC, finding emotional well-being and mood predicted OS. In mixed-stage samples (7/27 studies), physical functioning predicted OS more often than other PROs.”<br><br>“Physical and psychological functioning, pain, fatigue and appetite loss had prognostic significance above and beyond clinical predictors in CRC.” | PROMS (no new information to patients) |
| 61. Sabarimurugan et al. 2019 | Nasopharyngeal cancer (NPC) | 21 | 5069 | miRNAs | “The forest plot was generated using cumulated survival data, resulting in a pooled HR value of 1.196 (95% CI: 0.893-1.601) indicating that the upregulated miRNAs increased the likelihood of death of NPC patients by 19%.”<br><br>“[T]he combined effect estimate of HR across multiple studies indicated that increased miRNA expression in NPC potentially leads to poor overall survival. However, further large-scale prospective studies on the clinical significance of the miRNAs, with sizable cohorts are necessary in order to obtain conclusive results.” | Single marker (i.e. relative importance unclear), prognostic/ predictive value confirmed, further research recommended |

| | | | | | | |
|---|---|---|---|---|---|---|
| 62. Sun et al. 2019 | Colorectal cancer | 10 | 6372 | preoperative prognostic nutritional index (PNI) | “Our overall analysis indicated that the low-PNI group had a significantly reduced overall survival (OS) (HR = 1.87, 95% CI = 1.45-2.42, P < 0.01), cancer-specific survival (HR = 1.53, 95% CI = 1.07-2.19, P = 0.02), and disease-free survival (HR = 1.67, 95% CI = 1.23-2.26, P < 0.01) compared with the high-PNI group. Furthermore, our subgroup results indicated that a high PNI could be a significant indicator of improved OS in TNM stage II (HR = 1.93, 95% CI = 1.29-2.90, P < 0.01) and III (HR = 1.71, 95% CI = 1.25-2.34, P < 0.01), and a similar trend in TNM stage I or IV could also be observed though without statistical significance. Regarding postoperative complications, our pooled results indicated that the low-PNI group had a significantly increased incidence of total and severe postoperative complications. CONCLUSIONS: Our findings indicated that CRC patients with a preoperative high PNI had a significantly improved OS. However, almost only Asian CRC patients were included based on current issue.” | Single marker (i.e. relative importance unclear), prognostic/ predictive value confirmed, limitations of included studies (i.e. further research required) |
| 63. Wang M et al. 2019 | cancer | 6 | 1584 | TRIM59 | “The results showed that high levels of TRIM59 were significantly associated with poor OS in cancer patients (HR = 1.43, 95%CI: 1.24-1.66, P < .001), indicating that higher TRIM59 expression could be an independent prognostic factor for poor survival in cancer patients. CONCLUSION: Our meta-analysis suggests that higher TRIM59 expression predicts poor prognosis in cancer patients, and it may therefore serve as a promising prognostic factor.” | Single marker (i.e. relative importance unclear), prognostic/ predictive value confirmed |

| | | | | | | |
|---|---|---|---|---|---|---|
| 64. Wang SJ et al. 2019 | repeat hepatectomy for recurrent colorectal liver metastasis (CRLM) | 34 | 3039 | Prognostic factors as identified in the literature | "Pooled analysis showed that primary T3/T4 stage tumor (HR = 1.94; 95% CI: 1.04-3.63), multiple tumors (HR = 1.49; 95% CI: 1.10-2.01), largest liver lesion >=5cm (HR = 1.89; 95% CI: 1.11-3.23) and positive surgical margin (HR = 1.80; 95% CI: 1.09-2.97) at initial hepatectomy, and high serum level of carcinoembryonic antigen (HR = 1.87; 95% CI: 1.27-2.74), disease-free interval <=12 months (HR = 1.34; 95% CI: 1.10-1.62), multiple tumors (HR = 1.64; 95% CI: 1.32-2.02), largest liver lesion >=5cm (HR = 1.85; 95% CI: 1.34-2.56), positive surgical margin (HR = 2.25; 95% CI: 1.39-3.65), presence of bilobar disease (HR = 1.62; 95% CI: 1.19-2.20), and extrahepatic metastases (HR = 1.60; 95% CI: 1.23-2.09) at repeat hepatectomy were significantly associated with poor OS. CONCLUSIONS: Repeat hepatectomy is a safe and effective therapy for recurrent CRLM. Long-term outcome is predicted mainly by factors related to repeat hepatectomy." | Prognostic/ predictive value confirmed (11 factors) |
| 65. Wang X and Wang Y, 2019 | Gynaecological cancer | 9 | 2373 | prognostic nutritional index (PNI) | "The PNI correlated closely with the OS and PFS of gynecological cancer; the pooled HRs were respectively 2.66 (95% CI 1.56-4.55) and 2.43 (95% CI 2.07-2.86) on univariate analysis (UVA) and 1.88 (95% CI 1.10-3.20) and 1.92 (95% CI 1.52-2.44) on multivariate analysis (MVA). Conclusions: The PNI is significantly associated with the prognosis of patients with gynecological cancer, and may, in fact, be independently prognostic." | Single marker (i.e. relative importance unclear), prognostic/ predictive value confirmed |
| 66. Yang C et al. 2019 | Patients with malignant solid tumors | 17 | 2,228 | T-cell lymphoma invasion and | "The overall estimated results showed that high Tiam1 expression was significantly associated with shorter overall survival (HR=2.08, 95% CI: | Single marker (i.e. relative importance |

| | | | | | | |
|---|---|---|---|---|---|---|
| | | | | metastasis inducing factor 1 (Tiam1) | 1.62-2.68, P<0.01), and disease-free survival (HR=1.86, 95% CI: 1.49-2.32, P<0.01). Besides, we also found that there was a close relationship between high Tiam1 expression and positive lymphatic metastasis (OR=2.63; 95% CI: 1.79-3.84, P<0.01).<br>Conclusion: High Tiam1 expression was significantly associated with shorter survival and positive lymphatic metastasis in patients with malignant solid tumors. Therefore, Tiam1 may be a promising prognostic biomarker and an effective therapeutic target for malignant solid tumors." | unclear), prognostic/ predictive value confirmed |
| 67. Yang L et al. 2019 | nasopharyngeal carcinoma (NPC) | 21 | 2921 | Excision repair cross-complementation group 1 (ERCC1) protein | "The pooled results showed that high/positive expression of ERCC1 predicted poor objective response rate (ORR) [odds ratio (OR) = 2.83; 95% confidence interval (CI) = 2.11-3.80; P <.001], overall survival (OS) [hazard ratio (HR) = 1.77; 95% CI = 1.48-2.12; P <.001], and disease-free survival (DFS) (HR = 1.60; 95% CI = 1.43-1.79; P <.001) in NPC. Low heterogeneity was detected among these studies (ORR: I = 0.0%, P = .776; DFS: I = 38.7%, P = .148; OS: I = 0.0%; P = .530). The results of sensitivity analyses and publication bias verified the reliability of our findings.<br>CONCLUSIONS: This study suggested ERCC1 as a potential predictive and prognostic biomarker for the treatment response and survival prognosis of NPC patients." | Single marker (i.e. relative importance unclear), prognostic/ predictive value confirmed |
| 68. Yang M et al. 2019 | Lung cancer | 13 | 1,810 | sarcopenia (skeletal muscle depletion) | "The pooled prevalence of sarcopenia was 43% in patients with non-small cell lung cancer (NSCLC) and 52% in patients with small cell lung cancer (SCLC). Sarcopenia was associated with | Single marker (i.e. relative importance unclear), |

| | | | | | | |
|---|---|---|---|---|---|---|
| | | | | | a shorter overall survival (OS) in patients with lung cancer (hazard ratio [HR], 2.23; 95% CI, 1.68-2.94). This association existed for both NSCLC (HR, 2.57; 95% CI, 1.79-3.68) and SCLC (HR, 1.59; 95% CI, 1.17-2.14). Sarcopenia was an independent predictor of shorter OS in both stage I-II NSCLC (HR, 3.23; 95% CI, 1.68-6.23) and stage III-IV NSCLC (HR, 2.19; 95% CI, 1.14-4.24). However, sarcopenia was not an independent predictor of disease-free survival in patients with NSCLC (HR, 1.28; 95% CI, 0.44-3.69). Conclusions: Sarcopenia is highly prevalent in patients with lung cancer (approximately one in two) and an important predictor of impaired OS in patients with SCLC or with different stages of NSCLC." | prognostic/ predictive value confirmed |
| 69. Yu et al. 2019 | Liver failure | 13 | 2071 | hepatic encephalopathy | "The results proved the prognostic value of hepatic encephalopathy for survival of patients with liver failure (OR=5.62, 95%CI=6.30-9.82, P<0.001). The subgroup analyses showed that the type of liver failure and the follow up duration may be the factor influencing the association between hepatic encephalopathy and survival of patients with liver failure." | Single marker (i.e. relative importance unclear), prognostic/ predictive value confirmed |
| 70. Cao et al. 2020 | Gastric cancer | 28 | 15,617 | platelet-to-lymphocyte ratio (PLR) | "The pooled results indicated that elevated PLR was associated with poor OS (HR: 1.37; 95% CI: 1.24-1.51; P < 0.001). A significant publication bias was observed (Egger test, P = 0.036; Begg test, P = 0.017). After adjusting for publication bias using the trim and fill method, an adjusted pooled HR of 1.19 (95% CI: 1.08-1.33; P = 0.001) was observed. Subgroup analyses indicated an elevated PLR in retrospective studies. Studies conducted in Turkey, the UK, the USA, and | Single marker (i.e. relative importance unclear), prognostic/ predictive value confirmed, limitations of included studies (i.e. further |

| | | | | | | |
|---|---|---|---|---|---|---|
| | | | | | Costa Rica; studies with a sample size of < 1000, with < 70% male patients, and with patients treated with chemotherapy; studies with PLR cutoff value of >=200; and studies with lower quality as determined by the Newcastle-Ottawa Scale all showed greater harmful effects on OS than their corresponding subsets (P < 0.05). CONCLUSIONS: An elevated PLR was associated with poor OS in patients with gastric cancer. These results might differ between studies due to differences in design, country of origin, sample size, sex proportion, treatment strategy, PLR cutoff value, and study quality.” | research required) |
| 71. Zhao et al. 2020 | gastric cancer | 13 | 7004 | Perineural invasion | “There were significant relationships between PNI and a series of unfavourable clinicopathological factors including undifferentiated histology type (OR: 1.78, 95% CI 1.37 to 2.33, p<0.001; I<sup>2</sup>=75.3%), diffuse type (OR: 1.96, 95% CI 1.07 to 3.60, p=0.029; I<sup>2</sup>=79.5%), lymphatic invasion (OR: 7.00, 95% CI 3.76 to 13.03, p<0.001; I<sup>2</sup>=83.6%), vascular invasion (OR: 5.79, 95% CI 1.59 to 21.13, p=0.008; I<sup>2</sup>=95.8%), deeper tumour invasion (OR: 4.79, 95% CI 3.65 to 6.28, p<0.001; I<sup>2</sup>=65.0%) and lymph node metastasis (OR: 3.60, 95% CI 2.37 to 5.47, p<0.001; I<sup>2</sup>=89.6%). In addition, PNI was significantly associated with worse survival outcome in GC patients (HR: 1.69, 95% CI 1.38 to 2.06, p<0.001; I<sup>2</sup>=71.0%).” | Single marker (i.e. relative importance unclear), prognostic/ predictive value confirmed |
